# Single-Stage Causal Incentive Design via Optimal Interventions

**DOI:** 10.3390/e28010004

**Published:** 2025-12-19

**Authors:** Sebastián Bejos, Eduardo F. Morales, Luis Enrique Sucar, Enrique Munoz de Cote

**Affiliations:** 1 Computer Science Department, National Institute of Astrophysics, Optics and Electronics, Puebla 72840, Mexico; emorales@inaoep.mx (E.F.M.); esucar@inaoep.mx (L.E.S.); 2 Mutable Tactics, London CB2 9PJ, UK; enrique@mutabletactics.ai

**Keywords:** causal inference, applications of causal graphical models, incentive design, principal–agent problems, hierarchical Stackelberg games, Bayesian optimization, Gaussian process, information gain, differential entropy, regret analysis

## Abstract

We introduce Causal Incentive Design (CID), a framework that applies causal inference to canonical single-stage principal–agent problems (PAPs) characterized by bilateral private information. Within CID, the operating rules of PAPs are formalized using an additive-noise causal graphical model (CGM). Incentives are modeled as interventions on a function space variable, Γ, which correspond to policy interventions in the principal–follower causal relation. The causal inference target estimand V(Γ) is defined as the expected value of the principal’s utility variable under a specified policy intervention in the post-intervention distribution. In the context of additive-Gaussian independent noise, the estimand V(Γ) decomposes into a two-layer expectation: (i) an inner Gaussian smoothing of the principal’s utility regression; and (ii) an outer averaging over the conditional probability of the follower’s action given the incentive policy. A Gauss–Hermite quadrature method is employed to efficiently estimate the first layer, while a policy-local kernel reweighting approach is used for the second. For offline selection of a single incentive policy, a Functional Causal Bayesian Optimization (FCBO) algorithm is introduced. This algorithm models the objective functional γ↦V(γ) using a functional Gaussian process surrogate defined on a Reproducing Kernel Hilbert Space (RKHS) domain and utilizes an Upper Confidence Bound (UCB) acquisition functional. Consequently, the policy value V(γ) becomes an interventional query that can be answered using offline observational data under standard identifiability assumptions. High-probability cumulative-regret bounds are established in terms of differential information gain for the proposed FBO algorithm. Collectively, these elements constitute the central contributions of the CID framework, which integrates causal inference through identification and estimation with policy search in principal–agent problems under private information. This approach establishes a causal decision-making pipeline that enables commitment to a high-performing incentive in a single-shot game, supported by regret guarantees. Provided that the data used for estimation is sufficient, the resulting offline pipeline is appropriate for scenarios where adaptive deployment is impractical or costly. Beyond the methodological contribution, this work introduces a novel application of causal graphical models and causal reasoning to incentive design and principal–agent problems, which are central to economics and multi-agent systems.

## 1. Introduction

In an incentive design (ID) problem, a central institution alters the behavior of self-interested agents to improve overall performance by implementing an incentive function that adjusts their individual payoffs. The central institution, typically represented as a principal agent, may aim to drive the system’s performance towards a more desirable behavior, such as maximizing revenue or social welfare within the multi-agent system. This work concentrates on the single-stage canonical principal–agent problem (PAP), in which an incentive designer agent (the principal) commits to an incentive function that influences the behavior of a single follower. The interaction is a one-shot hierarchical inverse Stackelberg game, in which the principal first announces an incentive function; the follower then responds optimally based on that commitment, and the outcomes are realized without any subsequent adaptation or additional rounds. We maintain the assumption of private information for both players; specifically, the principal is unaware of the follower’s utility function and vice versa.

The single-stage PAP is the canonical problem in incentive design (ID) because it includes the core structural features in a one-shot setting: the principal commits once to an incentive, the follower best-responds, and outcomes are realized; information asymmetry and incomplete knowledge persist; and strategic reasoning is driven by ex ante beliefs about utilities or types. While there is no intertemporal adaptation or belief updating between rounds, the principal–agent (leader–follower) tension remains fundamental: one party creates incentives in the face of uncertainty, while the other optimizes behavior in response to that commitment. These features remain structurally invariant when extending the single-period formulation to multiple followers and multiple principals. With private information on both sides, the design task still entails bilateral inference at design time—reasoning about the other party’s unknown objectives—which is the core difficulty that scales to richer multi-agent settings. Thus, the single-stage formulation preserves the irreducible skeleton of ID problems and the central epistemic and incentive-alignment challenges that govern their multi-agent variants [1].

Consider a lender as the principal and a borrower as the follower. The lender must choose, once, a contract rule—an incentive function that maps borrower attributes or application information to loan terms (e.g., interest rate, credit limit, collateral, or a repayment rebate). The borrower then decides whether to accept the contract and, if funded, how much effort to exert toward repayment. Both sides hold private information (e.g., the borrower’s risk and effort costs, and the lender’s risk appetite and cost structure). The outcome—acceptance, repayment, or default, and the lender’s realized payoff—occurs once; there is no opportunity to update terms after observing behavior. This one-shot contract choice is exactly a single-stage PAP: the lender designs the incentive rule under uncertainty; the borrower best-responds; the realized payoff reflects both the selection induced by the rule and the behavioral response it elicits.

We treat the principal’s single-stage choice as the endpoint of a finite-horizon sequential optimization carried out before deployment. The principal starts from historical observations of the system—past incentive rules, the follower’s responses, and realized payoffs—and uses them to estimate the principal’s expected utility under any candidate incentive function. A data-efficient search procedure (Bayesian optimization over a space of incentive functions) iteratively proposes candidate incentives, updates beliefs about expected utility using the accumulated evidence, and records the most promising options. After a fixed number of iterations, the principal selects one incentive function—the estimated best—to implement in the single-stage game. This procedure makes no restrictive assumptions about the players’ utility forms and respects the private-information setting; it is simply a principled way to use prior observations to select a high-value one-shot incentive.

### 1.1. Approach and Contributions

Even in the single-stage canonical principal–agent problem (PAP), it is difficult to predict how a committed incentive will affect the follower’s behavior under bilateral private information. We address this challenge by integrating causal inference with incentive design: we represent the single-stage PAP as a causal graphical model (CGM) in which the incentive function is treated as an intervention on the function space variable Γ, and we target the principal’s utility variable $J_{L}$ under that policy, $V\left(\gamma \right)\equiv E[J_{L}|\mathrm{do}\left(\Gamma =\gamma \right)]$. This causal target is identified via the *g*-formula, enabling estimation of policy values from observational data without requiring controlled experiments. We then cast policy selection as a black-box sequential offline optimization over a function space of incentives, using Bayesian optimization to efficiently search among costly evaluations of V(γ) before committing to a single policy in the one-shot game.

In the proposed framework, the leader’s policy Γ acts as a functional intervention, the follower’s action is an explicit mediator, and the leader’s payoff $J_{L}$ is the outcome node. The policy value $V\left(\gamma \right)=E[J_{L}|do\left(\Gamma =\gamma \right)]$ constitutes an interventional and counterfactual query on this causal graphical model, which can be identified and estimated from observational data (offline logs) under the assumptions of positivity and additive noise. Accordingly, this work advances causal reasoning with graphical models and introduces a novel application domain in incentive design and principal–agent models, thereby complementing established applications in biology, medicine, and economics.

We formalize the single-stage canonical PAP (one principal and one follower) as an additive-noise CGM with endogenous variables $\left\{\Gamma ,\Omega_{L},\Omega_{F},J_{F},J_{L}\right\}$ that capture the inverse-Stackelberg semantics $\Omega_{L}=\gamma \left(\Omega_{F}\right)+U_{L}$, where incentives are interventions do(Γ=γ). From this model, we derive a non-parametric identification of V(γ) by marginalizing over $\Omega_{L}$ and $\Omega_{F}$. In the additive-Gaussian setting, the estimand becomes a nested expectation with two interpretable components: an inner expectation (a Gaussian smoothing of the outcome regression $\mu_{L}\left(\Omega_{L},\Omega_{F},\Gamma \right)$) and an outer expectation over the mediator law $p(\Omega_{F}|\Gamma =\gamma )$ induced by the incentive. Practically, we compute the inner term by Gauss–Hermite quadrature and approximate the outer term by policy-local kernel reweighting in policy space; a linear credit-market example (affine pricing rule) illustrates a closed-form specialization.

We optimize γ↦V(γ) over an admissible set Γ modeled as a Reproducing Kernel Hilbert Space (RKHS) $H_{k}$. A functional Gaussian process (GP) surrogate on $H_{k}$—with a functional RBF kernel driven by RKHS distances between incentive functions—provides posterior mean and uncertainty over policy values. A functional GP-UCB acquisition functional trades off exploration and exploitation to propose the next incentive to evaluate, building a data set ${\left\{\left(\gamma^{t},\hat{V}\left(\gamma^{t}\right)\right)\right\}}_{t=1}^{T}$ from which we select the best $\gamma^{+}$ to deploy in the single-stage game. We provide high-probability cumulative-regret bounds for this Stackelberg Functional Causal Bayesian Optimization (FCBO) procedure in terms of *differential information gain*, with guarantees for both finite policy sets and infinite (non-parametric) RKHS domains.

**Figure 1:**
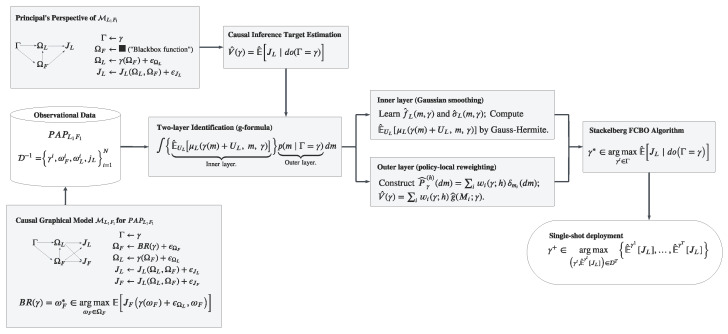
Overview of the CID pipeline. Identification yields a two-layer estimand; the estimator feeds a functional GP surrogate and a support-aware GP-UCB search to select a single policy offline. Shaded boxes indicate contributions introduced in this paper.

**Figure 2:**
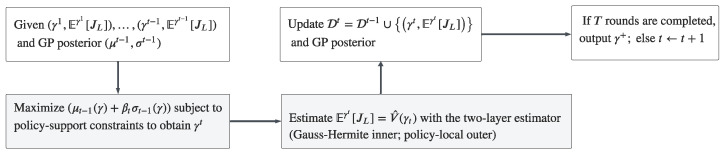
One iteration of Stackelberg FCBO algorithm. Novel elements are highlighted with shaded boxes: support-aware GP-UCB selection and the nested estimator used for off-policy evaluation.

Our pipeline builds on established ingredients: causal identification via the g-formula and policy interventions [2,3], RKHS/GP surrogates for Bayesian optimization [4,5,6], and GP-UCB-style exploration with information-gain regret analysis [7,8]. We have four new key contributions: (i) a PAP-specific CGM that treats incentives as functional interventions and makes the follower action an explicit mediator of policy value; (ii) a two-layer identification and estimation recipe for single-stage PAPs, using Gaussian inner smoothing with policy-local outer reweighting; (iii) a functional GP surrogate on an RKHS of incentive rules with a policy-space RBF and support-aware acquisition; and (iv) regret bounds stated in terms of differential information gain for the Stackelberg functional setting with a uniform sub-Gaussian envelope for estimator noise. Figure 1 sketches the full CID pipeline (identification and estimation of V(γ) and FCBO policy search) and Figure 2 depicts one FCBO iteration; shaded boxes on Figure 1 and Figure 2 mark components introduced in this paper. Our contributions can be summarized as follows:

- **CID for single-stage PAPs.** We establish a generic CGM for the single-stage canonical PAP in which incentives are formalized as functional interventions, yielding a principled causal estimand for policy value $V\left(\gamma \right)=E[J_{L}|\mathrm{do}\left(\Gamma =\gamma \right)]$ identified from observational data via the *g*-formula.
- **Semi-parametric estimation strategy.** We develop a practical, modular estimator for V(γ) under additive Gaussian noise: (a) learn the principal’s action mechanism and outcome regression; (b) compute the inner Gaussian expectation by Gauss–Hermite quadrature; and (c) approximate the induced mediator law with policy-local kernel weights. A linear credit-market pricing example provides a closed form that clarifies the roles of policy parameters and the induced borrowing response.
- **Functional Bayesian optimization for incentives.** We propose the *Stackelberg FCBO* algorithm: a functional GP surrogate on $H_{k}$ with a functional GP-UCB acquisition functional to sequentially (offline) optimize over incentive functions when policy evaluations are expensive and noisy.
- **Theoretical guarantees.** We prove high-probability cumulative-regret bounds that scale with $\sqrt{T\;\beta_{T}\;I_{T}}$, where $I_{T}$ is the information gain and $\beta_{T}$ is the exploration schedule. We extend the analysis from finite admissible sets to infinite RKHS domains via covering arguments and uniform approximation—quantifying the data-efficiency and reliability of the offline design that precedes the one-shot deployment.

### 1.2. Related Work

Incentive design (ID) has long been studied in economics and control through the lens of principal–agent theory [9,10]. Within this tradition, the single-stage (one-shot) PAP is a canonical formulation of contracting under asymmetric information: the principal commits to a contract, the agent best-responds, and outcomes are realized once. Classical approaches model uncertainty with priors over types and often assume a known mapping from actions to outcomes, yielding foundational results in adverse selection and moral hazard [1,9]. Control-theoretic treatments similarly emphasize leader–follower structure but typically rely on parametric models of dynamics and payoffs (e.g., early closed-loop Stackelberg formulations and multi-level control [10], and modern bilevel optimization theory and hardness results [11]).

More recently, the machine learning literature has approached ID through online learning paradigms—such as multi-armed bandits, reinforcement learning, and Bayesian optimization [5,12]—where the principal adapts incentives across rounds while observing partial feedback. This work has produced algorithms for repeated principal–agent settings, combinatorial bandits for dynamic preferences [13,14], meta-gradient methods for adaptive mechanism design [12,15], and BO-based procedures that steer multi-agent systems toward desirable equilibria [16]. While powerful, these approaches are inherently multi-stage and often treat the environment as a black box, emphasizing empirical adaptation rather than explicit reasoning about how a committed incentive causes behavioral change in a one-shot deployment. Beyond incentive design, robustness studies in multilayer transportation networks similarly demonstrate that inter-layer coupling critically governs aggregate performance—see the bilayer railway–aviation analysis with discrete cross-layer assignment [17].

A parallel line of research integrates causal reasoning into sequential decision-making [18,19,20,21]. Causal bandits, counterfactual policy evaluation, and causal reinforcement learning utilize graphical models to structure exploration and target the effects of interventions [22,23]. In Causal Bayesian Optimization (CBO) [24], the optimization routine exploits known causal structure among inputs to identify high-value interventions more efficiently. These methods, however, typically consider atomic (variable-level) interventions and multi-round interactions. In contrast, our focus is on a one-shot commitment problem: the principal must select a single incentive function before deployment and cannot adapt it afterward.

Our work connects these strands by introducing a causal, offline design pipeline tailored to the single-stage PAP. We represent the principal–agent interaction with a causal graphical model (CGM) and interpret incentives as functional (conditional) interventions and policies as interventions on the node that embodies the principal’s rule. This yields a policy value target for the principal (the expected utility under the post-intervention distribution) that can be identified from observational data via the g-formula and estimated without requiring online experimentation. To efficiently search over a space of incentive functions before deployment, we adopt a Functional Bayesian Optimization procedure [6,25]: a functional Gaussian process surrogate on an RKHS of admissible incentives, coupled with a UCB-type acquisition functional, guides a small number of costly offline evaluations of policy value. Taken together, the proposed approach complements classical single-stage contract theory by supplying a causal identification–estimation pathway for policy value under private information. Finally, our analysis provides high-probability regret guarantees in terms of differential information gain, quantifying how efficiently the offline procedure learns the principal’s objective over the function space.

Our emphasis is on the *theoretical underpinnings* of single-stage causal incentive design, specifically, (i) identification of policy value under functional interventions; (ii) a two-layer estimation approach with policy-local support diagnostics; and (iii) support-aware Functional Bayesian Optimization with regret control under a uniform sub-Gaussian envelope. To maintain focus and adhere to space constraints, we reserve numerical simulations and applied case studies for subsequent work that will use the present framework as a baseline to explore design choices, including policy classes, kernel metrics, and bandwidth selection across various domains.

### 1.3. Outline

The rest of the paper is organized as follows. The preliminary background, Section 2, first reviews the bilevel viewpoint of hierarchical Stackelberg games and inverse Stackelberg formulations. We then formalize the single-stage principal–agent problem (SS-PAP) and specify the information model with bilateral private information. The background closes with the essentials of causal graphical models (CGMs) and the notions of hard and functional (policy) interventions used later for identification and estimation. We introduce our Causal Incentive Design (CID) framework for canonical SS-PAPs in Section 3. We present an additive-noise Gaussian CGM for the canonical single-stage PAP and its assumptions, define the principal’s policy value target estimand $V\left(\gamma \right)=E[J_{L}|\mathrm{do}\left(\Gamma =\gamma \right)]$, and provide a semi-parametric identification formula for V(γ) via the g-formula. We then describe a practical offline estimation pipeline for the identification formula in general and provide an illustrative credit-market example with an affine pricing rule that demonstrates a closed-form specialization of the estimand. Section 4 details the *Stackelberg FCBO* procedure for offline policy selection. We model the objective γ↦V(γ) with a functional Gaussian-process surrogate over an RKHS of admissible incentives and employ a functional GP-UCB acquisition functional. We then present the Stackelberg FCBO algorithm and provide high-probability cumulative-regret bounds in terms of differential information gain. Section 5 reflects on the implications of CID for single-shot incentive design and outlines directions for empirical validation and broader multi-agent extensions.

## 2. Preliminary Background

The background is organized into three components: (i) the Stackelberg and inverse Stackelberg perspectives for incentive design (Section 2.1); (ii) the single-stage principal–agent problem (PAP) setting and the information model for the single-stage PAP with bilateral private information (Section 2.2); and (iii) the fundamentals of causal graphical models (CGMs), hard interventions, and functional (policy) interventions, which serve as the foundation for interpreting incentives as policies (Section 2.3).

### 2.1. Incentive Design as Inverse Stackelberg Games

Games characterized by a hierarchical decision-making structure, where one or more players, called the leaders, declare their strategy first and impose this strategy upon the other players, called the followers, are referred to as Stackelberg games. If leaders declare their strategy as a mapping from the followers’ decision space into their own decision space, we refer to this as inverse Stackelberg games. The essence of a Stackelberg game can be described considering the basic single-leader single-follower single-stage game as follows. Let us denote by $\omega_{L}\in \Omega_{L}\subseteq R^{n_{L}}$ and $\omega_{F}\in \Omega_{F}\subseteq R^{n_{F}}$ the leader and follower decision variables, respectively. The leader chooses an action or decision from its decision space $\Omega_{L}$, and the follower, informed of the leader’s choice, subsequently chooses an action from its decision space $\Omega_{F}$. Each player aims to maximize its utility functions $J_{L}$: $\Omega_{L}\times \Omega_{F}\to R$ and $J_{F}$: $\Omega_{L}\times \Omega_{F}\to R$, both functions of the decision spaces $\Omega_{L}$ and $\Omega_{F}$.

Since the leader acts first and announces his decision $\omega_{L}$, which is subsequently made known to the follower, the follower’s action becomes a function of the leader’s action $\omega_{F}=r_{F}\left(\omega_{L}\right)$. The function $r_{F}$: $\Omega_{L}\to \Omega_{F}$ is called the reaction function of the follower (as it indicates how the follower will react to the leader’s decision). Knowing $\omega_{L}$, the follower chooses $\omega_{F}^{*}$, with 

$$\omega_{F}^{*}\in \underset{\omega_{F}\in \Omega_{F}}{\arg\max}\;J_{F}\left(\omega_{L},\omega_{F}\right)=J_{F}\left(\omega_{L},r_{F}\left(\omega_{L}\right)\right).$$

Taking into account the aforementioned, before the leader announces his decision $\omega_{L}$, he will realize how the follower will react and hence based on this knowledge, the leader will choose and subsequently announce the leader’s optimal decision $\omega_{L}^{*}\in \Omega_{L}$, with 

$$\omega_{L}^{*}\in \underset{\omega_{L}\in \Omega_{L}}{\arg\max}\;J_{L}\left(\omega_{L},r_{F}\left(\omega_{L}\right)\right).$$

In contrast to the original Stackelberg game discussed above, in the inverse Stackelberg game, the leader acts first by deciding and announcing an incentive function γ to the follower, instead of $\omega_{L}$. This incentive function is a mapping of the follower’s decision space to the leader’s decision space, i.e., γ: $\Omega_{F}\to \Omega_{L}$, so the leader’s decision now follows directly from the follower’s decision. Note that the Stackelberg game is a special case of the inverse Stackelberg game where the incentive function maps to a constant, that is, the case in which γ: $\Omega_{F}\to \left\{\omega_{L}\right\}$. In addition, the incentive function approach allows the leader to first determine a particular desired point $\left(\omega_{L}^{d},\omega_{F}^{d}\right)$ that he intends to achieve. A natural choice for this $\left(\omega_{L}^{d},\omega_{F}^{d}\right)$ would be a global optimum of the leader $\left(\omega_{L}^{*},\omega_{F}^{*}\right)\in \arg\max_{\omega_{L}\in \Omega_{L},\;\omega_{F}\in \Omega_{F}}J_{L}\left(\omega_{L},\omega_{F}\right)$. Given the desired point, the problem can be formulated as follows:

(1)Find:γ∈Γ,(2)s.t.(ωLd,ωFd)∈argmaxωF∈ΩFJF(γ(ωF),ωF),

where Γ={γ: $\Omega_{F}\to \Omega_{L}\}$ is the admissible set of incentive functions γ from which the leader can choose. Even this static inverse Stackelberg problem with a single leader and a single follower is hard to solve analytically because of the function composition, the fact that there could be more than one best answer and the fact that the follower response might not be unique. We can say for sure that this inverse Stackelberg game is at least strongly NP-hard, as it can be posted as the following program:

(3)(ωLd,ωFd)∈argmaxωL∈ΩL,ωF∈ΩFJL(γ(ωF),ωF),(4)s.t.ωFd∈argmaxωF∈ΩFJF(γ(ωF),ωF).

Taking $\omega_{L}$ as a free variable for $\gamma (\omega_{F})$, this is equivalent to a bilevel optimization problem formulation shown in Equation (5) next, which is proven to be strongly NP-hard even when the objective functions are linear [11].

(5) $$\begin{array}{c} \begin{array}{cc} \max_{x_{U}\in X_{U},\;x_{L}\in X_{L}} & F(x_{U},x_{L}),\\ s.t.\; & x_{L}\in \arg\max\left\{f\left(x_{U},x_{L}\right)|g_{i}\left(x_{U},x_{L}\right)\leq 0,i\in I\right\},\\ \; & G_{j}\left(x_{U},x_{L}\right)\leq 0,j\in J, \end{array} \end{array}$$

where $X_{U}$, $X_{L}$ denote the decision space of the upper and lower levels, respectively, *F*: $X_{U}\times X_{L}\to R$ is the upper-level objective function, *f*: $X_{U}\times X_{L}\to R$ is the lower-level objective function, and the functions $g_{i}$: $X_{U}\times X_{L}\to R$, $G_{i}$: $X_{U}\times X_{L}\to R$ represents the lower-level and upper-level constraints respectively, for i∈I⊆N, j∈J⊆N. This nested two-level optimization task structure requires that only optimal solutions of the lower-level task are acceptable as feasible solutions of the upper-level task.

### 2.2. The Single-Stage Principal–Agent Problem

In practice, the leader and follower agents base their decisions on a set of information that is readily accessible to them. Incentive design (ID), also known as contract theory, investigates inverse Stackelberg games under a variety of information models available to the leader and the follower agent and the information asymmetries between them. In this domain, the inverse Stackelberg game is called the principal–agent problem, with the leader as the principal and the follower as the agent. The way this information set is conceived and theoretically described is a major factor in how the fields of economics, control theory, and machine learning approach the principal–agent problem.

In this research, we focus on the single-stage principal–agent problem (SS-PAP), which is a hierarchical inverse Stackelberg game with a specific information model played between a single principal (leader) and a single follower. This single-stage game problem is as follows: First, the principal decides or selects an incentive function γ from a predetermined set of functions Γ={γ: $\Omega_{F}\to \Omega_{L}\}$, and announces γ to the follower, without knowledge of the follower’s utility. Then, with knowledge of this incentive function γ, the follower agent selects an action ${\omega_{F}}^{*}\in \Omega_{F}$ that maximizes his utility $J_{F}\left(\gamma \left(\omega_{F}\right),\omega_{F}\right)$. We do not impose restrictive assumptions on the functional form of utilities. Regarding the information model, we assume that both the principal and the follower agent possess private information, i.e., the utility function of the follower is unknown to the principal and vice versa. The outcome or result in this single-stage principal–agent problem are the utilities $J_{F}\left(\omega_{F}^{*}\right),\omega_{F}^{*})$ and $J_{L}\left(\omega_{F}^{*}\right),\omega_{F}^{*})$ after the principal decides the incentive function γ. Our focus is on the principal’s perspective of the problem, where the principal wants to select the incentive function γ that maximizes its utility $J_{L}$.

We assume that the principal solves this problem as a sequential optimization problem with finite horizon *T*, using previously observed historical data, given as *N* observations $D^{-1}=\{(\gamma^{i},\omega_{F}^{i},\omega_{L}^{i},J_{L}^{i}{)\}}_{i=1}^{N}$ of the system. That is, using $D^{-1}=\{(\gamma^{i},\omega_{F}^{i},\omega_{L}^{i},J_{L}^{i}{)\}}_{i=1}^{N}$, to estimate $E[J_{L}^{t}]$ given that an incentive function $\gamma^{t}\in \Gamma$ is applied, for rounds *T*, the principal decides on the best $\gamma^{*}\in \Gamma$ to implement in the SS-PAP. In other words, the principal (as the decision-maker), at each stage t∈[T], is responsible for the development of a policy $\pi^{t}$: $D^{t-1}\to \gamma^{t}$, where $D^{t-1}=\left\{\left(\gamma^{1},E\left[J_{L}^{1}\right]\right),\ldots ,\left(\gamma^{t-1},E\left[J_{L}^{t-1}\right]\right)\right\}$; and when the accumulated history data set $D^{T}$ is complete, the principal decides the incentive function $\gamma^{+}$ such that

(6) $$\gamma^{+}\in \underset{\left(\gamma^{t},\;E\left[J_{L}^{t}\right]\right)\in D^{T}}{\arg\max}\;\left\{E\left[J_{L}^{1}\right],\ldots ,E\left[J_{L}^{T}\right]\right\}.$$

Therefore, we deal with a sequential optimization problem, and we take a Bayesian approach to the optimization of the functional objective of the principal. Appendix C provides details about Bayesian optimization (BO), which specializes in sequentially optimizing objectives with the above-mentioned characteristics, relying on a statistical model of the objective function whose beliefs guide the algorithm in making the most fruitful decisions.

### 2.3. Causal Graphical Models and Causal Inference

This research proposes solving the single-stage principal agent problem (SS-PAP) as defined in the previous subsection by incorporating causal reasoning. In this section, we provide the essential concepts of causal inference required to understand the Causal Incentive Design (CID) framework proposed in Section 3 to solve SS-PAP.

A causal graphical model (CGM) consists of a directed acyclic graph (DAG) G, which is called the causal structure, together with a four-tuple 〈U,V,F,p(U)〉 where

-
U is a set ${\left\{U_{i}\right\}}_{i\in \left[p\right]}$ of exogenous (unobserved) variables, which are determined by factors external to the model;
-
V is a set ${\left\{V_{i}\right\}}_{i\in \left[p\right]}$, with p∈N, of endogenous (observed) variables.
-
$F=\{f_{V_{1}},\ldots ,f_{V_{p}}\}$ is a set of functions known as structural equations, such that $V_{i}=f_{V_{i}}\left(\mathrm{pa}\left(v_{i}\right),u_{i}\right)$, for each $V_{i}\in V$, with $\mathrm{pa}(V_{i})\subset V$ denoting the parents of $V_{i}$;
-
p(U) is a set of probability distributions ${\left\{p\left(U_{i}\right)\right\}}_{i\in \left[p\right]}$ for each $U_{i}$, where $U_{i}$ is a random disturbance distributed according to $p(U_{i})$, independently of all other $U_{j}$ with i≠j.

The set of vertices in the causal structure G corresponds to a set of random variables $V=\{V_{1},\ldots ,V_{p}\}$ and each edge of the set of edges E represents a direct functional relationship between the corresponding variables, expressed by saying that $V_{i}$ is the direct cause of $V_{j}$ for an edge $(V_{i},V_{j})\in E$. So, the graph G encodes the causal relationship between the variables in V. We assume that the system V is causally sufficient, and therefore there are no variables in V that are common direct causes of at least two observed variables that are unmeasured. Let G be the causal structure of a CGM, i.e., a directed acyclic graph (DAG) with variables V. The statistical model associated to G consists of all distributions that factorize as

(7) $$p\left(V\right)\;=\;\prod_{V\in V}p\;\left(V|{\mathrm{pa}}_{G}\left(V\right)\right),$$

where ${\mathrm{pa}}_{G}\left(V\right)$ denotes the parents of *V* in G.

The notation do(X=x) indicates an idealized experiment or intervention in which the values of *X* are set to *x*, for some X∈X⊆V. Hard interventions is the term used to describe this category of interventions. A fundamental problem in Causal Inference is to identify p(y|do(X=x)) from observational data. In practice, experimental data are not always accessible due to the fact that randomized controlled experiments might be impractical, costly, or even unethical. Within the causal system V, we distinguish three different types of variables: no manipulative variables C, treatment variables X that can be set to specific values, i.e., intervene them, and a target variable *Y* that represents the outcome of interest. For a fully observed causal system V with a causal structure DAG G, the post-intervention distribution can be identified through the *g-formula*, also known as the truncated formula. We follow the standard g-formula identification [26]:

(8) $$p\left(V\setminus X|do\left(X=x\right)\right)=\prod_{V\in V\setminus X}p\;\left(V|\mathrm{pa}(V)\right){|}_{X=x}.$$

So, a CGM induces joint observational and interventional probability distributions given in Equations (7) and (18). Pearl (see [26]) formalized the concept of causal effect of *X* on *Y* after manipulating *X*, that is, doing X=x for some x∈R(X), where R(·) denotes the range of a random variable, as the expected value of the distribution p(Y|do(X=x)), i.e., the distribution of *Y* after operation do(X=x), denoted as $E_{p(Y|do(X=x))}\left[Y\right]$ or $E\left[Y|do(X=x)\right]$. Like any ordinary probability distribution, $p\left(\cdot |do(X=x)\right)$ obeys the same rules of conditioning and marginalization. So, we can specify expectations in the conventional manner, as shown in Equation (9).

(9) $$E\left[Y|do(X=x)\right]=\int_{R(Y)}y\;p\left(y|do(X=x)\right)dy$$

The traditional hard interventions discussed above are unconditional actions that simply force a variable *X* to adopt a designated value *x*. Functional interventions refer to a broader category of interventions in which a variable *X* is manipulated to respond in a predetermined manner to a set of other observable variables Z⊆Pa(X) in the causal system through a functional relationship X=g(z). Specifically, a functional intervention can be considered as a substitution of the structural equation $f_{X}\in F$, of the intervened variable *X*, with a deterministic function of some parents in the CGM. In other words, by a functional intervention, we substitute the structural equation $X=f_{X}\left(\mathrm{pa}\left(x\right),u_{x}\right)$ with a function X=g(z) for some Z⊆Pa(X) in the causal model. It is important to note that hard interventions are the specific instance of functional interventions in which the structural equation $f_{X}$ is substituted by a constant function *g*.

Pearl first explored functional interventions under the name of conditional actions in [2]. More recently, Correa and Bareinboim in [3] introduced a new set of inference rules that extends the do-calculus for hard interventions in [2] to derive claims about functional interventions. Following these, let p(y∣do(X=g(z))) stand for the post-intervention distribution of *Y* established under the policy X=g(z). To compute the post-intervention distribution p(y∣do(X=g(z))) for the functional intervention do(X=g(z)), we condition on Z and write

(10) $$\begin{array}{c} \begin{array}{cc} \; & p(y|do(X=g(z)))\\ \; & =\int_{R(Z)}p\left(y|do\left(X=g\left(z\right)\right),z\right)p\left(z|do\left(X=g\left(z\right)\right)\right)\\ \; & =\int_{R(Z)}p\left(y|do\left(X=g\left(z\right)\right),z\right)p\left(z\right)\\ \; & =E[p(y|do(X=g(z)),z)]\;\mathrm{with}\;x=g(z), \end{array} \end{array}$$

where the equality p(z∣do(X=g(z)))=p(z) comes from the fact that Z cannot be a descendant of *X*, and therefore any intervention in *X* has no effect on the distribution of Z. Thus, the causal effect of a functional intervention X=g(Z) can be computed from the expression p(y∣do(X=x),z) by substituting *x* for g(z) and taking the expectation over Z.

## 3. Causal Incentive Design for Single-Stage Canonical PAPs

In this section, we develop the proposed Causal Incentive Design (CID) framework by showing how causal inference can be incorporated into the principal–agent problem described in Section 2.2 to solve it. First in Section 3.1, we propose the general additive-noise Gaussian CGM $M_{L_{1}F_{1}}$ that represents the dynamics of the single-stage canonical principal–agent problem $PAP_{L_{1}F_{1}}$ and describe its key semantic aspects and underlying assumptions. This $PAP_{L_{1}F_{1}}$ is the canonical version in which the principal–agent problem is analyzed with a single principal controlling a unique decision variable $\omega_{L}\in \Omega_{L}$ and a single follower agent with a unique decision variable $\omega_{F}\in \Omega_{F}$. The core concepts of CID can be elucidated through the examination of the canonical principal–agent problem $PAP_{L1F1}$ and the CGM $M_{L_{1}F_{1}}$ that represents it.

The CID for the single-stage PAP is developed in three parts: (i) specification of the additive-noise CGM for the single-stage PAP and definition of the policy-value target $V\left(\gamma \right)=E[J_{L}|do\left(\Gamma =\gamma \right)]$ (Section 3.1); (ii) derivation of a semi-parametric identification for V(γ) via the g-formula, demonstrating its decomposition into an inner Gaussian smoothing and an outer expectation over the follower’s action (Section 3.2); and (iii) presentation of a two-layer estimator (Section 3.3), which first computes the inner Gaussian expectation (Section 3.3.3), and then approximates the outer expectation using policy-local kernel reweighting with accompanying diagnostics (Section 3.3.4).

### 3.1. A Causal Graphical Model for the Single-Stage Canonical Principal–Agent Problem

We consider the single-stage canonical principal–agent problem $PAP_{L_{1}F_{1}}$ as a causal system with endogenous observable random variables $V=\{\Gamma ,\Omega_{L},\Omega_{F},J_{F},J_{L}\}$, where Γ is the admissible set of incentive functions with range $R_{\Gamma }=\Gamma$. The variables $\Omega_{L},\Omega_{F}$ represent the decision variables of the principal and the follower, respectively, with ranges $R_{\Omega_{L}}=\Omega_{L}$ and $R_{\Omega_{F}}=\Omega_{F}$, where $\Omega_{L}$, $\Omega_{F}$ are the decision spaces of the principal and the follower, respectively. The variables $J_{F},J_{L}$ represent the values of the utility functions of the follower and the principal, respectively.

**Definition** **1.** *The CGM $M_{L_{1}F_{1}}$ for $PAP_{L_{1}F_{1}}$ has endogenous variables set V, exogenous variables set U, and structural equations set F given as follows:*

$$V=\left\{\Gamma ,\Omega_{L},\Omega_{F},J_{F},J_{L}\right\},\;U=\left\{U_{\Omega_{L}},U_{\Omega_{F}},U_{J_{F}},U_{J_{L}}\right\},\;F=\left\{f_{\Gamma },f_{\Omega_{L}},f_{\Omega_{F}},f_{J_{F}},f_{J_{L}}\right\},\;\mathrm{with}\;$$

(11)Γ:= γ(12)ΩF:= fΩF(γ,ϵΩF)=BR(γ)+ϵΩF(13)ΩL:= fΩL(γ,ΩF,ϵΩL)=γ(ΩF)+ϵΩL(14)JF:= fJF(ΩL,ΩF,ϵJF)=JF(ΩL,ΩF)+ϵJF(15)JL:= fJL(ΩL,ΩF,ϵJL)=JL(ΩL,ΩF)+ϵJL

*where $\epsilon_{\Omega_{F}}\in R_{U_{\Omega_{F}}}$, $\epsilon_{\Omega_{L}}\in R_{U_{\Omega_{L}}}$, $\epsilon_{\Omega_{J_{F}}}\in R_{U_{J_{F}}}$, $\epsilon_{\Omega_{J_{L}}}\in R_{U_{J_{L}}}$; with $p\left(U_{i}\right)=N\left(0,\sigma_{i}^{2}\right)$ for all $U_{i}\in U$ and p(U) is jointly independent. The function $\gamma :R_{\Omega_{F}}\to R_{\Omega_{L}}\in \Gamma$ is an incentive function, decided by the principal from the function space *Γ*. The functional BR(γ) is the best response functional for the follower agent given γ. The functions $J_{F}\left(\omega_{L},\omega_{F}\right)$, $J_{L}\left(\omega_{L},\omega_{F}\right)$ are the utility functions of the follower and the leader, respectively, in the $PAP_{L1F1}$.*

#### Assumptions Underlying the CGMs for Single-Stage Canonical PAPs

The CGM $M_{L_{1}F_{1}}$ is an additive-noise Gaussian, Structural Causal Model (SCM); not inherently linear, as we do not assume that the structural equations $f_{V_{i}}\left(\mathrm{pa}\left(v_{i}\right),u_{i}\right)$, for each $V_{i}\in V$, hold a specific functional form. We assume that the set of endogenous variables $V=\{\Gamma ,\Omega_{L},\Omega_{F},J_{F},J_{L}\}$ in the CGM—namely, the incentives variable Γ, the principal’s decision variable $\Omega_{L}$, the follower’s decision variable $\Omega_{F}$, and their respective utilities $J_{L}$ and $J_{F}$—is causally sufficient. That is, there are no latent common causes outside this set that jointly influence any two of these variables.

The causal structure $G_{L_{1}F_{1}}$ of $M_{L_{1}F_{1}}$, has directed edges from Γ to $\left\{\Omega_{L},\Omega_{F}\right\}$, from $\Omega_{L}$ to $\left\{J_{F},J_{L}\right\}$, and from $\Omega_{F}$ to $\left\{\Omega_{L},J_{F},J_{L}\right\}$. The variable Γ does not depend on an exogenous random variable or any other variable in the causal system; it is a deterministic root variable whose value is decided and fixed by the principal choice of γ∈Γ. Thus, γ acts as a fixed input to the causal system, selected by the principal agent.

The best response functional BR(γ) in the structural Equation (13), is the mapping from the principal’s chosen incentive function γ to the action $\omega_{F}^{*}$ that maximizes the follower’s utility. That is,

(16) $$BR\left(\gamma \right)=\omega_{F}^{*}\in \underset{\omega_{F}\in \Omega_{F}}{\arg\max}\;E\left[J_{F}\left(\gamma \left(\omega_{F}\right)+\epsilon_{\Omega_{L}},\omega_{F}\right)\right].$$

This formulation reveals how the follower’s decision responds to the incentive function chosen by the principal. The structural Equation (14), $\Omega_{L}:=\;f_{\Omega_{L}}\left(\gamma ,\Omega_{F},\epsilon_{\Omega_{L}}\right)=\gamma \left(\Omega_{F}\right)+\epsilon_{\Omega_{L}}$, is central to represent the inverse Stackelberg game semantics. It expresses that after the principal proposes the incentive function γ, the principal’s action $\Omega_{L}$ is given as a function $\gamma (\omega_{F})$ of the follower’s action $\omega_{F}$. This composition in the directed acyclic triangle $\Gamma \to \Omega_{F}$, $\Omega_{F}\to \Omega_{L}$, $\Gamma \to \Omega_{L}$, with the structural Equations (13) and (14), and it highlights how the principal’s concrete action $\Omega_{L}$ responds to the follower’s action, influenced by the incentive function γ selected by the principal. The utility variables $J_{L}$ and $J_{F}$ are defined as functions of $\left(\Omega_{L},\Omega_{F}\right)$, consistent with conventional formulations of principal–agent problems.

The generative causal model $M_{L_{1}F_{1}}$ must be examined from two perspectives: the principal’s and the follower’s. From the principal’s view, the follower’s utility $J_{F}$ is an unobservable or hidden variable. So, for the principal, the set of hidden variables is $H_{L}=\left\{J_{F}\right\}$, while the set of observable variables is $O_{L}=\left\{\Gamma ,\Omega_{L},\Omega_{F},J_{L}\right\}$, with $H_{L}\cup O_{L}=V$. On the other hand, from the follower’s view, $H_{F}=\left\{J_{L}\right\}$ and $O_{F}=\left\{\Gamma ,\Omega_{L},\Omega_{F},J_{F}\right\}$, with $H_{F}\cup O_{F}=V$. These separated perspectives are just marginalizations of the same generative SCM $M_{L_{1}F_{1}}$, but maintain consistency with the information model in $PAP_{L_{1},F_{1}}$.

It is also important to establish that, with respect to the information model in $PAP_{L_{1},F_{1}}$, the principal views the follower mechanism $f_{\Omega_{F}}\left(\gamma ,U_{\Omega_{F}}\right)$ as a black box. In other words, the principal does not know the function BR(γ) that the follower uses to decide $\omega_{F}$, but only observes the decision $\omega_{F}$. This work centers on the principal’s perspective, and for the remainder, we will concentrate exclusively on this view of the causal model $M_{L_{1}F_{1}}$, assuming $V=O_{L}\cup H_{L}$ and treating $f_{\Omega_{F}}\left(\gamma ,U_{\Omega_{F}}\right)$ as a black box.

### 3.2. A Non-Parametric Identification for the Causal Inference Target

In the Causal Incentive Design (CID) framework, with respect to the single-stage $PAP_{L_{1}F_{1}}$, the principal’s objective is to determine an incentive function $\gamma^{*}$ such that its utility $J_{L}$ is maximized, utilizing prior observations of the system in question. To accomplish this, the principal should first be able to estimate the expected value of their utility $J_{L}$, in the particular scenario where the incentive function γ is applied, ideally utilizing only observational data.

In the context of causal inference, this requires determining whether the expected value of the utility of the principal $E[J_{L}]$ in the post-intervention distribution $p\left(J_{L}|do\left(\Gamma =\gamma \right)\right)$ is identifiable. In other words, determine whether

(17) $$E_{p\left(J_{L}|do\left(\Gamma =\gamma \right)\right)}\left[J_{L}\right]\;\;\mathrm{or}\;\mathrm{equivalently},\;\;E\left[J_{L}|do\left(\Gamma =\gamma \right)\right],$$

can be estimated using exclusively observational data from variables $\left(\Gamma ,\Omega_{L},\Omega_{F},J_{L}\right)$, i.e., a data set $D={\left\{\gamma^{i},\omega_{F}^{i},\omega_{L}^{i},j_{L}^{i}\right\}}_{i=1}^{N}$ of N∈N observations of the system. We also employ the simpler notation $E^{\gamma }\left[J_{L}\right]$ to denote $E\left[J_{L}|do\left(\Gamma =\gamma \right)\right]$. Recall that the variable $J_{F}$ is a hidden variable from the principal’s perspective; so the principal has no knowledge or information about this variable.

#### Identification via the g-Formula

We first use the g-formula over the principal’s perspective to construct a non–parametric identification for the causal inference target $E\left[J_{L}|do\left(\Gamma =\gamma \right)\right]$, which is then adequate to become a semi-parametric target estimand by considering the Gaussian additive independent errors on the CGM $M_{L_{1}F_{1}}$. The g-formula over the principal’s perspective for the intervention do(Γ=γ) is given by

(18) $$p\left(O_{L}\setminus \Gamma |do\left(\Gamma =\gamma \right)\right)=\prod_{O_{L}\in O_{L}\setminus \Gamma }p\;(O_{L}|\mathrm{pa}\left(O_{L}\right)){|}_{\Gamma =\gamma }.$$

That is, we obtain the post-intervention distribution $p\left(O_{L}\setminus \Gamma |do\left(\Gamma =\gamma \right)\right)=p\left(\omega_{F},\omega_{L},j_{L}|do\left(\Gamma =\gamma \right)\right)$, removing all factors from the pre-intervention distribution $p(O_{L})$ corresponding to the intervened variable and substituting Γ=γ into the remaining factors. So, the post-interventional distribution $p\left(\omega_{F},\omega_{L},j_{L}|do\left(\Gamma =\gamma \right)\right)$ is factorized as shown in Equation (19). Then, we can estimate the target post-intervention distribution $p(j_{L}|do\left(\Gamma =\gamma \right))$ by marginalizing the variables $\omega_{F}$ and $\omega_{L}$ on the factorization for $p\left(\omega_{F},\omega_{L},j_{L}|do\left(\Gamma =\gamma \right)\right)$, as shown in Equation (20).

(19) $$p\left(\omega_{F},\omega_{L},j_{L}|do\left(\Gamma =\gamma \right)\right)=p\left(\omega_{F}|\Gamma =\gamma \right)\;p\left(\omega_{L}|\omega_{F},\Gamma =\gamma \right)\;p\left(j_{L}|\omega_{L},\omega_{F},\Gamma =\gamma \right)\;$$

(20) $$p\left(j_{L}|do\left(\Gamma =\gamma \right)\right)=\;\int \int \;p\left(j_{L}|\omega_{L},\omega_{F},\Gamma =\gamma \right)\;p\left(\omega_{L}|\omega_{F},\Gamma =\gamma \right)\;p\left(\omega_{F}|\Gamma =\gamma \right)\;d\omega_{L}\;d\omega_{F}.$$

The target functional value $V\left(\gamma \right)=E\left[J_{L}|do\left(\Gamma =\gamma \right)\right]$ under the incentive function (policy) γ is the expectation of $J_{L}$ in the post-intervention world, that is,

(21) $$\begin{array}{cc} V(\gamma ) & =E^{\gamma }\left[J_{L}\right]=\int j_{L}\;p\left(j_{L}|do\left(\Gamma =\gamma \right)\right)\;dj_{L}. \end{array}$$

Therefore, substituting the factorization form (Equation (20)) of the post-intervention distribution $p(j_{L}|do\left(\Gamma =\gamma \right))$, we obtain the following:

(22)V(γ)=∫j∫∫p(j∣ωL,ωF,Γ=γ)p(ωL∣ωF,Γ=γ)p(ωF∣Γ=γ)dωLdωFdj.(23)=∫∫∫jp(j∣ωL,ωF,Γ=γ)djp(ωL∣ωF,Γ=γ)p(ωF∣Γ=γ)dωLdωF,

where the exchange of integrals is allowed by the Tonelli/Fubini Theorem. Observe that the inner integral is the following conditional mean:

(24) $$\begin{array}{cc} \int j\;p(j|\omega_{L},\omega_{F},\Gamma =\gamma )\;dj & =E[Y|\Omega_{L}=\omega_{L},\Omega_{F}=\omega_{F},\Gamma =\gamma ] \end{array}$$

Let us denote $\mu_{L}\left(\omega_{L},\omega_{F},\gamma \right):=\;E\left[Y|\Omega_{L}=\omega_{L},\Omega_{F}=\omega_{F},\Gamma =\gamma \right]$. Thus, we have

(25)V(γ)=∫∫μL(ωL,ωF,γ)p(ωL∣ωF,Γ=γ)p(ωF∣Γ=γ)dωLdωF(26)=∫∫μL(ωL,ωF,γ)p(ωL∣ωF,Γ=γ)dωLp(ωF∣Γ=γ)dωF.

From structural Equation (14), $\Omega_{L}:=\;f_{\Omega_{L}}\left(\gamma ,\Omega_{F},\epsilon_{\Omega_{L}}\right)=\gamma \left(\Omega_{F}\right)+\epsilon_{\Omega_{L}}$, if $U_{\Omega_{L}}$ has density $f_{U_{\Omega_{L}}}$, then for a fixed $\omega_{F}$, we have $p\left(\omega_{L}|\omega_{F},\Gamma =\gamma \right)=f_{U_{L}}\left(\omega_{L}-\gamma \left(\omega_{F}\right)\right)$. Hence, the inner integral of Equation (26) becomes

(27) $$\int \mu_{L}\left(\omega_{L},\omega_{F},\gamma \right)\;f_{U_{L}}\left(\omega_{L}-\gamma \left(\omega_{F}\right)\right)\;d\omega_{L}.$$

Which, by the change of variable $r=\omega_{L}-\gamma \left(\omega_{F}\right)$, $d\omega_{L}=d\left(r+\gamma (\omega_{F})\right)$, becomes

(28) $$\int \mu_{L}\left(\gamma \left(\omega_{F}\right)+t,\omega_{F},\gamma \right)\;f_{U_{\Omega_{L}}}\left(r\right)\;d\left(r+\gamma (\omega_{F})\right)=E_{U_{\Omega_{L}}}\left[\mu_{L}\left(\gamma \left(\omega_{F}\right)+U_{\Omega_{L}},\omega_{F},\gamma \right)\right],$$

since $r=\omega_{L}-\gamma \left(\omega_{F}\right)=\epsilon_{\Omega_{L}}∼p\left(U_{\Omega_{L}}\right)$, as $\Omega_{L}=\gamma \left(\omega_{F}\right)+U_{\Omega_{L}}$ with $U_{\Omega_{L}}⊥\Omega_{F}$. Therefore, Equation (26) can be expressed as

(29) $$\begin{array}{cc} V(\gamma ) & =\int \left\{\int \mu_{L}\left(\omega_{L},\omega_{F},\gamma \right)\;p\left(\omega_{L}|\omega_{F},\Gamma =\gamma \right)\;d\omega_{L}\right\}\;p\left(\omega_{F}|\Gamma =\gamma \right)\;d\omega_{F}\\ \; & =\int \left\{E_{U_{\Omega_{L}}}\left[\mu_{L}\left(\gamma \left(\omega_{F}\right)+U_{\Omega_{L}},\omega_{F},\gamma \right)\right]\right\}\;p\left(\omega_{F}|\Gamma =\gamma \right)\;d\omega_{F}. \end{array}$$

The outer integral of Equation (29) corresponds to averaging over the follower’s action distribution $p(\Omega_{F}|\Gamma =\gamma )$ induced by the policy γ. Recall that from the principal’s perspective, the follower mechanism $\Omega_{F}=f_{\Omega_{F}}\left(\gamma ,U_{\Omega_{F}}\right)$ is unknown and is treated as a black-box mechanism.

### 3.3. Estimations on the Semi-Parametric Identification Formula

We now demonstrate how to estimate V(γ); first through a working example in the context of the credit market, and subsequently, we describe how the estimation can be conducted in general under additive, independent, mean-zero Gaussian noise, without committing to a particular parametric form for the structural functions. In this section, for simplicity and clarity on the estimation task, we rename two of the causal system variables. Here, we use $M:=\Omega_{F}$ to denote the follower’s action and refer to it as the mediator, because it acts as a *mediator* on the causal path from a fixed incentive policy γ∈Γ to the principal’s utility $J_{L}$. We refer here to the principal’s utility as the *outcome* of the system, and we denote it here as $Y:=J_{L}$ for convenience.

Before going into the details for the estimation on the linear parametrized PAP example and the general case estimation; we provide an introduction of the general components to learn on the identified estimand:

(30)V(γ)=EY∣do(Γ=γ)(31)=∫{EULμL(γ(m)+UL,m,γ)︸Innerexpectationfortheoutcomemodel.}p(m∣Γ=γ)︸Black-boxmediatorconditional.dm,

where $\mu_{L}\left(\omega_{L},m,\gamma \right)=E\left[Y|\Omega_{L}=\omega_{L},\;M=m,\;\Gamma =\gamma \right]$, and $\Omega_{L}=\gamma \left(M\right)+U_{L}$ holds. The two main components are inner expectation for the outcome model and the outer black-box mediator conditional, as shown in Equation (31).

For the first, we unpack the nested structure by first estimating the action mechanism $\Omega_{L}=f_{\Omega_{L}}\left(\gamma ,M,U_{L}\right)={\hat{f}}_{L}\left(\gamma ,M\right)+{\hat{\sigma }}_{L}\left(\gamma ,M\right)\;U_{L}$ by learning ${\hat{f}}_{L}\left(\gamma ,M\right)$ and ${\hat{\sigma }}_{L}\left(\gamma ,M\right)$ from the data; i.e., regress $\Omega_{L}$ on (M,Γ) for the mean ${\hat{f}}_{L}$ and regress squared residuals on (M,Γ) to obtain ${\hat{\sigma }}_{L}^{2}$. With these, we learn the conditional outcome mean $\mu_{L}\left(\omega_{L},m,\gamma \right)=E\left[Y|\Omega_{L}=\omega_{L},\;M=m,\;\Gamma =\gamma \right]$ as regression problem with features $\left(\Omega_{L},M,\Gamma \right)$ and outcome *Y*. Then, the inner expectation for the outcome model (see Equation (31)) may be computed by Gauss–Hermite quadrature or Monte Carlo using common random numbers across *m* to reduce variance.

The second main component is the post-intervention mediator law (what the follower does under the policy γ); the black-box mediator conditional p(m∣Γ=γ). We approximate p(m∣Γ=γ) near the target policy by a policy-local reweighting of the observation, selecting a policy-space metric $d_{G}$ and kernel *K* with bandwidth *h*. Thus, the outer expectation over the mediator law under a fixed γ is computed by policy-local kernel weights. Therefore, in general, the inner layer (first component) is a Gaussian smoothing of the outcome regression around the mean principal’s action (in linear models, the variance drops out and we recover the closed form; in general, we keep a small quadrature); and the outer layer (second component) is a weighted average over observed mediators. The kernel with bandwidth provides a principled way to compute those weights with global regularization as we show in the following sections.

Since empirical performance is determined by domain-specific design choices, such as policy parameterization, kernel selection on policy space, bandwidth *h*, and support criteria, we defer numerical examples and simulations to future work that will focus on applied instantiations of the present framework. We concentrate on formal properties that hold independent of the chosen application.

#### 3.3.1. The Estimation of a Linear Parametrized Instance

Consider a financial institution that is required to establish a transparent pricing plan before evaluating a potential borrower’s response. The bank (the principal) announces an affine policy, which includes a fixed fee and an interest “slope” that is dependent on the size of the loan. The bank, thereafter, observes how much the business (the follower) actually borrows. This is an instance of a canonical $PAP_{L_{1}F_{1}}$, which we can parameterize as the following linear Gaussian Structural Causal Models (SCM).

(32)Γ:= γ(α,β)(m)=αm+β,(33)M:= μM(α,β)+UM,UM∼N(0,σM2),(34)ΩL:= αM+β+UΩL,UΩL∼N(0,σL2),(35)Y:= b+rΩL+qM+UY,UY∼N(0,σY2),

with $U_{M},U_{\Omega_{L}},U_{Y}$ mutually independent and independent of Γ. We model the bank’s pricing rule as an affine policy $\gamma_{\left(\alpha ,\beta \right)}\left(m\right)=\alpha m+\beta$, with α≥0, β≥0; so, we have an affine pricing as the incentive functions family. As we have assumed from the principal’s perspective, the follower’s mechanism and utility remain hidden from the principal. So, the function $\mu_{M}\left(\alpha ,\beta \right)$ is an unknown function for the principal. In this context, we can see $\Omega_{F}$ as the realized cash inflow from the borrower, i.e., the bank collections follow the mechanism $\Omega_{L}=\gamma \left(M\right)+U_{L}=\alpha M+\beta +U_{L}$; the interest or the cash inflow *M* plus the fee actually received at the single stage, possibly noisy. The principal utility is consider linear given as $Y=b+r\;\Omega_{L}+q\;M+U_{Y}$, where *b* represents the baseline overheads, *r* is the realization factor that converts collections into value (often r≈1 if a dollar collected is a dollar of revenue before costs; r < 1 if haircuts rules apply), and *q* is the marginal value of the loan size *M*. It is typically negative if it aggregates funding cost, expected credit loss, and capital operating costs per unit of balance.

$$\begin{array}{cc} \underset{\mathrm{Profit}}{\underset{︸}{J_{L}}} & =\underset{\mathrm{Collections}}{\underset{︸}{r\Omega_{L}}}+\underset{\mathrm{Funding}\mathrm{and}\mathrm{risk}\mathrm{cost}\mathrm{per}\mathrm{unit}}{\underset{︸}{q\;M}}+\underset{\mathrm{Fixed}\mathrm{margin}}{\underset{︸}{b}}\\ \; & =\;r\;\left(\alpha M+\beta \right)+q\;M+b. \end{array}$$

In short, profit is given as the collections minus (negative *q*) the funding cost per size. This aligns with the canonical $PAP_{L_{1}F_{1}}$, where the utility of the principal depends on the decisions of the leader $\Omega_{L}$ and the follower $\Omega_{F}$.

Given this outcome model, the conditional mean $\mu_{L}\left(\omega_{L},m,\gamma \right)$ used inside the g-formula identification in Equation (29) is

$$\begin{array}{cc} \mu_{L}\left(\omega_{L},m,\gamma \right) & =E[Y|\Omega_{L}=\omega_{L},\;M=m,\;\Gamma =\gamma ]\\ \; & =b+r\;\omega_{L}+q\;m, \end{array}$$

since $E[U_{Y}|\Omega_{L},M,\Gamma ]=0$. Then, we reduce $E_{U_{\Omega_{L}}}\;\left[\mu_{L}\left(\gamma \left(m\right)+U_{\Omega_{L}},\;m,\;\gamma \right)\right]$, the inner expectation over the principal’s action noise $U_{\Omega_{L}}$ in the identification formula, using $\Omega_{L}=\gamma \left(m\right)+U_{\Omega_{L}}$ and $E[U_{\Omega_{L}}|M=m,\Gamma =\gamma ]=0$. If we fix *m*, the inner term becomes

(36) $$\begin{array}{cc} E_{U_{\Omega_{L}}}\;\left[\mu_{L}\left(\gamma \left(m\right)+U_{\Omega_{L}},\;m,\;\gamma \right)\right] & =E\;\left[\;b+r(\gamma \left(m\right)+U_{\Omega_{L}})+q\;m\;\right]\\ \; & =b+r\;\gamma (m)+q\;m. \end{array}$$

Because $\mu_{L}$ is linear on $\omega_{L}$ and $U_{\Omega_{L}}$ is mean-zero (conditional on *m*), the action-noise variance do not affect the mean; only the mean of $\Omega_{L}$ matters at this step. We reduce the outer integral by plugging the reduced inner term (36) into the outer integral in (29), and take the (outer) expectation with respect to the mediator law p(m∣Γ=γ):

$$\begin{array}{cc} V(\gamma ) & =\int \left(b+r\;\gamma (m)+q\;m\right)\;p\left(m|\Gamma =\gamma \right)\;dm\\ \; & =b\;+\;r\;E\;\left[\gamma (M)|\Gamma =\gamma \right]\;+\;q\;E\left[M|\Gamma =\gamma \right]. \end{array}$$

Now, substituting the affine policy $\gamma_{\left(\alpha ,\beta \right)}\left(m\right)=\alpha m+\beta$:

$$\begin{array}{cc} E\;\left[\gamma (M)|\Gamma =(\alpha ,\beta )\right] & =E[\alpha M+\beta |\Gamma =(\alpha ,\beta )]\\ \; & =\alpha \;\mu_{M}\left(\alpha ,\beta \right)+\beta , \end{array}$$

with $\mu_{M}\left(\alpha ,\beta \right):=E\left[M|\Gamma =\left(\alpha ,\beta \right)\right]$. Hence,

(37) $$\begin{array}{cc} V(\alpha ,\beta ) & =b+r\left(\alpha \;\mu_{M}\left(\alpha ,\beta \right)+\beta \right)+q\;\mu_{M}\left(\alpha ,\beta \right)\\ \; & =\;b\;+\;r\;\beta \;+\;\left(r\alpha +q\right)\;\mu_{M}\left(\alpha ,\beta \right)\;. \end{array}$$

Therefore, the expression in (37) for V(α,β) is the estimand we want to approximate from the data, given as *N* i.i.d. observations $\{(\alpha_{i},\beta_{i},M_{i},\Omega_{L,i},Y_{i}{)\}}_{i=1}^{N}$. The formula in (37) depends on (i) the outcome coefficients (b,r,q), and (ii) the black-box mean of the mediator under policy $\mu_{M}\left(\alpha ,\beta \right)=E\left[M|\Gamma =\left(\alpha ,\beta \right)\right]$. We estimate them separately in two steps and subsequently input them into the closed form. In a first step, we estimate $\left(\hat{b},\hat{r},\hat{q}\right)$ from a regression of *Y* on $\left(\Omega_{L},M\right)$; and in a second step, the estimation of ${\hat{\mu }}_{M}\left(\alpha ,\beta \right)$ is performed through the regression of *M* with respect to the variables (α,β). The closed form estimator is then

(38) $$\hat{V}\left(\alpha ,\beta \right)=\hat{b}+\hat{r}\beta +\left(\hat{r}\alpha +\hat{q}\right){\hat{\mu }}_{M}\left(\alpha ,\beta \right).$$

A natural way to estimate $\left(\hat{b},\hat{r},\hat{q}\right)$ is by weighting observations according to their location in relation to (α,β). Then, a weighted least-squares (WLS) fit yields $\left(\hat{b},\hat{r},\hat{q}\right)$ that are tuned to the neighborhood of the target policy $\gamma_{\left(\alpha ,\beta \right)}$. That is, for a target incentive policy $\gamma_{\left(\alpha ,\beta \right)}\left(m\right)=\alpha m+\beta$, we fit a local linear outcome model $Y\approx b\;+\;r\;\Omega_{L}\;+\;q\;M\;+\;\varepsilon$, by weighting observations according to their proximity to $\gamma_{\left(\alpha ,\beta \right)}$ in the policy space.

Let $X_{i}=\left[\;1,\;\Omega_{L,i},\;M_{i}\;\right]$ and $y_{i}=Y_{i}$ for i∈[N], and arrange the data in the matrix $X\in R^{n\times 3}$, and the vector $y\in R^{n}$. A practical choice for a distance $d\;\left(\left(\alpha_{i},\beta_{i}\right),\left(\alpha ,\beta \right)\right)$ in the policy space is an isotropic distance defined as

$$\begin{array}{cc} \; & d\left(\left(\alpha_{i},\beta_{i}\right),\left(\alpha ,\beta \right)\right)=\sqrt{{\left({\tilde{\alpha }}_{i}-\tilde{\alpha }\right)}^{2}+{\left({\tilde{\beta }}_{i}-\tilde{\beta }\right)}^{2}}, \end{array}$$

with standardized coordinates $\tilde{\alpha }=\left(\alpha -\bar{\alpha }\right)/\hat{\mathrm{sd}}\left(\alpha \right)$ (and analogously for β).

Let K(·) be a radial kernel (Gaussian or Epanechnikov) and h>0 a scalar bandwidth. We then define the unnormalized weights as follows:

$${\tilde{w}}_{i}\left(h\right)\;=\;K\;\left(\frac{d(\left(\alpha_{i},\beta_{i}\right),\left(\alpha ,\beta \right))}{h}\right),\;w_{i}\left(h\right)\;=\;\frac{{\tilde{w}}_{i}\left(h\right)}{\sum_{j=1}^{n}{\tilde{w}}_{j}\left(h\right)},\;\sum_{i}w_{i}\left(h\right)=1,$$

and $W=\mathrm{diag}\left(w_{1}\left(h\right),\ldots ,w_{n}\left(h\right)\right)$. Then, the WLS estimator $\hat{\theta }={\left(\hat{b},\hat{r},\hat{q}\right)}^{⊤}$ is

$$\hat{\theta }\;=\;\left[\begin{array}{c} \hat{b}\\ \hat{r}\\ \hat{q} \end{array}\right]={\left(X^{⊤}WX\right)}^{-1}X^{⊤}Wy.$$

We estimate $\mu_{M}\left(\alpha ,\beta \right)=E\left[M|\Gamma =\left(\alpha ,\beta \right)\right]$ for a fixed policy (α,β), regressing the realized mediator *M* on the policy coordinates (α,β), using observed tuples ${\left\{\left(\alpha_{i},\beta_{i},M_{i}\right)\right\}}_{i=1}^{N}$. We proceed similarly in this regression with the idea of weighting observations according to their proximity to $\gamma_{\left(\alpha ,\beta \right)}$. Let $\theta_{i}=\left(\alpha_{i},\beta_{i}\right)$ and θ=(α,β) denote observed and target incentive policies. We use the same policy-space isotropic distance and unnormalized weights as in the previous regression.

We can approximate $\mu_{M}$ by a local plane around θ=(α,β), given as the linear combination $\theta_{0}+\theta_{1}\left(\alpha_{i}-\alpha \right)+\theta_{2}\left(\beta_{i}-\beta \right)$ of the variables $\left(\alpha_{i}-\alpha \right)$ and $\left(\beta_{i}-\beta \right)$. Thus, using the approximation $M_{i}\;\approx \;\theta_{0}+\theta_{1}\left(\alpha_{i}-\alpha \right)+\theta_{2}\left(\beta_{i}-\beta \right)$, the WLS problem is given by

(39) $$\min_{\theta_{0},\theta_{1},\theta_{2}}\;\sum_{i=1}^{N}w_{i}\left(h\right){\left[M_{i}-\left\{\theta_{0}+\theta_{1}\left(\alpha_{i}-\alpha \right)+\theta_{2}\left(\beta_{i}-\beta \right)\right\}\right]}^{2},$$

with the same kernel weights $w_{i}\left(h\right)$. Then, the estimate of the mean at the target is the intercept in this linear model. Therefore, $\hat{\theta_{0}}$ represents the estimated mean value of the plane at the specific target policy (α,β) and ${\hat{\mu }}_{M}\left(\alpha ,\beta \right)=\hat{\theta_{0}}$. In matrix form, let the local design row be $x_{i}=\left[\;1,\;\alpha_{i}-\alpha ,\;\beta_{i}-\beta \;\right]$, arranging in the matrix $X\in R^{n\times 3}$ the $x_{i}$, and $W=\mathrm{diag}\left(w_{1}\left(h\right),\ldots ,w_{n}\left(h\right)\right)$. A local linear WLS estimator for $\mu_{M}\left(\alpha ,\beta \right)=E\left[M|\Gamma =\left(\alpha ,\beta \right)\right]$ is

$$\hat{\theta }={\left(X^{⊤}WX\right)}^{-1}X^{⊤}W\;M,\;{\hat{\mu }}_{M}\left(\alpha ,\beta \right)={\hat{\theta }}_{0},\;\mathrm{with}\;\;M={\left(M_{1},\ldots ,M_{n}\right)}^{⊤}$$

#### 3.3.2. The Estimation in the General Gaussian Additive-Noise Case

We model the conditional distribution of the leader’s realized action $\Omega_{L}$ given the mediator *M* and policy γ as the following Gaussian:

(40) $$p\left(\Omega_{L}|M=m,\Gamma =\gamma \right)∼N\left({\hat{f}}_{L}\left(m,\gamma \right),\;\sigma_{L}^{2}\left(m,\gamma \right)\right)$$

We learn non-parametrically the mean map ${\hat{f}}_{L}$: M×A→R and the variance map $\sigma_{L}^{2}$: M×A→(0,∞) from data ${\left\{\left(M_{i},A_{i},\Omega_{L,i}\right)\right\}}_{i=1}^{N}$ using RKHS regression kernel, i.e., Kernel Ridge Regression (KRR) on (M,Γ) [4,27]. Therefore, to learn the mean function ${\hat{f}}_{L}$, we solve the KRR problem, where the goal is to find a function *f* that minimizes a regularized loss function.

(41) $${\hat{f}}_{L}\left(m,\gamma \right)\in \arg\min_{f\in H_{MA}}\;{\left(\omega_{Li}-f\left(m_{i},\gamma_{i}\right)\right)}^{2}+\lambda_{f}{∥f∥}_{H_{MA}}^{2}.$$

The first term measures how well the function fits the training data. The second term is a regularization penalty, which penalizes the complexity of the function as measured by its norm in the RKHS, using the regularization hyperparameter $\lambda_{f}$ that balances the trade-off. By the representer theorem, the solution of problem (41) can be expressed as a linear combination of kernel functions centered at the training data points $f\left(\cdot \right)=\sum_{i\in [N]}\alpha_{fi}k_{MA}\left(x_{i},\cdot \right)$. In matrix form, ${\hat{f}}_{L}\left(m,a\right)=k_{\left(m,a\right)}^{⊤}\alpha_{f}$, where $k_{\left(m,a\right)}={\left(k_{MA}\left(x_{1},\left(m,a\right)\right),\ldots ,k_{MA}\left(x_{n},\left(m,a\right)\right)\right)}^{⊤}$; and $\alpha_{f}={\left(K+\lambda_{f}I\right)}^{-1}y$, where $K\in R^{N\times N}$ with $K_{ij}=k_{MA}\left(x_{i},x_{j}\right)$, $y={\left(y_{1},\ldots ,y_{N}\right)}^{⊤}$.

A product kernel on (m,a) can capture interactions as in our case, where the effect of *m* on $\Omega_{L}$ depends on policy γ. So, we can establish a product kernel on (m,a) as follows:

$$k_{MA}^{\mathrm{prod}}\left(\left(m,a\right),\left(m^{\prime },a^{\prime }\right)\right)\;=\;\underset{k_{M}\left(m,m^{\prime }\right)}{\underset{︸}{\exp\;\left(-\frac{{\left(m-m^{\prime }\right)}^{2}}{2\;l_{M}^{2}}\right)}}\;\times \;\underset{k_{\Gamma }\left(a,a^{\prime }\right)}{\underset{︸}{\exp\;\left(-\frac{∥a-a^{\prime }{∥}^{2}}{2\;l_{\Gamma }^{2}}\right)}}.$$

Likewise, for the variance function $\sigma_{L}^{2}\left(m,\gamma \right)$, we compute the residuals $r_{i}=\omega_{Li}-{\hat{f}}_{L}\left(m_{i},\gamma_{i}\right)$; and we can define pseudo-responses $z_{i}=\log\left(r_{i}^{2}+\epsilon \right)$ with small ϵ>0, for numerical stability. Modeling $\log\;\sigma_{L}^{2}\left(m,\gamma \right)=v\left(m,\gamma \right)$ in an RKHS, guarantees strict positivity after exponentiation, turns multiplicative scale effects into additive structure, and improves residual behavior under Gaussian assumptions. Therefore, we fit KRR on (m,γ)→z and solve

(42) $${\hat{v}}_{L}\left(m,\gamma \right)\in \arg\min_{v\;\in H_{MA}}\;{\left(z_{i}-v\left(m_{i},\gamma_{i}\right)\right)}^{2}\;+\;\lambda_{v}\;{∥v∥}_{H_{m\gamma }}^{2}.$$

As before, $v\left(\cdot \right)=\sum_{i\in [N]}\alpha_{vi}k_{MA}\left(x_{j},\cdot \right)$ with $\alpha_{v}={\left(K+\lambda_{v}I\right)}^{-1}z$ and $z={\left(z_{1},\ldots ,z_{n}\right)}^{⊤}$. We predict $\hat{v}\left(m,a\right)$ and set ${\hat{\sigma }}_{L}^{2}\left(m,a\right)=\exp\left(\hat{v}\left(m,a\right)\right)>0$.

Using the above, we can now estimate $\mu_{L}\left(\omega ,m,a\right)=E[Y|\Omega_{L}=\omega ,\;M=m,\;\Gamma =a]$. We can use a weighted KRR formulation to compute ${\hat{\mu }}_{L}\left(\omega ,m,a\right)$ with observations $x_{i}=\left(\omega_{i},m_{i},a_{i}\right)$, and output $y_{i}=Y_{i}$, i=1,…,n. Let $k_{X}$ be a positive-definite kernel on x=(ω,m,a) with RKHS $H_{X}$. Then, we solve the following weighted regularized least-squares problem:

(43) $${\hat{\mu }}_{L}\left(x\right)\in \arg\min_{\mu \in H_{X}}\;\sum_{i=1}^{n}w_{i}\left(\gamma \right)\;{\left(y_{i}-\mu \left(x_{i}\right)\right)}^{2}\;+\;\lambda_{\mu }\;{∥\mu ∥}_{H_{X}}^{2},$$

employing $\mu \left(\cdot \right)=\sum_{i\in [N]}\alpha_{\mu i}\;k_{X}\left(x_{i},\cdot \right)$, $\alpha_{\mu }={\left(KW+\lambda I_{n}\right)}^{-1}W\;y$; thus, ${\hat{\mu }}_{L}\left(x\right)=k_{x}^{⊤}\alpha$, where $K_{ij}=k_{X}\left(x_{i},x_{j}\right)$, $K\in R^{n\times n}$, $W=\mathrm{diag}\left(w_{1}\left(\gamma \right),\ldots ,w_{n}\left(\gamma \right)\right)$, $y={\left(y_{1},\ldots ,y_{n}\right)}^{⊤}$ and $k_{x}={\left[k_{X}\left(x_{1},x\right),\ldots ,k_{X}\left(x_{n},x\right)\right]}^{⊤}$. The policy weights $w_{i}\left(\gamma \right)\geq 0$ with $\sum_{i}w_{i}\left(\gamma \right)=1$, prioritize fidelity near γ. More details about these weights are given in Section 3.3.4.

#### 3.3.3. Computing the Inner Gaussian Expectation

So far, we have estimated what appears within the curly brackets before integration, in the identification Formula (29), shown again below for clarity:

$$\begin{array}{cc} V(\gamma ) & =\int \left\{\int \mu_{L}\left(\omega_{L},m,\gamma \right)\;p\left(\omega_{L}|m,\Gamma =\gamma \right)\;d\omega_{L}\right\}\;p\left(m|\Gamma =\gamma \right)\;dm\\ \; & =\int \left\{E_{U_{\Omega_{L}}}\left[\mu_{L}\left(\gamma \left(m\right)+U_{\Omega_{L}},m,\gamma \right)\right]\right\}\;p\left(m|\Gamma =\gamma \right)\;dm, \end{array}$$

where $\mu_{L}\left(\omega_{L},m,\gamma \right):=\;E\left[Y|\Omega_{L}=\omega_{L},M=m,\Gamma =\gamma \right]$. That is, we show before how to estimate $\mu_{L}\left(\omega_{L},m,\gamma \right)$ and $p(\omega_{L}|m,\Gamma =\gamma )$ and now we show how to approximate

$$\begin{array}{cc} \int \mu_{L}\left(\omega_{L},m,\gamma \right)\;p\left(\omega_{L}|m,\Gamma =\gamma \right)\;d\omega_{L} & =E_{U_{\Omega_{L}}}\left[\mu_{L}\left(\gamma \left(m\right)+U_{\Omega_{L}},m,\gamma \right)\right]\\ \; & =E_{U_{\Omega_{L}}}\left[\mu_{L}\;\left(\Omega_{L},\;m,\;\gamma \right)\;|\;M=m,\Gamma =\gamma \right]\\ \; & =g_{U_{\Omega_{L}}}\left(m;\gamma \right). \end{array}$$

An efficient and natural way to compute the inner Gaussian expectation ${\hat{g}}_{U_{\Omega_{L}}}\left(m;\gamma \right)$ is by the Gauss–Hermite (GH) quadrature method. The integral for $g_{U_{\Omega_{L}}}\left(m;\gamma \right)$ can be expressed as

$$g_{U_{\Omega_{L}}}\left(m;\gamma \right)=\int_{-\infty }^{\infty }\mu_{L}\left(\omega_{L},\;m,\;\gamma \right)\;\frac{1}{\sqrt{2\pi }\;\sigma_{\Omega_{L}}}\exp\;\left(-\frac{{\left(\omega_{L}-\mu_{\Omega_{L}}\right)}^{2}}{2\sigma_{\Omega_{L}}^{2}}\right)\;d\omega_{L},$$

where $\mu_{\Omega_{L}}:={\hat{f}}_{L}\left(\gamma ,m\right)$, $\sigma_{\Omega_{L}}:=\sigma_{L}\left(\gamma ,m\right)>0$, which we previously estimate and $p\left(\Omega_{L}|\left(M=m,\Gamma =\gamma \right)\right)∼N\;\left(\mu_{\Omega_{L}},\sigma_{\Omega_{L}}^{2}\right)$. Writing $\Omega_{L}=\mu_{\Omega_{L}}+U_{L}$ with $U_{L}∼N\left(0,\sigma_{\Omega_{L}}^{2}\right)$, the change of variables $U_{L}=\sigma_{\Omega_{L}}Z$, i.e., $Z=U_{L}/\sigma_{\Omega_{L}}$, transforms the expectation to the standard-normal scale. No information is lost; it is the same integral expressed in a convenient form for the GH method. Therefore, with a standard normal Z∼N(0,1), equivalently, we have

(44) $$g_{Z}\left(m;\gamma \right)=E_{Z}\left[\mu_{L}\left(\mu_{\Omega_{L}}+\sigma_{\Omega_{L}}Z,\;m,\;\gamma \right)\right]=\int_{-\infty }^{\infty }\mu_{L}\left(\mu_{\Omega_{L}}+\sigma_{\Omega_{L}}z,\;m,\;\gamma \right)\;\frac{e^{-z^{2}/2}}{\sqrt{2\pi }}\;dz.$$

The GH method approximates integrals of the form $\int_{-\infty }^{\infty }e^{-x^{2}}\;f\left(x\right)\;dx\approx \sum_{m=1}^{M}w_{m}^{GH}\;f\left(x_{m}^{GH}\right)$, where ${\left\{x_{m}^{GH},w_{m}^{GH}\right\}}_{m=1}^{M}$ are Hermite nodes and weights. To bring the standard-normal expectation (44) to this form, set $z=\sqrt{2}\;x$ ( $dz=\sqrt{2}\;dx$), yielding

(45)gZ(m;γ)=1π∫−∞∞μLμΩL+σΩL2x,m,γ︸f(xGH)e−x2︸GHweightswGHdx.(46)≈1π∑m=1MwmGHμLμΩ+σΩ2xj,m,γ,

where the computable approximation in (46) for the definite integral in (45) is given by applying a *M*-point rule GH. The sum of all the weights for the GH rule is always $\sum_{m=1}^{M}w_{m}^{GH}=\int_{-\infty }^{\infty }e^{-x^{2}}\;dx=\sqrt{\pi }$ and the Golub–Welsch Algorithm is a standard numerical method for computing the nodes and weights of any Gaussian quadrature rule, including the Gauss–Hermite quadrature. Unlike Monte Carlo, GH has no sampling variance; this stability is valuable when comparing many candidate policies γ. The inner integral is always one-dimensional (action noise), so GH is computationally light, and the nodes ${\left\{x_{m}^{GH},w_{m}^{GH}\right\}}_{m=1}^{M}$ are pre-computable and reusable.

#### 3.3.4. Policy-Local Empirical Measure Construction in the Outer Integral

Finally, the identified value of a fixed policy γ can be written as a conditional expectation $E\;\left[g_{\gamma }\left(M\right)|\Gamma =\gamma \right]$, since:

$$\begin{array}{cc} V(\gamma ) & =\int \left\{E_{U_{\Omega_{L}}}\left[\mu_{L}\left(\gamma \left(m\right)+U_{\Omega_{L}},m,\gamma \right)\right]\right\}\;p\left(m|\Gamma =\gamma \right)\;dm,\\ \; & =\int g_{U_{\Omega_{L}}}\left(m;\gamma \right)\;p\left(m|\Gamma =\gamma \right)\;dm,\\ \; & =E\;\left[g_{\gamma }\left(M\right)|\Gamma =\gamma \right] \end{array}$$

So, the task is to estimate the expectation over the mediator distribution under policy γ. We estimate this by a local kernel smoother in policy space. We observe i.i.d. ${\left\{\left(m_{i},\gamma_{i},\ldots \right)\right\}}_{i=1}^{N}$ with realized policies $\gamma_{i}$ (e.g., $\gamma_{i}=\left(\alpha_{i},\beta_{i}\right)$). Because Γ is continuous, the event {Γ=γ} has probability zero; therefore, we approximate the conditional expectation by *localizing* to policies $\gamma_{i}$ that are close to γ and form a policy-local empirical distribution over the observed mediators $\left\{M_{i}\right\}$. For that policy-local empirical distribution, we may define a distance $d(\gamma_{i},\gamma )$ in the policy space. Since $\gamma_{i},\gamma$ lie in an RKHS $H_{k}$, we use $d\left(\gamma_{i},\gamma \right)={∥\gamma_{i}-\gamma ∥}_{H_{k}}$ to reflect the policy geometry. Additionally, we select a non-negative, radial kernel K(u)≥0 with u=d/h and a bandwidth h>0 as the Gaussian kernel, also known as the Radial Basis Function (RBF) kernel $K\left(u\right)=\exp\left(-u^{2}/2\right)$. So, as in the linear parametrized instance before, we define the unnormalized weights:

(47) $${\tilde{w}}_{i}\left(\gamma ;h\right)=K\;\left(\frac{d(\gamma_{i},\gamma )}{h}\right),\;w_{i}\left(\gamma ;h\right)=\frac{{\tilde{w}}_{i}\left(\gamma ;h\right)}{\sum_{j=1}^{n}{\tilde{w}}_{j}\left(\gamma ;h\right)}\geq 0,\;\mathrm{with}\;\sum_{i\in [N]}w_{i}=1,$$

Thus, we approximate $E\;\left[g_{\gamma }\left(M\right)|\Gamma =\gamma \right]$ using a policy-local empirical measure supported on the observed mediators for the conditional p(m∣Γ=γ). The policy-local empirical distribution for approximating p(m∣Γ=γ) is defined as

(48) $${\hat{P}}_{\gamma }^{\left(h\right)}\left(dm\right)=\sum_{i=1}^{n}w_{i}\left(\gamma ;h\right)\;\delta_{m_{i}}\left(dm\right),$$

where $\delta_{Mm_{i}}$ is the unit mass at $m_{i}$. Intuitively, ${\hat{P}}_{\gamma }^{\left(h\right)}$ is the law of *M* one would obtain by resampling historical mediators with probability proportional to how close their policies $\gamma_{i}$ are to the target γ. This approximation for p(m∣Γ=γ) turns the outer integral into an interpretable, positivity–respecting kernel average. Integrating the test function ${\hat{g}}_{U_{\Omega_{L}}}\left(\cdot ;\gamma \right)$ against this measure yields

(49)V^(γ)=E^[gγ(M)∣Γ=γ](50)=∫g^UΩL(m;γ)P^γ(h)(dm)(51)=∑i=1nwi(γ;h)g^(Mi;γ),

which is precisely the outer half of the nested g-computation estimator $\hat{V}\left(\gamma \right)$. The policy-local reweighting in (49)–(51) implicitly requires a *positivity (overlap)* condition: for any target policy γ, there must be sufficient probability mass of logged policies in a neighborhood of γ so that the conditional law p(M∣Γ=γ) can be well approximated by the empirical measure ${\hat{P}}_{\gamma }^{\left(h\right)}$. In finite samples, this induces an *empirical support constraint*: $\hat{V}\left(\gamma \right)$, which is reliable only if the weights $w_{i}\left(\gamma ;h\right)$ do not degenerate. We quantify local support via the *effective sample size* (ESS):

$$\mathrm{ESS}\left(\gamma ;h\right)\;:=\;\frac{{\left(\sum_{i=1}^{N}w_{i}\left(\gamma ;h\right)\right)}^{2}}{\sum_{i=1}^{N}w_{i}{\left(\gamma ;h\right)}^{2}}.$$

The ESS is a measure of how many independent samples the weighted sample set is equivalent to. A low ESS indicates high variance and instability in the estimate $\hat{V}\left(\gamma \right)$ because the estimate is dominated by a small number of data points. We also define the *support set*:

$$S_{\mathrm{support}}\left(h,\tau ,w_{\max}\right)\;:=\;\left\{\gamma \in \Gamma :\;\mathrm{ESS}\left(\gamma ;h\right)\geq \tau ,\;\max_{i}\;w_{i}\left(\gamma ;h\right)\leq w_{\max}\right\}.$$

The support set $S_{\mathrm{support}}$ defines the region in the policy space Γ where the estimate $\hat{V}\left(\gamma \right)$ is considered empirically reliable. Policies γ outside this set are not trustworthy due to a lack of data overlap. The ESS(γ;h)≥τ condition ensures that the evaluation is based on a sufficiently large effective sample size, preventing the estimate from being dominated by just a few data points. By requiring ESS(γ;h)≥τ, we enforce a degree of overlap between the target policy γ and the logged policies. Higher τ means we demand better overlap and a more stable estimate, which shrinks the support set $S_{\mathrm{support}}$. The $\max_{i}\;w_{i}\left(\gamma ;h\right)\leq w_{\max}$ condition directly guards against extreme importance weights, which are a hallmark of poor overlap or model mismatch in off-policy evaluation. This constraint helps bound the influence of rare, yet highly weighted, events. Policies γ that require assigning an enormous weight to any single data point are excluded from the support set. Lower $w_{\max}$ means we tolerate less weight variance and demand better weight balance, which also shrinks the support set $S_{\mathrm{support}}$. The effective sample size (ESS) serves as a proxy for uncertainty in the outer expectation. A small ESS suggests high estimator variance and low information, which necessitates a trust-region restriction in policy space. Conversely, a large ESS indicates data-supported proposals and reduced uncertainty.

## 4. A Functional Bayesian Optimization Algorithm for Single-Stage Canonical PAPs

Functional Bayesian Optimization (FBO) is the version of Bayesian Optimization (BO) (see Appendix C) in which the domain D of the objective function to maximize $f_{obj}$: D→R is a space of functions. We leverage the sequential optimization process of Bayesian optimization and the use of the Gaussian Process Upper Confidence Bound (GP-UCB) acquisition function (see Appendix C.2) to solve the single-stage canonical $PAP_{L_{1}F_{1}}$. Specifically, we propose an FBO algorithm to sequentially solve the following optimization problem.

(52) $$\gamma^{*}\in \underset{\gamma^{t}\in \Gamma }{\arg\max}\;E\left[J_{L}|do\left(\Gamma =\gamma^{t}\right)\right].$$

This is an objective functional, since its domain is a space of incentive functions Γ. We employ a GP-UCB acquisition functional (see Appendix C.2 and Section 4.2) in the proposed FBO algorithm as the strategy to guide the selection of the best next incentive function evaluation $\gamma^{t}$ in the sequential optimization of Equation (52). Additionally, we use a GP-UCB acquisition functional to establish upper bounds on the cumulative regret $R^{T}$ for sequentially optimizing the objective functional in Equation (52) with horizon *T* when the FBO algorithm described in the following subsections is employed.

In order to describe the proposed FBO algorithm to solve Equation (52), we begin by establishing a functional Gaussian process as a surrogate function model for the objective functional in Section 4.1. For this, we set up a functional space for the incentive functions space Γ, which allows the definition of a Gaussian process kernel over this space of functions in Section 4.1.1, and in light of this, we show how to compute the posterior distribution for this functional Gaussian process in Section 4.1.2. Later, the GP-UCB acquisition functional for functional search is described in Section 4.2. Having these elements available, we present, in Section 4.3, Algorithm 1 to solve Equation (52). This algorithm is referred to as the Stackelberg Functional Causal Bayesian Optimization (FCBO) algorithm for $PAP_{L1F1}$. In Section 4.4, we show cumulative-regret bounds results in terms of differential information gain for the Stackelberg FCBO algorithm.

**Algorithm** **1:** Stackelberg FCBO for single-stage 
$PAP_{L1F1}$ **Input    :** 
$D^{-1}=\{(\gamma^{i},\omega_{F}^{i},\omega_{L}^{i},J_{L}^{i}{)\}}_{i=1}^{N}$; the 
$H_{k}$ specification; the horizon *T*;                the functional GP kernel *K* on 
$\Gamma \subset H_{k}$; the exploration schedule 
${\left\{\beta_{t}\right\}}_{t=1}^{T}$;                and the CGM 
$M_{L1F1}$ for a 
$PAP_{L1F1}$ from the principal’s perspective. **Output** **:** 
$D^{T}={\left\{\left(\gamma^{t},E^{\gamma^{t}}\left[J_{L}^{t}\right]\right)\right\}}_{t\in [T]}$, with 
$\gamma^{t}\in H_{k}^{d}$ or 
$\gamma^{t}\in H_{k}$
**1**
.
**2**
Initialize functional GP 
$\mathrm{GP}\left(\mu^{0}\left(\gamma \right),K^{0}\left(\gamma ,\gamma^{\prime }\right)\right)$;
**3**
**for** 
t=1,…,T **do**
**4**

Select 
$\gamma^{t}\in \underset{\gamma \in H_{k}}{\arg\max\;}\mu^{t}\left(\gamma \right)+\beta^{t}\sigma^{t}\left(\gamma \right)$ (see Section 4.2);
**5**

Estimate 
$E^{\gamma^{t}}\left[J_{L}\right]$ as 
$\hat{V}\left(\gamma^{t}\right)=\hat{E}\left[J_{L}|do\left(\Gamma =\gamma^{t}\right)\right]$
(see Section 3.3);
**6**

Set 
$D^{t}\leftarrow D^{t-1}\cup \left\{\left(\gamma^{t},E^{\gamma^{t}}\left[J_{L}^{t}\right]\right)\right\}$;
**7**

Update functional GP 
$\mathrm{GP}\left(\mu^{t}\left(\gamma \right),K^{t}\left(\gamma ,\gamma^{\prime }\right);D^{t}\right)$ (see Equation (55));
**8**

**end**

**9**
**return** 
$D^{T}={\left\{\left(\gamma^{t},E^{\gamma^{t}}\left[J_{L}^{t}\right]\right)\right\}}_{t\in [T]}$

### 4.1. Functional Gaussian Process Surrogate Model

We employ a Reproducing Kernel Hilbert Space (RKHS) as a space of functions for the domain of the objective functional $E^{\gamma^{t}}\left[J_{L}\right]$: Γ→R in Equation (52). Taking Γ as $H_{k}$, that is, $E^{\gamma^{t}}\left[J_{L}\right]$: $H_{k}\to R$, where $H_{k}$ is an RKHS with reproducing kernel *k*: X×X→R and X⊆R (see Appendix B), allows us to directly define a Gaussian process kernel over functions in $H_{k}$ (see Section 4.1.1). In each round *t*, an incentive function $\gamma^{t}\in H_{k}$ is selected by means of a GP-UCB acquisition functional (see Section 4.2) and a noisy evaluation $E^{\gamma^{t}}\left[J_{L}\right]$ is returned.

We use a Gaussian process (GP) as a surrogate model for the objective functional $E^{\gamma^{t}}\left[J_{L}\right]$: $H_{k}\to R$. We assume specifically that the input function space is an RKHS $H_{k}$, which allows us to define a GP kernel over functions in a RKHS function space in Section 4.1.1. Therefore, the GP we use for this objective functional with domain $H_{k}$ is given as a stochastic process $F_{\gamma }=\left\{f\left(\gamma \right)|\gamma \in H_{k}\right\}$, in which every finite collection of random variables f(γ) has a multivariate normal distribution. So, we use this GP $F_{\gamma }=\left\{f\left(\gamma \right)|\gamma \in H_{k}\right\}$, with $F_{\gamma }∼\mathrm{GP}\left(\mu \left(\gamma \right),K\left(\gamma ,\gamma^{\prime }\right)\right)$ as a surrogate model for $E^{\gamma^{t}}\left[J_{L}\right]$. Next, based on [6], we formulate a GP kernel $K\left(\gamma ,\gamma^{\prime }\right)=cov\left[f\left(\gamma \right),f\left(\gamma^{\prime }\right)\right]$ with $\gamma ,\gamma^{\prime }\in H_{k}$. See [4] for GP fundamentals and [6,25] for functional BO surrogates.

#### 4.1.1. Functional Gaussian Process Kernel

Based on the RKHS $H_{k}$ kernel *k*, the GP kernel $K\left(\gamma ,\gamma^{\prime }\right)=cov\left[f\left(\gamma \right),f\left(\gamma^{\prime }\right)\right]$ can be constructed. We focus here on the construction of a functional version of the standard Radial Basis Function (RBF) kernel, which we assume in the regret analysis of this functional Bayesian framework.

The functional RBF can be stated as in Equation (53), where ${∥\cdot ∥}_{H_{k}}$ is the norm in the RKHS $H_{k}$, so ${∥\gamma ,\gamma^{\prime }∥}_{H_{k}}^{2}$ is the squared distance between the functions $\gamma ,\gamma^{\prime }\in H_{k}$, which can be calculated as the internal product ${\langle \gamma -\gamma^{\prime },\gamma -\gamma^{\prime }\rangle }_{H_{k}}$ in the RKHS $H_{k}$.

(53) $$K\left(\gamma ,\gamma^{\prime }\right)=\exp\left(-\frac{{∥\gamma ,\gamma^{\prime }∥}_{H_{k}}^{2}}{2\sigma^{2}}\right).$$

Thus, the squared distance ${∥\gamma ,\gamma^{\prime }∥}_{H_{k}}^{2}$ in a functional RBF between two incentive functions γ and $\gamma^{\prime }$ is given by Equation (54), with $\upsilon_{i},\upsilon_{j},\xi_{i},\xi_{j}\in R$, n,m∈N, $x_{i},x_{j},x_{i}^{\prime },x_{j}^{\prime }\in X$.

(54) $${\langle \gamma -\gamma^{\prime },\gamma -\gamma^{\prime }\rangle }_{H_{k}}=\sum_{i\in [n]}\sum_{j\in [n]}\upsilon_{i}\upsilon_{j}k\left(x_{i},x_{j}\right)-2\sum_{i\in [n]}\sum_{j\in [m]}\upsilon_{i}\xi_{j}k\left(x_{i},x_{j}^{\prime }\right)+\sum_{i\in [m]}\sum_{j\in [m]}\xi_{i}\xi_{j}k\left(x_{i}^{\prime },x_{j}^{\prime }\right).$$

We reserve lower-case $k(x,x^{\prime })$ for the reproducing kernel of the input RKHS $H_{k}$ over *X*, and upper-case $K(\gamma ,\gamma^{\prime })$ for the Gaussian-process kernel over incentive functions $\Gamma =H_{k}$. Thus, *k* appears only inside ${∥\cdot ∥}_{H_{k}}$ and expansions like Equation (54), while *K* and its posterior $K^{t}$ govern GP means, covariances, and Gram matrices.

#### 4.1.2. Functional Gaussian Process Posterior Distribution

After building the functional RBF kernel primarily by finding out how to measure the distance between the elements γ of the function space $H_{k}$, the posterior distribution of the functional GP $F_{\gamma }∼\mathrm{GP}\left(\mu \left(\cdot \right),K\left(\cdot ,\cdot \right)\right)$ may be updated in a way very similar to a scalar GP. The posterior distribution is again a GP $F_{\gamma }∼\mathrm{GP}\left(\mu \left(\cdot \right),K\left(\cdot ,\cdot \right)\right)$, with functional mean $\mu^{t}\left(\cdot \right)$ and covariance kernel $K^{t}\left(\cdot ,\cdot \right)$ given as follows:

(55) $$\begin{array}{c} \begin{array}{cc} \mu^{t}\left(\gamma \right)= & k^{t}{\left(\gamma \right)}^{⊤}{\left(G^{t}+\sigma^{2}I\right)}^{-1}y^{t},\\ K^{t}\left(\gamma ,\gamma^{\prime }\right)= & K\left(\gamma ,\gamma^{\prime }\right)-k^{t}{\left(\gamma \right)}^{⊤}{\left(G^{t}+\sigma^{2}I\right)}^{-1}k^{t}\left(\gamma^{\prime }\right), \end{array} \end{array}$$

where $k^{t}\left(\gamma \right)={\left[K\left(\gamma ,\gamma^{1}\right),K\left(\gamma ,\gamma^{2}\right),\ldots ,K\left(\gamma ,\gamma^{t}\right)\right]}^{⊤}$, $G^{t}$ is the t×t Gram matrix with entries $G_{t}\left[i,j\right]=K\left(\gamma_{i},\gamma_{j}\right)$, for i,j∈[t] (see Appendix B), and $y^{t}={\left[y^{1},\ldots ,y^{t}\right]}^{⊤}$ is the vector of estimations $y^{1}=E^{\gamma^{1}}\left[J_{L}\right],\ldots ,y^{t}=E^{\gamma^{t}}\left[J_{L}\right]$.

### 4.2. Upper Confidence Bound Acquisition Functional

The functional version of the GP-UCB acquisition function (see Appendix C.2, Equation (A20)) is given in Equation (56):

(56) $$\alpha_{\text{fGP-UCB}}\left(\gamma ;D,\beta^{t}\right)=\mu^{t}\left(\gamma \right)+\beta^{t}\sigma^{t}\left(\gamma \right),$$

where $\sigma^{t}\left(\gamma \right)=\sqrt{K^{t}\left(\gamma ,\gamma \right)}$ (see Equation (55)). This acquisition functional can be interpreted as the strategy to choose an incentive function as follows:

(57) $$\gamma^{t+1}=\underset{\gamma \in H_{k}}{\arg\max}\;\mu^{t}\left(\gamma \right)+\beta^{t}\sigma^{t}\left(\gamma \right),$$

which if we remove the second term $\beta^{t}\sigma^{t}\left(\gamma \right)$ and optimize just for $\mu^{t}\left(\gamma \right)$, i.e., maximizing the expected reward based on the posterior distribution so far, this rule would be too greedy too soon and would lead to getting stuck in shallow local optima. Instead, the full objective $\mu^{t}\left(\gamma \right)+\beta^{t}\sigma^{t}\left(\gamma \right)$ in Equation (57) prefers both incentive functions γ where f(γ) is uncertain, that is, with large $\sigma^{t}\left(\gamma \right)$, and where we expect to achieve high rewards, i.e., with large $\mu^{t}\left(\gamma \right)$. Thus, it implicitly deals with the trade-off between exploration and exploitation. An interpretation of this intervention rule is that it greedily selects the intervention functions γ such that f(γ) should be a reasonable upper bound on $f(\gamma^{*})$, where $\gamma^{*}$ is the optimal incentive function (see Appendix C.2; this follows the GP-UCB principle [7]). The UCB term $\beta_{t}^{1/2}\sigma^{t-1}\left(\gamma \right)$ promotes policies with high expected information gain about the functional value. As shown in the regret analysis in Section 4.4, this connects the functional acquisition directly to *mutual-information* control. Our analysis offers theoretical guidance for acquisition, specifically through trust-region methods using ESS and weight caps. Empirical tuning and benchmarking are intentionally deferred to a companion, application-driven study.

### 4.3. The Stackelberg FCBO Algorithm for Single-Stage Canonical PAPs

We present a Functional Causal Bayesian Optimization (FCBO) algorithm for the single-stage $PAP_{L1F1}$ by integrating all components of Section 4.1 and Section 4.2. We refer to this algorithm, shown in Algorithm 1, as the Stackelberg FCBO algorithm for the single-stage $PAP_{L1F1}$. Recall that Algorithm 1 addresses the optimization problem of Equation (52).

Algorithm 1 gets as input a data set of past observations of the system, $D^{-1}=\{(\gamma^{i},\omega_{F}^{i},\omega_{L}^{i},J_{L}^{i}{)\}}_{i=1}^{N}$; the specification of the incentive function space given as an RKHS $H_{k}$, where $\gamma^{t}\in H_{k}^{d}$ (finite function space) or $\gamma^{t}\in H_{k}$ (infinite function space); the number of rounds *T* contemplated in the sequential optimization; the kernel *K* of the functional GP; the exploration schedule ${\left\{\beta_{t}\right\}}_{t=1}^{T}$; and the CGM $M_{L1F1}$ for a $PAP_{L1F1}$ from the principal’s perspective. The functional space $H_{k}$ can be specified by stating the reproducing kernel *k* of the RKHS $H_{k}$ (see Appendix B). In addition, the functional GP kernel *K* over $\Gamma \subset H_{k}$ must be specified (by default, we can use the functional RBF kernel of Equation (53)). In Algorithm 1, we use a time-indexed exploration coefficient $\beta^{t}$ to match the high-probability guarantees in Section 4.4. For these results, we instantiate

$$\beta^{t}\;=\;2\log\;\left(\frac{t^{2}\pi^{2}\;\left|\Gamma \right|}{6\;\delta }\right),$$

so, the acquisition is $\mu^{t}\left(\gamma \right)+\beta^{t}\sigma^{t}\left(\gamma \right)$. In practice, one may instead tune a constant confidence level ρ and set $\beta =\Phi^{-1}\left(\rho \right)$ (where $\Phi^{-1}$ is the quantile function, i.e., the inverse cumulative distribution function (CDF); see Appendix C.2). This is equivalent to using a constant exploration coefficient $\beta^{t}\equiv \beta$. Our regret bounds in Section 4.4 hold whenever the run uses the exploration schedule ${\left\{\beta^{t}\right\}}_{t=1}^{T}$ as $\beta^{t}=2\log\left(\frac{t^{2}\pi^{2}\left|\Gamma \right|}{6\delta }\right)$ or any schedule that dominates it pointwise. We can set the exploration schedule in this way for default in Algorithm 1.

The $M_{L1F1}$ from the principal’s perspective have the induced DAG, from the set of observable variables $O_{L}=\left\{\Gamma ,\Omega_{L},\Omega_{F},J_{L}\right\}$, with arrows $\Gamma \to \Omega_{L}$, $\Gamma \to \Omega_{F}$, $\Omega_{F}\to \Omega_{L}$, $\Omega_{L}\to J_{L}$ and $\Omega_{F}\to J_{L}$, as causal structure. Additionally, the follower utility $J_{F}$ is an unobservable or hidden variable, and the follower mechanism $f_{\Omega_{F}}\left(\gamma ,U_{\Omega_{F}}\right)$ is a black box function. Algorithm 1 returns the accumulated history data set $D^{T}$ after *T* rounds in the sequential optimization:

(58) $$D^{T}=\left\{\left(\gamma^{1},E^{\gamma^{1}}\left[J_{L}\right]\right),\ldots ,\left(\gamma^{T},E^{\gamma^{T}}\left[J_{L}\right]\right)\right\}.$$

Modeling f(γ) as a sample from $F_{\gamma }=\left\{f\left(\gamma \right)|\gamma \in H_{k}\right\}$ with $F_{\gamma }∼\mathrm{GP}\left(\mu \left(\gamma \right),K\left(\gamma ,\gamma^{\prime }\right)\right)$, as a surrogate model for the objective functional $E^{\gamma^{t}}\left[J_{L}\right]$: $H_{k}\to R$ (see Section 4.1). We completely specified this GP by its mean functional μ(γ)=E[f(γ)] and kernel functional $K\left(\gamma ,\gamma^{\prime }\right)=E\left[\left(f(\gamma )-\mu (\gamma )\right)\left(f\left(\gamma^{\prime }\right)-\mu \left(\gamma^{\prime }\right)\right)\right]$. We assume, without loss of generality (see [4]), a GP with mean function zero, i.e., μ≡0, as prior distribution over $F_{\gamma }$. So, we initialize the functional GP in line 2 with $\mu^{0}\left(\gamma \right)$ as the zero function and $K^{0}\left(\gamma ,\gamma^{\prime }\right)$ as the functional RBF in Equation (53), to begin the inductive process of Algorithm 1.

From Line 3 to Line 8, the sequential optimization is performed, going through *T* rounds. The decision on the incentive function $\gamma^{t}$ is made employing the GP-UCB acquisition functional (see Section 4.2) as a policy $\pi^{t}$: $D^{t-1}\to \gamma^{t}$. The expected value of $J_{L}$ on the post-intervention distribution $p\left(J_{L}|do\left(\Gamma =\gamma^{t}\right)\right)$ is estimated by computing $\hat{V}\left(\gamma^{t}\right)=\hat{E}\left[J_{L}|do\left(\Gamma =\gamma^{t}\right)\right]$, using the techniques developed in Section 3.3. The estimation of $E^{\gamma^{t}}\left[J_{L}\right]$, is the approach by which Algorithm 1 gets information about the objective functional of Equation (52) to guide the search for $\gamma^{*}$. To complete a round *t* in the sequential optimization process outlined in Algorithm 1, first the data set $D^{t}$ is updated in Line 6 by adding the new observation pair $\left(\gamma^{t},E^{\gamma^{t}}\left[J_{L}\right]\right)$. Then, in Line 7, the surrogate functional, i.e., the functional GP GP, for the objective functional of the optimization problem in Equation (52), is updated using $D^{t}$. Figure 2 complements Algorithm 1 with a flow-chart of one FCBO round. The support-aware GP-UCB step and the two-layer estimator are highlighted as novel elements.

Once the *T* rounds are completed, Algorithm 1 returns $D^{T}$. Observe that, despite the fact that the algorithm returns the cumulative history data set in Equation (58), it implicitly addresses the optimization problem of Equation (52), since the following holds for large *T*:

(59) $$\gamma^{*}\approx \gamma^{+}\in \underset{\left(\gamma^{t},E^{\gamma^{t}}\left[J_{L}\right]\right)\in D^{T}}{\arg\max}\left\{E^{\gamma^{1}}\left[J_{L}\right],\ldots ,E^{\gamma^{T}}\left[J_{L}\right]\right\}.$$

That is, due to the convergence result in Section 4.4 for Algorithm 1, we maintain the best incentive function $\gamma^{+}$ from $\left(\gamma^{+},E^{\gamma^{+}}\left[J_{L}\right]\right)\in D^{T}$ to solve Equation (52), as $\gamma^{+}$ approximates the optimum $\gamma^{*}$ of the functional optimization problem in Equation (52). Furthermore, we can bound the expected cumulative regret $E[R_{D^{T}}^{T}]$ using Algorithm 1 to address the singe stage canonical $PAP_{L1F1}$. This result is presented in the following subsection.

### 4.4. Information-Theoretic Regret Bounds on the Stackelberg FCBO Algorithm

The cumulative-regret bounds for Algorithm 1 are derived by adapting the regret bounds results in terms of differential information gain (see Section 4.4.1) from [7] to our problem setting. In our setting, each observation $y_{t}=\hat{V}\left(\gamma_{t}\right)$ aggregates information about an interventional query $V(\gamma_{t})$ on the underlying CGM. The resulting regret bounds therefore quantify how quickly an information-guided sequence of interventions $do(\Gamma =\gamma_{t})$ converges, in policy value, to the best available intervention in the considered class. First, in Section 4.4.2, we deal with the setting in which the set of admissible incentive functions Γ is finite. Specifically, we assume that Γ is represented as the finite RKHS $H_{k}^{d}$ provided in Appendix B, so |Γ|=d∈N. Then, we show how to extend the results for the finite case from Section 4.4.2 to the general case where Γ is an infinite RKHS $H_{k}$ in Section 4.4.3.

Recall from Section 4.1 that the objective functional in Equation (52) is viewed as a sample path *f* of the functional GP $F_{\gamma }=\left\{f\left(\gamma \right)|\gamma \in H_{k}\right\}$ with $F_{\gamma }∼\mathrm{GP}\left(\mu \left(\gamma \right),K\left(\gamma ,\gamma^{\prime }\right)\right)$. Algorithm 1 uses a centered GP $\mathrm{GP}\left(\mu \equiv 0,K(\gamma ,\gamma^{\prime })\right)$ as a prior distribution $\mathrm{GP}\left(\mu^{0}\left(\gamma \right),K^{0}\left(\gamma ,\gamma^{\prime }\right)\right)$ over $F_{\gamma }$. So, we are assuming a priori that the objective functional in Equation (52) is a sample path *f* from a centered GP $\mathrm{GP}\left(\mu \equiv 0,K(\gamma ,\gamma^{\prime })\right)$. The initialization of the functional GP in Algorithm 1 also specified $K^{0}\left(\gamma ,\gamma^{\prime }\right)$ as the functional RBF in Equation (53). However, following the analysis in [7], we do not assume a specific functional kernel.

At round *t*, the observation is $y_{t}=\hat{V}\left(\gamma_{t}\right)$ with estimation error $\varepsilon_{t}$: $=y_{t}-V\left(\gamma_{t}\right)$. We assume that ${\left\{\varepsilon_{t}\right\}}_{t\geq 1}$ forms a conditionally *sub-Gaussian martingale-difference* sequence with respect to the algorithm’s filtration $\left\{F_{t}\right\}$, i.e., $E[\varepsilon_{t}|F_{t-1}]=0$ and $E\left[\exp\left(\lambda \varepsilon_{t}\right)|F_{t-1}\right]\leq \exp\left(\lambda^{2}R^{2}/2\right)$ for all λ∈R and some envelope R>0 (cf. kernelized bandit analyses with conditionally sub-Gaussian noise [8]). The variance may depend on $\gamma_{t}$ (heteroskedasticity). For the analysis, we further assume a uniform bound $\mathrm{Var}\left(\varepsilon_{t}|F_{t-1}\right)\leq \sigma_{*}^{2}$ on the support set where the Stackelberg FCBO algorithm proposes candidates. Under this *uniform envelope*, the standard GP-UCB high-probability confidence sets and regret bounds apply, with $\sigma_{*}^{2}$ entering the information-gain and $\beta_{t}$ expressions (Section 4.4.1).

#### 4.4.1. Differential Information Gain

A primary concern in the regret analysis of Algorithm 1 is to measure the efficiency with which τ noisy observations $D^{\tau }=\left\{\left(\gamma^{1},E^{\gamma^{1}}\left[J_{L}\right]\right),\ldots ,\left(\gamma^{\tau },E^{\gamma^{\tau }}\left[J_{L}\right]\right)\right\}$, and acquire knowledge about the objective functional $E\left[J_{L}|do\left(\Gamma =\gamma^{t}\right)\right]$ in Equation (52). This learning process becomes apparent through the improvement of the GP surrogate model $\mathrm{GP}(\mu \left(\gamma \right),K\left(\gamma ,\gamma^{\prime }\right))$ for $E\left[J_{L}|do\left(\Gamma =\gamma^{t}\right)\right]$, after the observation process in which the Algorithm 1 decides on $\gamma_{t}$, by the GP-UCB acquisition functional policy $\pi^{t}$: $D^{t-1}\to \gamma^{t}$, and then, after the follower response $\omega_{F}$, computes $\hat{V}\left(\gamma^{t}\right)=\hat{E}\left[J_{L}|do\left(\Gamma =\gamma^{t}\right)\right]$ to obtain the observed pair $\left(\gamma^{t},E^{\gamma^{t}}\left[J_{L}\right]\right)$. The informativeness of this observation process, as a function of the number of observations τ, provides a way to bound the ability to learn about the objective functional $E\left[J_{L}|do\left(\Gamma =\gamma^{t}\right)\right]$.

Here, we use the notation ${\hat{E}}^{\gamma^{t}}\left[J_{L}\right]=E^{\gamma^{t}}\left[J_{L}\right]+\epsilon^{t}$, to explicitly distinguish between the assumed noisy observations ${\hat{E}}^{\gamma^{t}}\left[J_{L}\right]$ and exact observations $E^{\gamma^{t}}\left[J_{L}\right]$ on the objective functional $E\left[J_{L}|do\left(\Gamma =\gamma^{t}\right)\right]$. The information capacity of an arbitrary set of sampling points $\Gamma^{\tau }=\left\{\gamma^{1},\ldots ,\gamma^{\tau }\right\}\subset \Gamma$ regarding the objective functional $E\left[J_{L}|do\left(\Gamma =\gamma^{t}\right)\right]$ can be measured by the mutual information $I^{\tau }$, also known as information gain, between $f^{\tau }=\left\{E^{\gamma^{1}}\left[J_{L}\right],\ldots ,E^{\gamma^{\tau }}\left[J_{L}^{\tau }\right]\right\}$ and the noisy observations $y^{\tau }=\left\{{\hat{E}}^{\gamma^{1}}\left[J_{L}\right],\ldots ,{\hat{E}}^{\gamma^{\tau }}\left[J_{L}\right]\right\}$ at these points. That is, measuring the reduction in uncertainty over $E\left[J_{L}|do\left(\Gamma =\gamma^{t}\right)\right]$ resulting from the disclosure of $y^{\tau }$, which is calculated as in Equation (60).

(60) $$\begin{array}{c} \begin{array}{cc} I^{\tau }\left(f^{\tau };y^{\tau }\right) & =H\left(f^{\tau }\right)-H\left(f^{\tau }|y^{\tau }\right)\\ \; & =H\left(y^{\tau }\right)-H\left(y^{\tau }|f^{\tau }\right)=I^{\tau }\left(y^{\tau };f^{\tau }\right), \end{array} \end{array}$$

as mutual information is symmetric, and where $H(f^{\tau })$ denotes the differential entropy of $f^{\tau }$ and $H(y^{\tau }|f^{\tau })$ denotes the conditional differential entropy of $y^{\tau }$ given $f^{\tau }$. The differential entropy of a set of multivariate Gaussian distributed random variables is calculated as $H\left(X_{1},\ldots ,X_{n}\right)=H\left(N_{n}\left(\mu ,K\right)\right)=\frac{1}{2}\log[{\left(2\pi e\right)}^{n}\left|K|\right]$, where |K| denotes the determinant of the covariance matrix *K* (see [28]). Thus, we can write its covariance as $K^{\tau }+\sigma^{2}I$, that is, separating the additive noise into a diagonal matrix $\sigma^{2}I$, where $K^{\tau }=[K({\hat{E}}^{\gamma }\left[J_{L}\right],{\hat{E}}^{\gamma^{\prime }}\left[J_{L}\right]$ for every ${\hat{E}}^{\gamma }\left[J_{L}\right],{\hat{E}}^{\gamma^{\prime }}\left[J_{L}\right]\in y^{\tau }$. Then, we can compute $H(y^{\tau })$ as follows:

(61) $$H\left(y^{\tau }\right)=\frac{1}{2}\log[{\left(2\pi e\right)}^{\tau }|K^{\tau }+\sigma^{2}I\left|\right].$$

To compute $H(y^{\tau }|f^{\tau })$, observe that given $f^{\tau }$, the only randomness left is the noise, which has covariance $\sigma^{2}I$, so

(62) $$H\left(y^{\tau }|f^{\tau }\right)=\frac{1}{2}\log[{\left(2\pi e\right)}^{\tau }|\sigma^{2}I\left|\right]=\frac{1}{2}\log\left[{\left(2\pi e\right)}^{\tau }\sigma^{2\tau }\right].$$

Putting all together, the information gain $I^{\tau }\left(f^{\tau };y^{\tau }\right)$ is given by $\frac{1}{2}\log\left[|I+K^{\tau }\sigma^{-2}|\right]$ as shown next in Equation (63):

(63) $$\begin{array}{c} \begin{array}{cc} I^{\tau }\left(f^{\tau };y^{\tau }\right) & =H\left(y^{\tau }\right)-H\left(y^{\tau }|f^{\tau }\right)\\ \; & =\frac{1}{2}{\log[\left(2\pi e\right)}^{\tau }|K^{\tau }+\sigma^{2}I|]-\frac{1}{2}{\log[\left(2\pi e\right)}^{\tau }|\sigma^{2}I\left|\right]\\ & =\frac{1}{2}\log\left[\frac{|K^{\tau }+\sigma^{2}I|}{|\sigma^{2}I|}\right]=\frac{1}{2}\log\left[|I+K^{\tau }\sigma^{-2}|\right] \end{array} \end{array}$$

$$I\left(f_{t};y_{t}\right)=H\left(y_{t}\right)-H\left(y_{t}|f_{t}\right)=\frac{1}{2}\log\left|I+\sigma^{-2}K_{t}\right|$$

In our setting, we define the maximum information gain $I^{T}$ after *T* rounds as

(64) $$I^{T}\;=\;\frac{1}{2}\;\log\left|\;I\;+\;\Sigma_{T}^{-1}G^{T}\;\right|,\;\Sigma_{T}:=\mathrm{diag}\left(\sigma_{1}^{2},\ldots ,\sigma_{T}^{2}\right),$$

where I is the identity matrix and $G^{T}$ is the covariance matrix. For notational simplicity in the stated bounds we adopt the conservative specialization $\Sigma_{T}⪯\sigma_{*}^{2}I$, which yields $I_{T}\leq \frac{1}{2}\log\left|\;I+\sigma_{*}^{-2}G_{T}\right|$ and recovers the expressions in our regret constants.

After presenting the concept of mutual information on differential entropy to assess the informativeness with respect to the objective functional $E\left[J_{L}|do\left(\Gamma =\gamma^{t}\right)\right]$ of a noisy set of observations $y^{\tau }=\left\{{\hat{E}}^{\gamma^{1}}\left[J_{L}\right],\ldots ,{\hat{E}}^{\gamma^{\tau }}\left[J_{L}\right]\right\}$ at a certain set of sampling points $\Gamma^{\tau }=\left\{\gamma^{1},\ldots ,\gamma^{\tau }\right\}\subset \Gamma$. We are now able to present the regret analysis for Algorithm 1 in terms of information gain $I^{\tau }\left(f^{\tau };y^{\tau }\right)$.

#### 4.4.2. Regret Bound for a Finite Incentive Function Space

The cumulative regret in a sequential optimization process is the loss in reward resulting from the inability to predict the points that maximize the objective function, in advance. For Algorithm 1, this is the error between the decided incentive functions $\gamma^{1},\ldots ,\gamma^{T}$ and those that maximize the objective functional $E\left[J_{L}|do\left(\Gamma =\gamma \right)\right]$. That is, letting $\gamma^{*}\in \arg\max_{\gamma^{t}\in \Gamma }f\left(\gamma \right)$, with $f\left(\gamma \right)=E\left[J_{L}|do\left(\Gamma =\gamma^{t}\right)\right]$, for a incentive function choice $\gamma^{t}$ in round *t*, we incur instantaneous regret $r^{t}=f\left(\gamma^{*}\right)-f\left(\gamma^{t}\right)$, and the cumulative regret $R_{T}$ is defined as $R^{T}=\sum_{t=1}^{T}r^{t}$.

Theorem 1 establishes a probabilistic bound for the cumulative regret of Algorithm 1, ensuring its convergence and efficiency with high probability. Considering a confidence bound δ∈(0,1), the result of Theorem 1 is that the cumulative regret $R^{T}$ of Algorithm 1 is bound by the square root of a linear expression of the information gain $I^{T}\left(f^{T};y^{T}\right)$, with probability of at least 1−δ. Recall from previous Section 4.4.1 that information gain $I^{T}\left(f^{T};y^{T}\right)$ is a measure of the reduction in uncertainty over the objective functional, resulting from the disclosure of observations $y^{T}=\left\{{\hat{E}}^{\gamma^{1}}\left[J_{L}\right],\ldots ,{\hat{E}}^{\gamma^{T}}\left[J_{L}\right]\right\}$, generated by the decision of the incentive function, i.e., sampling points, $\Gamma^{\tau }=\left\{\gamma^{1},\ldots ,\gamma^{\tau }\right\}\subset \Gamma$. The linear expression for the information gain in the specified upper bound includes the low coefficients $\beta^{T}$ and $C_{1}$. The coefficient $\beta^{t}$ is the exploration coefficient in the acquisition functional (see Section 4.2) on the strategy to choose the next incentive function in Equation (57), which for the purpose of the bound we use $\beta^{t}=2\log\left(\frac{t^{2}\pi^{2}\left|\Gamma \right|}{6\delta }\right)$, for all t∈[T]. This choice reconciles the exploration coefficient used in Algorithm 1 with the regret analysis: any implementation that uses $\beta^{t}$ above (or a pointwise larger schedule) satisfies the bound of Theorem 1. For the auxiliary coefficient $C_{1}$, we use $C_{1}=\frac{4\sigma^{2}}{\log(1+\sigma^{-2})}$. Theorem 1 given below assumes the finite functional space $H_{k}^{d}$, which was established in the Appendix B, as the finite set of allowed incentive functions, i.e., $\Gamma =H_{k}^{d}$ so |Γ|=d for some d∈N. Recall from Section 4.1 that the objective functional in Equation (52) is viewed as a sample path *f* of the functional GP $F_{\gamma }=\left\{f\left(\gamma \right)|\gamma \in H_{k}\right\}$ with $F_{\gamma }∼\mathrm{GP}\left(\mu \left(\gamma \right),K\left(\gamma ,\gamma^{\prime }\right)\right)$. Assume further that the observation errors $\left\{\varepsilon_{t}\right\}$ are conditionally *R*-sub-Gaussian and form a martingale-difference sequence with a uniform variance envelope $\mathrm{Var}\left(\varepsilon_{t}|F_{t-1}\right)\leq \sigma_{*}^{2}$ over the proposal support.

**Theorem** **1.** *Let δ∈(0,1) and assume that *Γ is finite, with $\Gamma =H_{k}^{d}$ for some d∈N. Running Algorithm 1 using $\beta^{t}=2\log\left(\frac{t^{2}\pi^{2}\left|\Gamma \right|}{6\delta }\right)$ for a sample path $f\left(\gamma \right)=E\left[J_{L}|do\left(\Gamma =\gamma \right)\right]$ of a functional GP $F_{\gamma }=\left\{f\left(\gamma \right)|\gamma \in H_{k}^{d}\right\}∼\mathrm{GP}\left(\mu \left(\gamma \right),K\left(\gamma ,\gamma^{\prime }\right)\right)$ with mean function $\mu^{t}\left(\gamma^{t}\right)=0$ and covariance function $K^{t}\left(\gamma ,\gamma^{\prime }\right)$, we get a cumulative-regret bound of $O^{*}\sqrt{TI^{\tau }\left(f^{\tau };y^{\tau }\right)\log\left|\Gamma \right|}$ with high probability. Specifically,

$$\mathrm{Pr}\left\{R_{D^{T}}^{T}\leq \sqrt{C_{1}T\beta^{T}I^{T}\left(f^{T};y^{T}\right)}\;\right\}\geq 1-\delta ,$$

*for all T≥1, where $C_{1}=\frac{4\sigma^{2}}{\log(1+\sigma^{-2})}$.*

The proof of Theorem 1 is provided in Appendix A by demonstrating a series of partial results given as Propositions A1–A4.

#### 4.4.3. Regret Bound on an Infinite Incentive Function Space

The regret bound of Theorem 1 is established under the assumption that the set of admissible incentive functions Γ is finite, specifically $\Gamma =H_{k}^{d}$. In practice, however, the Stackelberg FCBO algorithm can operate over a continuous, potentially infinite-dimensional admissible set of incentive functions $\Gamma \subset H_{k}$, where $H_{k}$ is a bounded RKHS. In this subsection, we extend the cumulative-regret analysis of the Stackelberg FCBO algorithm to an infinite admissible space of incentive functions $\Gamma \subset H_{k}$ where $H_{k}$ is a bounded separable RKHS. This corresponds to the setting where incentive functions $\gamma^{t}\in \Gamma =H_{k}$ are sampled and optimized over a non-parametric space of continuous functions. In accordance with the infinite domain analysis in [7], we assume that the functional f(γ), with $f\left(\gamma \right)=E\left[J_{L}|do\left(\Gamma =\gamma \right)\right]$ is Lipschitz continuous and is a member of the RKHS that is induced by the functional kernel.

Applying the regret bounds of Theorem 1 to a finite subset of $H_{k}$ obtained by discretizing $H_{k}$ does not ensure that the regret bound is applicable to the original continuous infinite domain $H_{k}$. The issue is that the objective functional *f*: $H_{k}\to R$, even if continuous, may not be well approximated on all of the function space $H_{k}$ by its restriction to a finite set, and this would only control regret over the discretized surrogate problem, not the full functional optimization problem:

$$\gamma^{*}\in \underset{\gamma^{t}\in \Gamma \subset H_{k}}{\arg\max}\;E\left[J_{L}|do\left(\Gamma =\gamma^{t}\right)\right].$$

Furthermore, regret is accumulated over the true sequence of evaluations, which may lie outside any fixed discretization. So, to rigorously lift the regret guarantees from a finite discretization to the full RKHS domain $H_{k}$, we require uniform approximation of both (i) the infinite set of admissible incentive functions and (ii) the objective functional *f*: $H_{k}\to R$ itself. Therefore, the key step is to approximate the infinite-dimensional domain $H_{k}$ and the functional f(γ) in a way that allows the application of the previously developed finite-set regret analysis from Theorem 1.

In order to overcome this, we approximate the infinite function space by constructing a finite-dimensional subspace by means of two applications of the Stone–Weierstrass theorem (see Appendix D). In the first application, the objective functional *f*: Γ→R, with $\Gamma \subset H_{k}$ compact, is uniformly approximated on Γ by finite linear combinations of kernel sections $K(\cdot ,\gamma_{i})$ associated with a carefully chosen finite set of basis incentive functions $\left\{\gamma_{1},\ldots ,\gamma_{d}\right\}\subset H_{k}$. As a consequence, for every $\varepsilon_{1}>0$, there exists such a finite set and coefficients $\alpha_{1},\ldots ,\alpha_{d}\in R$ satisfying

$$\underset{\gamma \in \Gamma }{\sup}\left|f\left(\gamma \right)-\sum_{i=1}^{d}\alpha_{i}\;K\left(\gamma_{i},\gamma \right)\right|<\varepsilon_{1},$$

and the set ${\left\{\gamma_{i}\right\}}_{i=1}^{d}\subset \Gamma$ then acts as a basis of reference incentive functions, and the approximant $\sum_{i=1}^{d}\alpha_{i}K\left(\gamma_{i},\gamma \right)$ providing a finite-dimensional parametric model for *f*. Details of this construction are given in Appendix D, where an explanation of two formal methods for selecting such basis incentive functions (one based on minimal ε-covers and one based on spectral decomposition of the kernel operator) is also provided in Appendix D.2.

From the first application of the Stone–Weierstrass theorem, we obtained a finite set of basis incentive functions $\left\{\gamma_{1},\ldots ,\gamma_{d}\right\}\subset H_{k}$ and coefficients $\left\{\alpha_{i}\right\}$ such that f(γ) is uniformly approximated, to within an error $\varepsilon_{1}>0$, by

$$\sum_{i=1}^{d}\alpha_{i}\;K\left(\gamma_{i},\gamma \right).$$

In the second application, each basis function $\gamma_{i}$ is itself uniformly approximated on the compact input domain $\Omega_{F}$, the input space of the follower’s decision variable, by a polynomial $p_{i}^{\left(n\right)}$ of degree at most *n*, so that the entire approximation to *f* is parameterized by finitely many coefficients. That is, we now apply the Stone–Weierstrass theorem a second time, but this time to each basis function $\gamma_{i}$ individually, treating it as a continuous scalar-valued function on the compact domain $\Omega_{F}$. Let $P_{n}$ denote the algebra of real polynomials on $\Omega_{F}$ of degree at most *n*. The Stone–Weierstrass theorem on $\Omega_{F}$, compact in the Euclidean topology, ensures that $P_{n}$ is uniformly dense in $C(\Omega_{F},R)$. Therefore, for any $\varepsilon_{2}>0$ and for each i∈{1,…,d}, there exists a polynomial $p_{i}^{\left(n\right)}\in P_{n}$ such that

$$\underset{x\in \Omega_{F}}{\sup}|\gamma_{i}\left(x\right)-p_{i}^{\left(n\right)}\left(x\right)|<\varepsilon_{2}.$$

Therefore, each $\gamma_{i}$ can be replaced by a polynomial $p_{i}^{\left(n\right)}$ of degree ≤n with uniform approximation error $\varepsilon_{2}$ on $\Omega_{F}$. So, the resulting functional approximation becomes

$$f\left(\gamma \right)\approx \sum_{i=1}^{d}\alpha_{i}\;K\left(p_{i}^{\left(n\right)},\gamma \right),$$

where *K* is evaluated between the polynomial surrogate $p_{i}^{\left(n\right)}$ and the input γ.

Replacing each $\gamma_{i}$ with $p_{i}^{\left(n\right)}$ parameterises the basis incentive functions by their polynomial coefficients. So, the full parameterisation of the approximant to *f* now consists of (1) the *d* coefficients $\left\{\alpha_{i}\right\}$ from the first approximation step, and (2) the coefficients of the *d* polynomials $\left\{p_{i}^{\left(n\right)}\right\}$, each with finitely many coefficients determined by its degree *n*. This yields a finite-dimensional cross-parameter space:

$$\Theta_{d,n}\;=\;\left\{(\alpha_{1},\ldots ,\alpha_{d},\mathrm{coeffs}\left(p_{1}^{\left(n\right)}\right),\ldots ,\mathrm{coeffs}\left(p_{d}^{\left(n\right)}\right))\right\}$$

that fully describes the approximant to *f*. Hence, the second Stone–Weierstrass application replaces infinite-dimensional basis functions $\gamma_{i}\in H_{k}$ with finite-dimensional polynomial surrogates $p_{i}^{\left(n\right)}$, thus turning the problem of learning *f* over Γ into an optimization over the finite-dimensional parameter set $\Theta_{d,n}$.

This two-step reduction maps the original optimization problem over the infinite-dimensional domain $\Gamma \subset H_{k}$ to an optimization over a finite-dimensional parameter space, while controlling the approximation error. The uniform error $\varepsilon_{2}$ in approximating each $\gamma_{i}$ propagates to the approximation of *f* in a way controlled by the Lipschitz constant of *f* with respect to γ, ensuring that the total error from both applications remains bounded when *d* and *n* are chosen large enough. So, by Lipschitz continuity of *f* on Γ with respect to ${∥\cdot ∥}_{H_{k}}$, with constant L>0, this perturbation changes the output of *f* by at most $L\;\varepsilon_{2}$. Consequently, the regret bound in the infinite-dimensional case satisfies

$$R_{\inf}^{T}\;\leq \;R_{\mathrm{finit}}^{T}\;+\;T\left(\varepsilon_{1}+L\;\varepsilon_{2}\right),$$

where $R_{T}^{\mathrm{finit}}$ is the cumulative-regret bound from the finite-basis case. By choosing the basis size *d* and polynomial degree *n* large enough, both $\varepsilon_{1}$ and $\varepsilon_{2}$ can be made arbitrarily small, so that the additive term $T(\varepsilon_{1}+L\;\varepsilon_{2})$ remains negligible compared to the leading $\sqrt{T}$-scale term in $R_{T}^{\inf}$. See Theorem 2 below for explicit assumptions and a sublinear cumulative-regret guarantee in the infinite-domain setting.

This reduction method explicitly connects the choice of basis to the covering number $N\left(\Gamma ,{∥\cdot ∥}_{H_{k}},\varepsilon \right)$, which determines the discretization size $|\Gamma_{\varepsilon }|$, where $\Gamma_{\varepsilon }=\left\{\gamma_{1},\ldots ,\gamma_{d}\right\}$ and

$$N\left(\Gamma ,{∥\cdot ∥}_{H_{k}},\varepsilon \right)=\min\left\{|\Gamma_{\varepsilon }|:\Gamma_{\varepsilon }\subset \Gamma \;\mathrm{is}\;\mathrm{an}\;\varepsilon \text{-cover}\;\mathrm{of}\;\Gamma ,\;\mathrm{for}\;{∥\cdot ∥}_{H_{k}}\right\}.$$

In the finite function space regret analysis of Theorem 1 (See Equation (A4)), the confidence parameter has the form $\beta^{T}=2\log(\frac{\pi^{2}T^{2}}{6\delta }\left|\Gamma |\right)$, which leads to following:

(65) $$\beta^{T}=2\log\left(\frac{\pi^{2}T^{2}}{6\delta }N\left(\Gamma ,{∥\cdot ∥}_{H_{k}},\varepsilon \right)\right)\leq 2\log\left(\frac{\pi^{2}T^{2}}{6\delta }\left|\Gamma_{\varepsilon }\right|\right);$$

as in practice, the set may come from a near-minimal cover so $d=|\Gamma_{\varepsilon }|\geq N\left(\Gamma ,{∥\cdot ∥}_{H_{k}},\varepsilon \right)$, i.e., the selected set forms a valid but not necessarily minimal ε-cover; typically bounded by a small factor (often at most logarithmic in *N* for standard greedy covering procedures on compact metric spaces (See Appendix D.2). This relationship in Equation (65) shows that $\beta^{T}$ grows logarithmically with *T* but also depends logarithmically on the covering number of Γ, so reducing *d* toward the minimal covering number directly leads to a smaller $\beta_{T}$ and, therefore, a tighter regret bound. Nevertheless, even for a valid, but not necessarily minimal, ε-cover, $|\Gamma_{\varepsilon }|$ is constant for a fixed ε, so $\beta_{T}\in O\left(\log T\right)$.

Considering all the elements mentioned above and following the same reasoning as in the proof of Theorem 1 (See Appendix A), the regret analysis for $R_{finit}^{T}$ can be extended to the infinite-dimensional domain $\Gamma \subset H_{k}$ using a discretization argument as follows. We first construct an $\epsilon_{T}$-cover $\Gamma_{\epsilon_{T}}\subset \Gamma$ in the $H_{k}$ norm, with size $|\Gamma_{\epsilon_{T}}|=d_{\epsilon_{T}}$, so that every γ∈Γ is within $\epsilon_{T}$ of some $\tilde{\gamma }\in \Gamma_{\epsilon_{T}}$. A union bound over all $\gamma \in \Gamma_{\epsilon_{T}}$ and all t≤T yields, with probability at least 1−δ, a uniform confidence interval of the form

$$|f\left(\gamma \right)-\mu^{t-1}\left(\gamma \right)|\leq \sqrt{\beta^{T}}\sigma^{t-1}\left(\gamma \right)+L\epsilon_{T},$$

where $\beta^{T}=2\log\;\left(\frac{T^{2}\pi^{2}}{6\delta }d_{\epsilon_{T}}\right)$ and *L* is the Lipschitz constant of *f* in the $H_{k}$ norm, ${∥\cdot ∥}_{H_{k}}$.

Applying this bound to both the optimal $\gamma^{*}$ and the chosen $\gamma^{t}$ in the round *t* shows that the instantaneous regret satisfies $r^{t}=f\left(\gamma^{*}\right)-f\left(\gamma^{t}\right)\leq 2\sqrt{\beta^{T}}\sigma^{t-1}\left(\gamma^{t}\right)+2L\epsilon_{T}$ (Proposition A2). Summing over t=1,…,T and using Cauchy–Schwarz together with the information gain bound $\sum_{t=1}^{T}\sigma_{t-1}^{2}\left(\gamma_{t}\right)\leq C_{1}I^{\tau }\left(f^{\tau };y^{\tau }\right)$ (Proposition A3), we obtain

(66) $$R_{\inf}^{T}=\sum_{t=1}^{T}\left[f\left(\gamma^{*}\right)-f\left(\gamma^{t}\right)\right]\leq 2\sqrt{C_{1}T\beta^{T}I^{T}\left(f^{T};y^{T}\right)}+2TL\epsilon_{T}.$$

Choosing $\epsilon_{T}=\frac{1}{T}$ makes the additive term $2TL\epsilon_{T}$ a constant, which can be upper-bounded by $\frac{\pi^{2}}{6}$, using $\sum_{t=1}^{\infty }\frac{1}{t^{2}}=\frac{\pi^{2}}{6}$. The final result is the high-probability bound.

(67) $$R_{\inf}^{T}\leq \sqrt{C_{1}T\beta^{T}I^{T}\left(f^{T};y^{T}\right)}+\frac{\pi^{2}}{6}.$$

**Theorem** **2.** *Let $\Gamma \subset H_{k}$ be compact in ${∥\cdot ∥}_{H_{k}}$, and let K: Γ×Γ→R be bounded and continuous. Suppose observations follow $y^{t}=f\left(\gamma^{t}\right)+\varepsilon^{t}$ with σ-sub-Gaussian noise $\varepsilon^{t}$, and f: Γ→R is a sample path of the centered functional GP GP(0,K) and is L-Lipschitz with respect to ${∥\cdot ∥}_{H_{k}}$. For analysis, fix any discretization schedule ${\left\{\varepsilon^{t}\right\}}_{t=1}^{T}$ and an $\varepsilon^{t}$-cover $\Gamma_{\varepsilon^{t}}\subset \Gamma$ with size $N(\Gamma ,{∥\cdot ∥}_{H_{k}},\varepsilon^{t})$. Run Algorithm 1 with GP-UCB and the exploration schedule*

$$\beta^{t}\;=\;2\log\;\left(\frac{\pi^{2}t^{2}\;N(\Gamma ,{∥\cdot ∥}_{H_{k}},\varepsilon^{t})}{6\;\delta }\right),\;\delta \in \left(0,1\right).$$

*Let $I_{T}$ denote the (maximum) information gain after T rounds. Then, with probability at least 1−δ,*

$$R_{inf}^{T}\;\leq \;\sqrt{\;C_{1}\;T\;\beta^{t}\;I_{T}\;}\;+\;2L\sum_{t=1}^{T}\varepsilon^{t},\;C_{1}\;=\;4\sigma^{2}\log\;\left(1+\sigma^{-2}\right).$$

*In particular, if $\sum_{t=1}^{\infty }\varepsilon^{t}<\infty$ (e.g., $\varepsilon^{t}=c/t^{2}$), then $R_{inf}^{T}=\tilde{O}\;\left(\sqrt{T\;I_{T}}\right)$ is sublinear; if moreover $I_{T}=o\left(T\right)$, then $R_{inf}^{T}=o\left(T\right)$.*

**Proof.**  Combine the finite- |Γ| GP-UCB bound with a union bound over the $\varepsilon^{t}$-covers $\Gamma_{\varepsilon^{t}}$ to obtain uniform confidence bands on $\Gamma_{\varepsilon^{t}}$, which yields

$$\beta^{t}=2\log(\pi^{2}t^{2}N(\Gamma ,{∥\cdot ∥}_{H_{k}},\varepsilon^{t})/\left(6\delta \right)).$$

Lipschitz continuity lifts these bands from $\Gamma_{\varepsilon^{t}}$ to all Γ, adding $2L\varepsilon^{t}$ to per-round regret; summing over *t* produces the stated bound. The two-step uniform-approximation argument (via discretization) recovers $R_{\inf}^{T}\leq R_{\mathrm{finit}}^{T}+T\left(\varepsilon_{1}+L\varepsilon_{2}\right)$, absorbed by $\sum_{t}\varepsilon^{t}$ when the schedule is summable. □

Therefore, schedule $\beta^{t}$ mirrors the finite-set case with |Γ| replaced by the covering number, and the bound extends Theorem 1 by adding a discretization penalty $2L\sum_{t}\varepsilon^{t}$. Contemplating the following assumptions for the infinite-domain analysis: (i) f∼GP(0,K) on Γ with *K* bounded and continuous. (ii) *f* is *L*-Lipschitz in ${∥\cdot ∥}_{H_{k}}$ on Γ. (iii) Noise $\varepsilon^{t}$ is σ-sub-Gaussian and Algorithm 1 uses $\beta^{t}$ as above. (iv) $\Gamma \subset H_{k}$ is compact and covers $\Gamma_{\varepsilon^{t}}$ exist with size $N(\Gamma ,{∥\cdot ∥}_{H_{k}},\varepsilon^{t})$. (v) The information gain $I_{T}$ is finite.

#### 4.4.4. Practical Implications of the Regret Bounds

The regret bounds for Stackelberg FCBO turn our theory into concrete guarantees for one-shot incentive design. They quantify, with high probability, how far the principal’s expected utility under the selected incentive can be from that of the best admissible incentive, before deployment. This matters in practice because the single-stage PAP requires a single commitment—there is no opportunity to “learn on the fly.”

The bounds certify a maximum difference in actual versus expected performance at the time of decision-making, offering an up-front measure of acceptable risk for using an incentive in important decisions (such as setting credit terms, granting subsidies, or allocating resources). Because the bound scales with differential information gain—that is, the knowledge added by each experiment—each offline evaluation reflects its informational value. This allows practitioners to prioritize experiments that reduce uncertainty the most, rather than running large, costly test batteries.

The bounds yield practical stopping rules. For example, stop when the acquisition-driven Upper Confidence Bound (a statistical estimate indicating the highest likely value) on the best candidate falls below a target gap (such as a minimum acceptable return on investment, ROI, or a maximum loss threshold). It also helps determine how many offline evaluations are needed to reach a desired near-optimality level, meaning how close the chosen candidate is to the optimal option.

In summary, the regret bounds provide actionable guarantees for offline policy selection: they quantify residual risk, guide the allocation of evaluation effort, and ensure that the final one-shot incentive is provably close to optimal under the given causal model and data.

### 4.5. On Extending CID to Multi-Follower Single-Stage Principal–Agent Problems

This subsection addresses the extension of the CID framework for single-stage principal–agent problems (SS–PAPs) to account for multiple followers. We illustrate this extension by presenting two contrasting CGMs for the base case of a PAP consisting of one principal and two followers. We use the notation $M_{L_{v}^{m}F_{u}^{n}}$, where the superscripts *m*, *n* represent the number of principal agents and follower agents in the CGM, respectively (we omit the superscript when it is equal to one, as in the canonical CGM $M_{L_{1}F_{1}}$), and the subscripts *v*, *u* the number of variables controlled by the principals, followers, respectively. To show how causal inference on canonical SS–PAPs can be extended to multi-follower SS–PAPs, we employ two representative models: the CGM $M_{L_{2}F_{1}^{2}}$ with individualized incentives and independent follower utility functions, and the CGM $M_{L_{1}F_{1}^{2}}$ with universal incentives and joint utility functions. For these CGMs, the endogenous variables are incentive function space variable Γ; follower actions $\Omega_{F}^{1},\Omega_{F}^{2}$; principal action $\Omega_{L_{1}},\Omega_{L_{2}}$ (individualized) or action $\Omega_{L}$ (universal); utilities variables $J_{F}^{1},J_{F}^{2},J_{L}$. We assume as before that the exogenous variables are mutually independent, mean-zero; and Gaussian when needed for analytic smoothing: $U_{A^{1}},U_{A^{2}}$ (follower action noises), $U_{L_{1}},U_{L_{2}}$ or $U_{L}$ (leader-action noises) and $U_{J_{F}^{1}},U_{J_{F}^{2}},U_{J_{L}}$ (utility noises). In this subsection, for clarity in the notation, we sometimes use $a^{1}$, $a^{2}$ or $a^{i}$, $a^{-i}$ for the followers actions $\omega_{F}^{1}$ and $\omega_{F}^{2}$, and $A_{1}$, $A_{2}$ for $\Omega_{F}^{1}$, $\Omega_{F}^{2}$. As has been consistently acknowledged in this research, we assume the principal’s perceptive; i.e., the principal does not have knowledge about the followers utilities nor their best-response maps.

#### 4.5.1. CGMs with Individualized Incentives and Independent Follower Utilities

In $M_{L_{2}F_{1}^{2}}$, the principal has two decision variables $\Omega_{L_{1}}$ and $\Omega_{L_{2}}$, i.e., it must decides on two distinct incentive functions $\gamma_{1}\in \Gamma$ and $\gamma_{2}\in \Gamma$, one for each follower. This type of CGM for SS–PAPs with multiple followers allows the selection of distinct incentives for different followers, which is useful in multi-agent systems where it is important to break symmetries between followers. Furthermore, in the $M_{L_{2}F_{1}^{2}}$ CGM, the utility function of each follower does not depend on the decisions of the other followers, i.e., there is no interference between the follower utilities: $J_{F}^{i}$ depends only on its own channel (though principal utility $J_{L}$ may couple channels). $\Gamma_{1}\;\to \;\Omega_{F}^{1}$ and $\Gamma_{2}\;\to \;\Omega_{F}^{2}$ via best responses (68), as shown in the structural equations F given below:

(68)ΩF1=BR1(Γ1,UA1),ΩF2=BR2(Γ2,UA2),(69)ΩL1=Γ1(ΩF1)+UL1,ΩL2=Γ2(ΩF2)+UL2,(70)JF1=gF1(ΩL1,ΩF1)+UJF1,JF2=gF2(ΩL2,ΩF2)+UJF2,(71)JL=gL(ΩL1,ΩL2,ΩF1,ΩF2)+UJL.Γ1=γ1;Γ2=γ2

Therefore, choosing policies $\Gamma_{1}=\gamma_{1},\Gamma_{2}=\gamma_{2}$, the principal wants to estimate the causal estimand $V\left(\gamma_{1},\gamma_{2}\right)=E\;\left[J_{L}\;|\;\mathrm{do}\left(\Gamma_{1}=\gamma_{1},\Gamma_{2}=\gamma_{2}\right)\right]$ and targets incentive policies $\gamma_{1}^{*}$ and $\gamma_{2}^{*}$ such that

(72) $$\gamma_{1}^{*},\gamma_{2}^{*}\in \underset{\gamma_{1}\in \Gamma_{1},\gamma_{2}\in \Gamma_{2}}{\arg\max}\;E\left[J_{L}|do\left(\Gamma_{1}=\gamma_{1},\Gamma_{2}=\gamma_{2}\right)\right].$$

Using the *g*-formula, the identification formula for $V(\gamma_{1},\gamma_{2})$ admits the familiar nested expectation form:

(73)V(γ1,γ2)=∫∫EUL1,UL2gLγ1(a1)+UL1,γ2(a2)+UL2,a1,a2︸innerexpectation(Gaussiansmoothing)(74)×p(a1∣Γ1=γ1)p(a2∣Γ2=γ2)da1da2,

#### 4.5.2. CGMs with Universal Incentive and Joint Follower Utilities

In contrast, in $M_{L_{1}F_{1}^{2}}$, incentives are said to be universal, that is, the incentive function γ is applied equally to all followers, and here the utility function of each follower depends on the decisions of the other followers. The structural equations F for $M_{L_{1}F_{1}^{2}}$ are given in Equations (76)–(79).

(75)Γ=γ(76)(ΩF1,ΩF2)=Φ(Γ,UA1,UA2)(Jointbest-responseequilibriummap),(77)ΩL=Γ(ΩF1,ΩF2)+UL,(78)JFi=gFi(ΩL,ΩF1,ΩF2)+UJFi,i∈{1,2},(79)JL=gL(ΩL,ΩF1,ΩF2)+UJL.

The direct causal relations from arrows $\Gamma \;\to \;(\Omega_{F}^{1},\Omega_{F}^{2})$ are via the joint best-response equilibrium map Φ; as shown in the structural Equation (76). One can write $\Omega_{F}^{1}={\mathrm{BR}}_{1}\left(\Gamma_{1},U_{A^{1}}\right)$, $\Omega_{F}^{2}={\mathrm{BR}}_{2}\left(\Gamma_{2},U_{A^{2}}\right)$, and define Φ as the fixed-point solver. That is, each follower *i* has payoff $J_{F}^{i}\left(a^{i},a^{-i};\gamma \right)$ with the best-response correspondence:

$${\mathrm{BR}}_{i}\left(\gamma ,a^{-i}\right)\;\in \;\arg\max_{a^{i}\in A_{i}}u_{i}\left(a^{i},a^{-i};\gamma \right),\;i\in \left\{1,2\right\},$$

and the joint best-response correspondence is

$$B_{\gamma }\left(a^{1},a^{2}\right)\;=\;{\mathrm{BR}}_{1}\left(\gamma ,a^{2}\right)\times {\mathrm{BR}}_{2}\left(\gamma ,a^{1}\right)\;\subseteq \;A_{1}\times A_{2},$$

for compact action sets $A_{i}\subset R^{d_{i}}$. So, $B_{\gamma }\left(a^{1},a^{2}\right)$ takes an action profile $\left(a^{1},a^{2}\right)$ as input and returns the set of all possible joint actions where each player is best responding to the input action of the other player. A Nash equilibrium under γ is then any fixed point $\left(a^{1★},a^{2★}\right)\in B_{\gamma }\left(a^{1★},a^{2★}\right)$, as $a^{1★}\in {\mathrm{BR}}_{1}\left(\gamma ,a^{2★}\right)$, i.e., follower 1’s action is a best response to follower 2’s action; and $a^{2★}\in {\mathrm{BR}}_{1}\left(\gamma ,a^{1★}\right)$, i.e., follower 2’s action is a best response to follower 1’s action. However, in scenarios where the followers game presents multiple Nash Equilibria (NE), the conventional method of defining NE as a fixed point fails to provide clarity on the actual outcome that players will select, resulting in a model that lacks predictive capability. Therefore, it is important to consider some additional assumptions for this joint best-response equilibrium map Φ, such as the existence of the equilibrium and the specification of an equilibrium selection rule, where the goal is to move from the Nash equilibrium correspondence (a set of possible outcomes) to a unique joint action law (a single predicted outcome).

Under $M_{L_{1}F_{1}^{2}}$ the principal wants to estimate the causal estimand $V\left(\gamma \right)=E\;\left[J_{L}\;|\;\mathrm{do}(\Gamma =\gamma \right]$ and targets the incentive function $\gamma^{*}$ such that:

(80) $$\gamma^{*}\in \underset{\gamma \in \Gamma }{\arg\max}\;E\left[J_{L}|do\left(\Gamma =\gamma \right)\right].$$

By the *g*-formula, the identification for V(γ) also admits the nested expectation form:

(81) $$\begin{array}{cc} V(\gamma ) & =\;\int \int \;\underset{\mathrm{inner}\;\mathrm{expectation}\;(\mathrm{Gaussian}\;\mathrm{smoothing})}{\underset{︸}{E_{U_{L}}\;\left[g_{L}\left(\gamma \left(a^{1},a^{2}\right)+U_{L},\;a^{1},\;a^{2}\right)\right]}}\;p\left(a^{1},a^{2}|\Gamma =\gamma \right)\;da^{1}\;da^{2}. \end{array}$$

In the identification formula for V(γ), we can observe the importance of having a joint best-response equilibrium map Φ with an equilibrium selection rule. In multiple equilibria cases, $p(a^{1},a^{2}|\Gamma =\gamma )$ becomes non-unique, ambiguous, and very hard to estimate from data; so it is important to impose or learn an equilibrium selection rule that makes the joint action law unique or as unambiguous as possible.

## 5. Discussion

This work formalizes the Causal Incentive Design (CID) framework for the canonical single-stage principal–agent problem (SS–PAP) by treating incentives as interventions in a causal graphical model (CGM). The causal target $V\left(\gamma \right)=E[J_{L}|do\left(\Gamma =\gamma \right)]$ is the expected value of the principal’s utility variable $J_{L}$ under a specified policy intervention Γ=γ in the post-intervention distribution $p\left(J_{L}|do\left(\Gamma =\gamma \right)\right)$. The construction of an estimand by the identification of V(γ) is via the g-formula, and the selection of a single deployment incentive policy is through a Functional Bayesian Optimization algorithm. The process pipeline on CID for SS–PAPs is explicitly offline: historical data records from the system are mapped into estimates of V(γ), which are then optimized *prior* to a one-shot commitment. This connection between causal identification and sample-efficient search yields high-probability regret guarantees for the offline selection step.

A central contribution is the anatomy of the estimand V(γ) as a nested expectation with two interpretable layers: (i) an inner Gaussian smoothing of the outcome regression around the principal’s induced action, and (ii) an outer averaging with respect to the follower’s induced action law p(M∣Γ=γ); which extends to multi-follower setting. The inner layer admits quadrature-based evaluation (e.g., Gauss–Hermite), while the outer layer is handled by a policy-local reweighting scheme that respects positivity and interpretability. In the affine credit-market specialization (Section 3.3.1), this decomposition collapses to a closed form, clarifying how pricing slope and fees interact with induced borrowing behavior.

The CID framework demonstrate a recent advance in causal reasoning grounded in causal graphical models (CGMs): policies are formalized as functional interventions on a policy node, policy value is represented as an interventional or counterfactual query, and identification and estimation are performed directly on the CGM using observational logs. Additionally, the single-stage principal–agent model examined here introduces a novel application domain for CGMs in incentive design, complementing established applications in biology, medicine, and economics, and facilitating further exploration of multi-agent scenarios.

The remainder of the Discussion is structured as follows. Section 5.1 revisits the Stackelberg FCBO procedure and interprets the information–gain regret bounds as causal decision guarantees for one-shot policy selection in single-stage PAPs. Section 5.2 reviews the primary modeling, identification, and learning assumptions of the CID framework, including the CGM structure, positivity/overlap, the additive-noise model, and the uniform sub-Gaussian noise envelope used in the regret analysis. Section 5.3 discusses the practical scope of the current single-stage, offline analysis, emphasizing limitations related to policy-local support, potential model misspecification such as heavy-tailed noise or unmodeled confounding, and robustness considerations, as well as the importance of effective-sample-size diagnostics. Section 5.4 analyzes the computational demands of the CID pipeline and the FCBO algorithm, specifying how time and memory requirements scale with the number of logged units, the number of policy evaluations, and GP updates, and indicating regimes in which value estimation or surrogate modeling is the dominant computational cost. Section 5.5 describes extensions of the CID framework and nested estimand beyond the single-follower case, considering individualized versus joint incentives and the impact of interactions among multiple followers on the CGM, identification, and the design of learning algorithms.

### 5.1. Offline Functional BO and Causal Decision Guarantees

The Stackelberg FCBO algorithm models γ↦V(γ) as a black-box functional objective on a Reproducing Kernel Hilbert Space (RKHS) and uses a functional GP surrogate with a GP-UCB-style acquisition functional. The resulting cumulative-regret bounds for the Stackelberg FCBO algorithm scale with $\sqrt{T\;\beta^{t}\;I_{T}}$, where $I_{T}$ is an information gain term. This offers concrete guidance on evaluation budgets (sampling costs), stopping rules (such as UCB gaps), and the exploration schedule prior to the one-shot deployment; i.e., determining when enough data have been collected to make a final decision.

In contrast to online-learning methods such as bandits and reinforcement learning over actions, CID for SS–PAPs is distinctly one-shot and offline, utilizing intrinsic causal structure in PAPs and causal reasoning to assess policies without the need for deployment. In relation to causal BO methods, we argue that interventions do(Γ=γ) in the function-valued random variable Γ, which involve functional (conditional) interventions $do\left(\Omega_{L}=\gamma \left(\omega_{L}\right)\right)$, since

$$E\left[J_{L}|do\left(\Gamma =\gamma \right)\right]\approx E\left[J_{L}|do\left(\Gamma =\gamma \right),do\left(\Omega_{L}=\gamma \left(\omega_{L}\right)\right)\right],$$

are the right abstraction for causal reasoning about incentive functions in PAPs. In $M_{L_{1}F_{1}}$, the node Γ explicitly represents the incentive policy and is connected to both $\Omega_{L}$ and $\Omega_{F}$. These connections $\Gamma \to \Omega_{L}$ and $\Gamma \to \Omega_{F}$, together with $\Omega_{F}\to \Omega_{L}$, $\Omega_{L}\to J_{L}$, and $\Omega_{F}\to J_{L}$, constitute the minimal policy-aware motif that captures how incentives γ∈Γ shape the leader’s realized decision $\Omega_{L}$ and the follower’s decision $\Omega_{F}$, to produce the principal’s utility variable $J_{L}$ in the system. In structural terms, the leader’s action is generated by the policy applied to the follower context, $\Omega_{L}=\gamma \left(\Omega_{F}\right)$, while the adoption of a policy also reshapes the distribution of follower variables through $\Gamma \to \Omega_{F}$.

This inverse Stackelberg-aware approach makes the hierarchical semantics explicit: the dependence between principal and follower decisions is encoded both by $\Omega_{F}\to \Omega_{L}$ (the policy takes follower context as input) and through behavior modifications driven by incentive policies $\Gamma \to \Omega_{L}$ and $\Gamma \to \Omega_{F}$ (the policy governs how actions are produced and how follower behavior is distributed under intervention). Moreover, the follower decision $\Omega_{F}$ acts as a mediator *M* in the causal path from Γ to $J_{L}$ in the principal’s perspective of $M_{L_{1}F_{1}}$, in agreement with the intrinsic semantics of PAPs.

The CID framework for SS–PAPs illustrates the fundamental difference between considering the causal relations between the input variables ( Γ, $\Omega_{F}$ in SS–PAPs ) and not considering them in an optimization problem. This variation is the central motivation in the causal decision-making framework of Causal Bayesian Optimization (CBO) [24] over classical Bayesian optimization. In this sense, the CID framework for SS–PAPs exhibits PAPs, inverse Stackelberg games, and bilevel optimization problems, as a fundamental family of problems that elucidates this important distinctness (between BO and CBO), given that the hierarchical semantics of an inverse Stackelberg game rely on the causal relationships $\Gamma \to \Omega_{L}$, $\Gamma \to \Omega_{F}$ and $\Omega_{F}\to \Omega_{L}$. Disregarding the causal relationships between these three variables Γ, $\Omega_{F}$, and $\Omega_{L}$ merely distorts the semantics of the functional optimization problem that solves a PAP.

### 5.2. Assumptions

The identification and estimation of CID are based and rely on the following assumptions: (i) *Consistency*: the recorded outcome equals the counterfactual under the realized policy. (ii) *Causal sufficiency*: for variables $\left\{\Gamma ,\Omega_{L},\Omega_{F},J_{L},J_{F}\right\}$ in the generative CGM $M_{L_{1}F_{1}}$; (iii) *Black-box follower’s best-response*: we work over the principal’s perspective where the observed variables are $\left\{\Gamma ,\Omega_{F},\Omega_{L},J_{L}\right\}$ and the follower’s best-response is considered a black-box function. (iv) *Positivity (overlap)*: in a policy-local sense, we assume that there exists a neighborhood $N_{\rho }\left(\gamma \right)$ such that $p(\Omega_{F}|\Gamma \in N_{\rho }\left(\gamma \right))>0$ wherever the estimator needs support to approximate $p(\Omega_{F}|\Gamma =\gamma )$. (v) *Policy compliance*: The action implemented by the principal is consistent with the distribution of $\Omega_{L}=\gamma \left(\Omega_{F}\right)+U_{L}$ in practical deployment, as it is presupposed. Otherwise, if this alignment is not achieved, the action do(Γ=γ) and the behavior observed in practice will differ significantly. (vi) *Additive, zero-mean Gaussian noise SCM*: featuring mutually independent exogenous terms; in the leader’s action, in particular, allowing inner Gaussian smoothing on the identification formula. We also assume i.i.d. logged units and no interference across units, i.e., the Stable Unit Treatment Value Assumption (SUTVA). Violations or weak forms of these conditions, such as latent confounding, measurement error, or insufficient policy-local overlap, can bias V(γ) and compromise the calibration of uncertainty. (vii) *Noise envelope for regret analysis:* While the outer estimator is heteroskedastic across γ, we assume a uniform sub-Gaussian envelope on $\varepsilon_{t}=\hat{V}\left(\gamma_{t}\right)-V\left(\gamma_{t}\right)$ over the proposal support. This implies a diagonal noise model in Section 4.4.1 with $\Sigma_{T}⪯\sigma_{*}^{2}I$, under which the stated GP-UCB bounds hold (see [8,29]).

### 5.3. Scope and Limitations

The present CID analysis is *single-stage and offline*: it commits once to a policy and cannot adapt after deployment. Dynamics (carry-over effects), learning-by-doing, and path-dependent constraints are beyond the scope of this paper. The follower mechanism is treated as a black box (we observe $\Omega_{F},\Omega_{L},J_{L}$, but not the follower utilities); this pushes strategic dependence into the observed mediator law and increases sensitivity to support gaps in $\Omega_{F}$ near the target policy γ. So, in the absence of extensive historical policy support, the sensitivity in the outer layer increases. The additive Gaussian assumption simplifies the inner layer but may under-represent heavy tails or heteroskedasticity; when noise departs from Gaussian, it can lead to inaccuracies in inner smoothing and variance estimates.

For diagnostics and protection against weak overlap: (i) Monitor weight distributions, to look for outlier weights pointing to weak overlap and high variance. (ii) Monitor the effective sample size (ESS), which measures the effective number of independent samples you have after applying the weights.

$$\mathrm{ESS}\left(\gamma ;h\right)\;:=\;\frac{{\left(\sum_{i=1}^{N}w_{i}\left(\gamma ;h\right)\right)}^{2}}{\sum_{i=1}^{N}w_{i}{\left(\gamma ;h\right)}^{2}}.$$

A low ESS relative to the total sample size (*N*) indicates that a few heavily weighted units dominate the estimation, signaling a high variance problem. (iii) Monitor sensitivity to the policy-space bandwidth, by re-running the evaluation using different values for this bandwidth parameter *h*. If the value of the new policy changes significantly when you slightly adjust the bandwidth, the estimate is unstable and highly dependent on a tuning parameter, which reduces confidence in the result. (iv) Reduce overfitting bias in the outer layer employing sample-splitting, cross-fitting, and orthogonalization.

In practice, Algorithm 1 can constrain candidate policies to a trust region $S_{\mathrm{support}}$ (see Section 3.3.4 for the definition of $S_{\mathrm{support}}$), adapt the bandwidth *h* to balance bias–variance and/or inflate posterior uncertainty as γ approaches the boundary of $S_{\mathrm{support}}$. Crucially, kernel reweighting does not create support: if γ is far from the historical policies, ${\hat{P}}_{\gamma }^{\left(h\right)}$ collapses, and $\hat{V}\left(\gamma \right)$ becomes high-variance (and possibly biased). Therefore, the practical search space of Algorithm 1 is data-dependent and effectively limited to neighborhoods of previously observed policies. Because the outer estimator relies on policy-local overlap, we can incorporate a policy-support score s(γ):=ESS(γ;h)/N into the selection. Concretely, we can either constrain GP-UCB to $\gamma \in S_{\mathrm{support}}\left(h,\tau ,w_{\max}\right)$ (hard trust region) or use a soft penalty ${\tilde{A}}_{t}\left(\gamma \right)=A_{t}\left(\gamma \right)\cdot \varphi \left(s\left(\gamma \right)\right)$, where $A_{t}\left(\gamma \right)$ is the acquisition function, with φ(s)↓0 as s↓0, i.e., this penalty function must approach zero as the policy-support score s(γ) approaches zero. Conversely, when s(γ) is high (close to 1), φ(s) should be close to 1 to minimally impact the acquisition function $A_{t}\left(\gamma \right)$. This prevents proposals in regions where p(M∣Γ=γ) cannot be estimated from logs and makes the practical search space explicit and data-dependent.

### 5.4. Time–Space Complexity over a Full FBO Run

Estimating V(γ) once via the semi-parametric identification formula (assuming additive, zero-mean Gaussian noise) costs $O\;\left(N\;(P+C_{\gamma }+Q_{\mathrm{eff}}\;C_{\mu })\right)$, under policy-local reweighting over *N* recorded tuples, and where *P* is the policy-space representation size, $C_{\gamma }$ is the cost to compute $\gamma (\Omega_{F})$ once, $Q_{\mathrm{eff}}$ is the inner integration budget (e.g., Gauss–Hermite nodes or MC points), and $C_{\mu }$ is the cost to evaluate the fitted outcome regression once. Across a BO horizon of *T* iterations (one evaluation per step), the total cost to produce these off-line evaluations is $O\;\left(T\;N\;(P+C_{\gamma }+Q_{\mathrm{eff}}\;C_{\mu })\right)$. With local reweighting over only the top L≪N nearest policies, this becomes $O\;\left(T\;(P\log N+L\;\left(P+C_{\gamma }+Q_{\mathrm{eff}}\;C_{\mu }\right))\right)$. The one-time training of the outcome model contributes an upfront $C_{\mathrm{train}}^{\mu }$ that is then amortized. The memory requirement for the estimator is O(N) (or O(L) when local weighting is applied), in addition to O(NP) if the policy embeddings are stored in cache. The quadrature nodes are $O(Q_{\mathrm{eff}})$ and can be considered negligible.

The implementation of an exact functional Gaussian Process surrogate with incremental updates throughout the execution adds $O\;\left(\left(1+B\right)\;T^{3}+\left(1+B\right)\;T^{2}\;P\right)$ for Cholesky updates and acquisition scoring of *B* candidates per iteration. Since, across the full horizon *T*, kernel updates are $\sum_{t=1}^{T}O\left(N_{t}P\right)=O\left(T^{2}P\right)$; Cholesky factorizations are $\sum_{t=1}^{T}O\left(N_{t}^{2}\right)=O\left(T^{3}\right)$; and total UCB functional acquisition scoring work (each needs mean and variance) $\sum_{t=1}^{T}O\left(B\;(N_{t}P+N_{t}^{2})\right)=O\left(B\;(T^{2}P+T^{3})\right)$, where $N_{t}$ is the number of observed policy evaluations at iteration *t* (so $N_{t}=t$). Merging the estimation cost and exact GP updates plus functional acquisition work cost, the total end-to-end time is

$$\mathrm{Total}\;\mathrm{time}\;\approx \;C_{\mathrm{train}}^{\mu }\;+\;\underset{\mathrm{For}\;\mathrm{all}\;\hat{V}\left(\gamma \right)\;\mathrm{evaluations}}{\underset{︸}{O\left(T\;N\;(P+C_{\gamma }+Q_{\mathrm{eff}}C_{\mu })\right)}}\;+\;\underset{\mathrm{For}\;\mathrm{the}\;\mathrm{exact}\;\mathrm{functional}\;\mathrm{GP}}{\underset{︸}{O\;\left(\left(1+B\right)\;T^{3}+\left(1+B\right)\;T^{2}P\right)}}.$$

Replace *N* by *L* and add TPlogN under local weighting. Comparing the estimation cost $T\;N\;(P+C_{\gamma }+Q_{\mathrm{eff}}\;C_{\mu })$ of V(γ) with the dominant cubic GP term $\left(1+B\right)\;T^{3}$, we can establish that GP dominates when

$$T\gg \sqrt{N\;\left(P+C_{\gamma }+Q_{\mathrm{eff}}C_{\mu }\right)/\left(1+B\right)},$$

while the estimation cost of V(γ) dominates below that scale. In terms of space, the exact GP stores dense Gram/Cholesky factors of size T×T, i.e., $O(T^{2})$.

### 5.5. Toward Multi-Follower Settings

We outline a multi-follower generalization of CID that preserves the CGM semantics of the canonical case. Let followers be indexed by i∈[n] with contexts $\Omega_{F}^{\left(i\right)}$, and let the leader’s (possibly vector-valued) action be $\Omega_{L}$. The policy node Γ represents the incentive rule(s) chosen by the principal and, in the policy-augmented CGM, carries arrows $\Gamma \;\to \;\Omega_{L}$ and $\Gamma \;\to \;\Omega_{F}^{\left(i\right)}$ for all *i*. As in the single-follower case, contexts feed into the leader’s realized action via $\Omega_{F}^{\left(i\right)}\;\to \;\Omega_{L}$, but now the post-intervention law involves the joint mediator $M=(\Omega_{F}^{\left(1\right)},\ldots ,\Omega_{F}^{\left(n\right)})$, since both $\Omega_{L}$ and $\left\{\Omega_{F}^{\left(i\right)}\right\}$ affect the principal’s payoff $J_{L}$. Two instructive extensions clarify how identification and learning must adapt beyond the canonical case: (i) the regime IIIU: individualized incentives with conditionally independent follower utilities (factorized mediator law), and (ii) the regime UIJU: universal incentives with joint follower utilities (coupled actions via a joint best-response map).

In the IIIU regime, the principal deploys a collection of incentive functions (e.g., one per channel, segment, or product), and the followers’ utilities are *conditionally independent* given their own contexts and the relevant part of the policy. Graphically, the mediator law factorizes under interventions as follows:

(82) $$p\;\left(M\;|\;do\left(\{\Gamma^{\left(i\right)}=\gamma^{\left(i\right)}\}\right)\right)\;=\;\prod_{i=1}^{n}p\;\left(\Omega_{F}^{\left(i\right)}\;|\;do\left(\Gamma^{\left(i\right)}=\gamma^{\left(i\right)}\right)\right).$$

Estimation and optimization inherit a separable structure: outer-layer reweighting and inner smoothing decompose over *i*, enabling parallel computation and straightforward uncertainty aggregation. Surrogate modeling should mirror this factorization (e.g., with parallel GPs, modeling the functional objective as a sum or collection of independent functional GPs or using block-diagonal kernels over policy components, where the overall kernel is constructed by combining separate, independent kernels for each component), and identifiability reduces to policy-local overlap for each channel. This setting suits applications where incentives are personalized or targeted and cross-follower externalities are negligible.

In the UIJU regime, a single policy γ applies to all followers whose actions are strategically coupled; so, we use a joint best-response map $A\;=\;\Phi \left(\gamma ,M,U_{F}\right)$, with exogenous terms $U_{F}$, where we use *A* to denote the actions carried out by followers $\left\{\omega_{F}^{\left(1\right)},\ldots ,\omega_{F}^{\left(n\right)}\right\}$. The identification requires an *equilibrium-selection* rule to make the post-intervention law; i.e., $p\;\left(A\;|\;M,\;do(\Gamma =\gamma )\right)$ is well defined through a selection mapping *S*: (γ,M)↦A. Three practical approaches are (i) *Uniqueness*: Assume that do(Γ=γ) almost surely results in a unique equilibrium outcome *A*. (ii) *Parametric selection*: Encode tie-breaking or stability criteria in *S* and learn its parameters from observed play. (iii) *Stochastic selection*: Models the choice among equilibria as a stochastic process, where each equilibrium is chosen with a specific probability.

Surrogate modeling for the UIJU regime should reflect cross-follower dependence (e.g., using multi-task GPs with coregionalization and structured kernels over policy components), and outer-layer estimation must use joint reweighting over *M* (and, if modeled, *A*) rather than product weights. Positivity must hold in a joint sense: support near γ is needed for the relevant regions of the (M,A)-space, a stricter requirement than in the individualized case.

## 6. Conclusions

This study introduces a principled, offline framework for single-stage incentive design using causal inference, grounded in causal graphical models (CGMs) and information-theoretic analysis. The single-stage principal–agent problem (SS–PAP) is formalized as a CGM, where the principal’s incentive rule is modeled as a functional intervention on a policy node Γ, the follower’s action serves as an explicit causal mediator, and the principal’s payoff $J_{L}$ is the outcome. The causal target estimand is defined as the principal’s expected utility under an incentive policy intervention, $V\left(\gamma \right)=E[J_{L}|do\left(\Gamma =\gamma \right)]$, which quantifies how a committed incentive causally influences the follower’s behavior and the principal’s payoff under private information.

For the identification of the estimand, a semi-parametric expression for V(γ) is derived using the g-formula, which decomposes into an inner Gaussian smoothing over noise and an outer expectation with respect to the post-intervention distribution of the mediator. Building on this structure, a two-layer estimator is proposed for offline logs: the inner layer computes a Gaussian expectation, implemented numerically via Gauss–Hermite quadrature, while the outer layer approximates the interventional mediator law using policy-local kernel reweighting with effective-sample-size and weight-cap diagnostics. This design explicitly addresses the bias–variance trade-off and ensures that off-policy evaluation maintains overlap in policy space.

For policy selection, the value functional γ↦V(γ) is embedded in a functional Gaussian process surrogate over a Reproducing Kernel Hilbert Space (RKHS) of admissible incentives, and a support-aware GP-UCB search is performed. The resulting regret bounds are characterized in terms of differential information gain, directly connecting the exploration–exploitation trade-off to information-theoretic quantities under a uniform sub-Gaussian envelope for estimator noise. Collectively, these components establish a unified pipeline for causal reasoning and optimal intervention design in single-stage principal–agent settings using only offline observational logs.

The proposed offline approach is particularly suitable in scenarios where adaptive experimentation is infeasible, ethically constrained, or operationally costly. For instance, this applies when contracts, tariffs, or credit terms must be fixed prior to deployment and cannot be iteratively adjusted. Within this regime, the analysis clarifies the conditions under which causal identification is possible, the extent to which extrapolation in policy space is feasible while maintaining overlap, and methods for quantifying uncertainty through effective sample size and information gain.

The CID framework extends beyond the canonical single-follower case. In a multi-follower generalization, the nested form of the estimand is preserved, but a strong dependence emerges based on the interaction of follower utilities. In regimes with individualized incentives and conditionally independent utilities, identification, estimation, and surrogate modeling decompose across followers, enabling parallelization and simpler diagnostics. Conversely, in regimes with universal incentives and jointly coupled utilities, identification depends on equilibrium-selection assumptions, and outer-layer estimation must address joint mediator laws and stricter positivity requirements. These extensions demonstrate both the robustness of the two-layer CID structure and the additional challenges encountered in more complex multi-agent systems.

Overall, the presented CID framework advances the field of causal graphical models and their applications by integrating a CGM-based representation of principal–agent interactions with explicit causal reasoning about policy interventions and information-theoretic guarantees for offline policy search. This framework establishes a foundational link between causal inference and policy search in principal–agent problems, supporting reliable single-stage incentive design in complex systems and providing a basis for future research involving empirical studies, richer policy classes, and more complex multi-follower environments.

### Future Work

The proposed approach enables the integration of causal reasoning into incentive design, especially for canonical single-stage PAPs. It also reveals opportunities to strengthen robustness in causal inference estimation, improve computational scalability, validate the framework under realistic constraints, and expand to multi-follower and multi-stage PAPs. Future work will address these limitations and explore extensions to make CID dependable at scale and deployable in practice.

Beyond the scope of this work, an important future extension is a robust identification toolkit that tolerates limited policy-local overlap in data logs and mild unmeasured confounding. Specifically, we plan to incorporate partial-identification estimation bounds, i.e., reporting intervals for the causal target estimand V(γ) rather than a single point estimation, with techniques based on [30] tailored to policy interventions, or proximal/negative-control strategies when credible proxy variables are available. In addition to targeted sensitivity analyses using weight clipping and bandwidth sweeps with effective sample size diagnostics. By addressing this limitation, the practical utility of CID improves in data regimes where perfect overlap and strict sufficiency are unrealistic.

Other aspects for strengthening robustness involve exploring beyond additive-Gaussian noise and augmenting policy-local reweighting with structural conditional density models to learn the mediator law given an incentive policy p(M∣Γ). Regarding the first, we plan to replace the inner Gaussian smoothing with robust or heavy-tailed quadrature methods, such as Student-t distributions or mixture models, which can better account for outliers and extreme data points. In addition, to explore heteroskedastic noise models and variance-adaptive smoothing, to propagate these refinements into uncertainty quantification. Regarding conditional density models for p(M∣Γ), the plan is to utilize conditional normalizing flows or score-based estimators while preserving the identification logic and benchmarking bias–variance trade-offs. These improvements are essential to reduce bias in heavy-tailed regimes and to maintain calibrated uncertainty when noise scale depends on context.

The forthcoming work will involve the complete extension of CID to multi-follower over the distinct configurations. This will require adaptations to surrogate modeling tailored to specific regimes and adjusting the regret bounds. Many systems are situated between regimes: individualized incentives with conditionally independent follower utilities and universal incentives with joint follower utilities. A pragmatic approach is to adopt clustered dependence: partition followers into groups with strong in-group coupling and weak cross-group effects. The CGM should then mix block-factorized mediator laws with group-level equilibrium selection. For a large amount of followers, exchangeability and mean-field approximations can effectively reduce dimensionality. These techniques summarize follower interactions through aggregate statistics (such as empirical means or occupancy measures), which can be used as input to the system’s laws and particularly on the joint best-response map, yielding scalable identification and surrogate models with kernels over aggregates. This implies measuring similarity between two policies based on the similarity of their predicted aggregate statistics, making the optimization process efficient for massive populations.

Beyond the existing framework, a promising direction is constraint-aware CID. The core motivation is that incentive policies deployed in real-world settings (such as credit, pricing, or subsidies) must reliably satisfy constraints, and not just on average. This means mitigating the risk of rare and catastrophic failures. Essential constraints include safety (avoiding dangerous or unintended side effects), budget (ensuring costs remain within defined limits), and fairness (ensuring similar treatment or impact across groups, minimizing disparities in approval rates or resource allocation). The planned efforts on this matter involve implementing three interconnected components in the search process for policy interventions: (i) constrained acquisitions that enforce high-probability feasibility; (ii) risk-sensitive objectives and chance constraints; and (iii) fairness controls. The analysis of this extension must delineate clear Pareto trade-offs, enabling decision-makers to transparently assess trade-offs between maximizing utility and satisfying constraints such as safety, fairness, and budget.

A paramount direction is to generalize from the single-stage setting to multi-stage environments, where incentives are applied over time. Specifically, we plan to formalize a dynamic CGM with history-dependent policies Π, identify the causal target V(Π) with a sequential (stage-wise) analogue of our two-layer estimator, and lift the optimization to a Dynamic Functional Causal Bayesian Optimization algorithm that accounts for temporal causal relations and stage-wise uncertainty. This expansion is essential to capture learning-by-doing, carryover effects, and path-dependent constraints—features central to many real-world deployments.

Finally, translating these theoretical gains into robust real-world value is a key long-term goal. This involves curated benchmarks (credit pricing, platform subsidies, and market mechanisms), rigorous baselines (contextual bandits with off-policy evaluation (OPE), policy-gradient RL with OPE, and non-causal BO), and comprehensive reporting of offline policy regret, uncertainty coverage, and constraint satisfaction—with open, reproducible artifacts. Future work will include detailed simulations and case studies to demonstrate (a) calibration of the two-layer estimator under policy dispersion and overlap constraints; (b) separation of outer policy-local reweighting and inner Gaussian smoothing through ablation studies; and (c) performance of end-to-end Functional Causal Bayesian Optimization (FCBO) with support-aware acquisition across representative policy classes.

Additionally, it is essential to monitor run-time and memory usage as policy classes and evaluation budgets expand. The primary bottleneck in Bayesian Optimization (BO) is the Gaussian Process (GP) surrogate model, which often has cubic time complexity over the BO horizon. This can be overcome by replacing costly exact computations with approximations, such as random-feature surrogates chosen in the policy space, and by coordinating multi-fidelity evaluations of V(γ). That is, using “cheap samples” that can be sampled quickly at a low computational cost in the inner layer of the identification formula to gain a coarse understanding of the policy’s value, and reserve the expensive, high-accuracy estimations (like high-fidelity quadrature for integrating probabilities) for only the most promising policies. This is essential for validating scalability in resource-constrained environments and for enabling larger, more expressive policy classes without sacrificing regret guarantees.

Taken together, these directions aim not only to refine the current methodology but also to broaden CID’s theoretical and practical footprint. Specifically, the field must transition from single-stage to multi-stage decision-making; move from brittle point estimates to robust identification; progress from exact, heavy surrogates to scalable multi-fidelity optimization; expand from isolated agents to coupled, constraint-laden systems; and shift from stylized demonstrations to reproducible, high-stakes applications. Advancing along these directions will facilitate the development of reliable, deployable incentive designs grounded in causal reasoning.

## Data Availability

Data are contained within the article.

## Abbreviations

The following abbreviations are used in this manuscript:

BO	Bayesian Optimization
CBO	Causal Bayesian Optimization
CID	Causal Incentive Design
CGM	Causal Graphical Models
FCBO	Functional Causal Bayesian Optimization
FBO	Functional Bayesian Optimization
GP	Gaussian Process
MAS	Multi-Agent System
PAP	Principal–Agent Problem
RKHS	Reproducing Kernel Hilbert Space
SCM	Structural Causal Model

## Appendix A. Regret Bound Proofs in a Finite Function Space

Details for the proofs of the propositions needed to prove Theorem 1 are presented here next. Proposition A1 provides a high probability bound on the difference $\left|f\left(\gamma \right)-\mu_{t-1}\left(\gamma \right)\right|$, between the true function value f(γ) and the posterior mean $\mu_{t-1}\left(\gamma \right)$ across the time steps 1,…,T and the incentive functions $\gamma =\{\gamma^{1},\ldots ,\gamma^{T}\}\subseteq \Gamma$ in the finite functional decision space Γ, showing that the posterior mean $\mu_{t-1}\left(\gamma \right)$ is a good estimate of the function value f(γ), with $\gamma =\{\gamma^{1},\ldots ,\gamma^{t-1}\}\subseteq \Gamma$ up to a confidence width of $\sqrt{\beta^{t}}\sigma_{t-1}\left(x\right)$.

**Proposition** **A1.** *Let *Γ *be the finite decision set and δ∈(0,1) be the desired confidence level. Select δ over the open interval (0,1) and set $\beta^{t}=2\log(\frac{\pi^{2}t^{2}}{6\delta }\left|\Gamma |\right)$. Then*

$$\left|f\left(\gamma \right)-\mu^{t-1}\left(\gamma \right)\right|\leq \sqrt{\beta^{t}}\sigma^{t-1}\left(\gamma \right)\;\forall \gamma \in \Gamma ,\forall t\geq 1,$$

*holds with probability greater than or equal to 1−δ.*

**Proof.** We show that for appropriately chosen confidence parameters $\left\{\beta^{t}\right\}$ for t∈[T], every such confidence interval is always valid, with high probability. A random variable X∼N(0,1) has the upper bound of the tail probability $\mathrm{Pr}\left(X>c\right)\leq \frac{1}{2}e^{-\frac{c^{2}}{2}}$ for c>0 (see [31]), and Pr(|X|>c)=2Pr(X>c) as the standard normal distribution is symmetric. For a fixed t≥1, conditioned on observations $y^{t-1}=\left\{{\hat{E}}_{p_{\gamma^{1}}}\left[J_{L}^{1}\right],\ldots ,{\hat{E}}_{p_{\gamma^{\tau }}}\left[J_{L}^{t-1}\right]\right\}$, the sequence of incentive functions $\gamma =\{\gamma^{1},\ldots ,\gamma^{t-1}\}\subseteq \Gamma$ are deterministic and the posterior distribution $f\left(\gamma \right)∼N(\mu^{t-1}\left(\gamma \right),{\sigma^{t-1}}^{2}\left(\gamma \right))$ at time t−1. We normalize this Gaussian as $Z=\frac{f\left(\gamma \right)-\mu^{t-1}\left(\gamma \right)}{\sigma^{t-1}\left(\gamma \right)}∼N\left(0,1\right)$ so we can apply the previous tail bound and symmetry property to *Z*. Therefore, we have the following bound, using X=Z and $c=\sqrt{\beta^{t}}$:

(A1)
$$\mathrm{Pr}\left\{\left|\frac{f\left(\gamma \right)-\mu^{t-1}\left(\gamma \right)}{\sigma^{t-1}\left(\gamma \right)}\right|>\sqrt{\beta^{t}}\right\}=\mathrm{Pr}\left\{\left|f\left(\gamma \right)-\mu^{t-1}\left(\gamma \right)\right|>\sqrt{\beta^{t}}\sigma^{t-1}\left(\gamma \right)\right\}\leq e^{-\frac{\beta^{t}}{2}}.$$

As we want the confidence interval to hold for all γ∈Γ and all time steps t∈[T] in Algorithm 1, we use the union-bound inequality to construct the appropriate parameters $\left\{\beta^{t}\right\}$ for t∈[T]. Let E be the event that at least one confidence interval fails for some γ∈Γ at some time step t∈[T] in the Algorithm 1, so

$$E=\underset{\gamma \in \Gamma ,\;t\in [T]}{⋃}\left|f\left(\gamma \right)-\mu_{t-1}\left(\gamma \right)\right|>\sqrt{\beta^{t}}\sigma^{t-1}\left(\gamma \right).$$

Applying the union bound, we know that

$$\begin{array}{cc} \mathrm{Pr}(E)= & \mathrm{Pr}\left\{\underset{\gamma \in \Gamma ,\;t\in [T]}{⋃}\left|f\left(\gamma \right)-\mu_{t-1}\left(\gamma \right)\right|>\sqrt{\beta^{t}}\sigma^{t-1}\left(\gamma \right)\right\}\\ \leq & \sum_{t\in [T]}\sum_{\gamma \in \Gamma }\mathrm{Pr}\left\{\left|f\left(\gamma \right)-\mu_{t-1}\left(\gamma \right)\right|>\sqrt{\beta^{t}}\sigma^{t-1}\left(\gamma \right)\right\}. \end{array}$$

By running Algorithm 1 up to *T* time steps, we have T|Γ| possibilities that $\left\{\left|f\left(\gamma \right)-\mu_{t-1}\left(\gamma \right)\right|>\sqrt{\beta^{t}}\sigma^{t-1}\left(\gamma \right)\right\}$, and as we want to ensure that Pr(E)≤δ, we can set

(A2)
$$\mathrm{Pr}\left\{\left|f\left(\gamma \right)-\mu_{t-1}\left(\gamma \right)\right|>\sqrt{\beta^{t}}\sigma^{t-1}\left(\gamma \right)\right\}\leq \frac{\delta }{T|\Gamma |}.$$

So, combining this with Equation (A1), we can solve equation $e^{-\frac{\beta^{t}}{2}}=\frac{\delta }{T|\Gamma |}$ for $\beta^{t}$, getting $\beta^{t}=2\log\frac{T|\Gamma |}{\delta }$. Finally, to analyze Algorithm 1 for T→∞, the identity $\sum_{t=1}^{\infty }\frac{1}{t^{2}}=\frac{\pi^{2}}{6}$ is used to design a time-dependent probability budget for each time step and considering the union of infinitely many events. This increases the confidence parameter $\beta_{t}$ over time, so that the probability of failure decreases suitably quickly, and the probability of failure anywhere and at any time is small. So, we now want

$$\mathrm{Pr}\left(E\right)=\mathrm{Pr}\{\overset{\infty }{\underset{t=1}{⋃}}\underset{\gamma \in \Gamma }{⋃}\left\{\left|f\left(\gamma \right)-\mu_{t-1}\left(\gamma \right)\right|>\sqrt{\beta^{t}}\sigma^{t-1}\left(\gamma \right)\right\}\leq \delta .$$

Similar to Equation (A2), but now taking advantage of the identity $\sum_{t=1}^{\infty }\frac{1}{t^{2}}=\frac{\pi^{2}}{6}$, we set

(A3)
$$\mathrm{Pr}\left\{\left|f\left(\gamma \right)-\mu_{t-1}\left(\gamma \right)\right|>\sqrt{\beta^{t}}\sigma^{t-1}\left(\gamma \right)\right\}\leq \frac{\delta }{\left|\Gamma \right|}\cdot \frac{6}{\pi^{2}t^{2}}.$$

Then, by the union bound inequality for this T→∞ case, we have the following:

$$\begin{array}{cc} \mathrm{Pr}(E)\leq & \sum_{t=1}^{\infty }\sum_{\gamma \in \Gamma }\mathrm{Pr}\left\{\left|f\left(\gamma \right)-\mu_{t-1}\left(\gamma \right)\right|>\sqrt{\beta^{t}}\sigma^{t-1}\left(\gamma \right)\right\}\\ \leq & \sum_{\gamma \in \Gamma }\sum_{t=1}^{\infty }\frac{\delta }{\left|\Gamma \right|}\cdot \frac{6}{\pi^{2}t^{2}}=\sum_{\gamma \in \Gamma }\frac{\delta }{\left|\Gamma \right|}\cdot \frac{6}{\pi^{2}}\sum_{t=1}^{\infty }\frac{1}{t^{2}}=\sum_{\gamma \in \Gamma }\frac{\delta }{\left|\Gamma \right|}=\delta \end{array}$$

So, as above, using the tail bound from Equation (A1), we can solve for $\beta_{t}$ the equation $e^{-\frac{\beta^{t}}{2}}=\frac{6\delta }{\pi^{2}t^{2}\left|\Gamma \right|}$, from which we obtain that

(A4)
$$\beta^{t}=2\log(\frac{\pi^{2}t^{2}}{6\delta }\left|\Gamma |\right)$$

□

**Proposition** **A2.**
*For a fixed t≥1. If $\left|f\left(\gamma \right)-\mu^{t-1}\left(\gamma \right)\right|\leq \sqrt{\beta^{t}}\sigma^{t-1}\left(\gamma \right)$ for all $\gamma_{1},\ldots ,\gamma_{t-1}\in \gamma \subseteq \Gamma$, then the instantaneous regret $r_{t}=f\left(\gamma^{*}\right)-f\left(\gamma^{t}\right)\leq 2\sqrt{\beta^{t}}\sigma^{t-1}\left(\gamma^{t}\right)$.*

**Proof.** Algorithm 1 selects the incentive function $\gamma^{t}\in \Gamma$ According to the acquisition functional

$$\gamma^{t}=\underset{\gamma \in \Gamma }{\arg\max}\;\mu^{t-1}\left(\gamma \right)+\beta^{t}\sigma^{t-1}\left(\gamma \right).$$

So, ∀γ∈Γ, $\mu^{t-1}\left(\gamma \right)+\beta^{t}\sigma^{t-1}\left(\gamma \right)\leq \mu^{t-1}\left(\gamma^{t}\right)+\beta^{t}\sigma^{t-1}\left(\gamma^{t}\right)$. In particular, for the unknown optimal incentive function $\gamma^{*}$, we have that

(A5)
$$\mu^{t-1}\left(\gamma^{*}\right)+\beta^{t}\sigma^{t-1}\left(\gamma^{*}\right)\leq \mu^{t-1}\left(\gamma^{t}\right)+\beta^{t}\sigma^{t-1}\left(\gamma^{t}\right).$$

By hypothesis, in the particular cases of $\gamma^{*}$ and $\gamma^{t}$ we have

(A6)
$$f\left(\gamma^{*}\right)\leq \mu^{t-1}\left(\gamma^{*}\right)+\sqrt{\beta^{t}}\sigma^{t-1}\left(\gamma^{*}\right)$$

(A7)
$$f\left(\gamma^{t}\right)\geq \mu^{t-1}\left(\gamma^{t}\right)-\sqrt{\beta^{t}}\sigma^{t-1}\left(\gamma^{t}\right).$$

Then, using inequalities, we have the following bound for instantaneous regret $r^{t}$

$$\begin{array}{cc} r^{t} & =f\left(\gamma^{*}\right)-f\left(\gamma^{t}\right)\\ \; & \leq \mu^{t-1}\left(\gamma^{*}\right)+\sqrt{\beta^{t}}\sigma^{t-1}\left(\gamma^{*}\right)-\left(\mu^{t-1}\left(\gamma^{t}\right)-\sqrt{\beta^{t}}\sigma^{t-1}\left(\gamma^{t}\right)\right)\\ \; & \leq \mu^{t-1}\left(\gamma^{t}\right)+\sqrt{\beta^{t}}\sigma^{t-1}\left(\gamma^{t}\right)-\left(\mu^{t-1}\left(\gamma^{t}\right)-\sqrt{\beta^{t}}\sigma^{t-1}\left(\gamma^{t}\right)\right)\\ \; & =2\sqrt{\beta^{t}}\sigma^{t-1}\left(\gamma^{t}\right) \end{array}$$

□

Proposition A3 shows that the information gain for a selected set of incentive functions can be expressed in terms of the predictive variances and that we can bound this variance in terms of the information gain.

**Proposition** **A3.**
*For any finite sequence of incentive functions $\gamma^{1},\ldots ,\gamma^{\tau }\in \Gamma$*

$$\sum_{t=1}^{\tau }{\left(\sigma^{t}\right)}^{2}\left(\gamma^{t}\right)\leq \frac{\sigma^{2}}{\log(1+\sigma^{-2})}I^{\tau }\left(f^{\tau };y^{\tau }\right).$$

**Proof.** Following Section 4.4, $y^{\tau }=\left\{y^{1},\ldots ,y^{\tau }\right\}=\left\{{\hat{E}}_{p_{\gamma^{1}}}\left[J_{L}^{1}\right],\ldots ,{\hat{E}}_{p_{\gamma^{\tau }}}\left[J_{L}^{\tau }\right]\right\}$, where the incentive functions $\gamma^{1},\ldots ,\gamma^{T}$ are deterministic conditioned on $y^{\tau -1}$. From Equation (63), we know that $I^{\tau }\left(f^{\tau };y^{\tau }\right)=H\left(y^{\tau }\right)-H\left(y^{\tau }|f^{\tau }\right)$. Using the chain rule for differential entropy, we have the following alternative expressions for $H(y^{\tau })$ in Equation (A8) and $H(y^{\tau }|f^{\tau })$ in Equation (A9), equivalent to Equation (63).

(A8)
$$\begin{array}{c} \begin{array}{cc} H(y^{\tau }) & =H(y^{1},\ldots ,y^{\tau })\\ \; & =\sum_{t=1}^{\tau }H\left(y^{t}|y^{1},\ldots ,y^{t-1}\right)\\ \; & =\sum_{t=1}^{\tau }\frac{1}{2}\log\left[2\pi e\left((\sigma^{t-1}{)}^{2}\left(\gamma^{t}\right)+\sigma^{2}\right)\right]. \end{array} \end{array}$$

(A9)
$$H\left(y^{\tau }|f^{\tau }\right)=\sum_{t=1}^{\tau }H\left(\epsilon^{t}\right)=\sum_{t=1}^{\tau }\frac{1}{2}\log\left[\left(2\pi e\right)\sigma^{2}\right],$$

since given $f^{\tau }$, the only randomness left is noise, as $y^{t}={\hat{E}}_{p_{\gamma^{t}}}\left[J_{L}^{t}\right]=f\left(\gamma^{t}\right)+\epsilon^{t}$, with $\epsilon^{t}∼N\left(0,\sigma^{2}\right)$. So, using Equations (A8) and (A9), now we can express the information gain for the selected incentive functions as follows:

(A10)
$$\begin{array}{c} \begin{array}{cc} I^{\tau }\left(f^{\tau };y^{\tau }\right) & =H\left(y^{\tau }\right)-H\left(y^{\tau }|f^{\tau }\right)\\ \; & =\sum_{t=1}^{\tau }\frac{1}{2}\log\left[2\pi e\left((\sigma^{t-1}{)}^{2}\left(\gamma^{t}\right)+\sigma^{2}\right)\right]-\sum_{t=1}^{\tau }\frac{1}{2}\log\left[\left(2\pi e\right)\sigma^{2}\right]\\ \; & =\frac{1}{2}\sum_{t=1}^{\tau }\log\left[\frac{(\sigma^{t-1}{)}^{2}\left(\gamma^{t}\right)+\sigma^{2}}{\sigma^{2}}\right]=\frac{1}{2}\sum_{t=1}^{\tau }\log\left[1+\frac{(\sigma^{t-1}{)}^{2}\left(\gamma^{t}\right)}{\sigma^{2}}\right] \end{array} \end{array}$$

Observe that in Equation (A10), we have expressed the information gain for a selected set of incentive functions exclusively in terms of predictive variances. Now, we use the elementary inequality for all z∈[0,1], $z\leq \frac{1}{\log(1+z^{-1})}\log\left(1+z\right)$, and definite $z^{t}=\frac{{\sigma^{t-1}}^{2}\left(\gamma^{t}\right)}{\sigma^{2}}$ so that ${\sigma^{t-1}}^{2}\left(\gamma^{t}\right)=\sigma^{2}z^{t}$, and then we have

(A11)
$$z^{t}\leq \frac{1}{\log(1+{\left(z^{t}\right)}^{-1})}\log\left(1+z^{t}\right)\leq \frac{1}{\log(1+\sigma^{-2})}\log\left(1+z^{t}\right),$$

because $z^{t}\leq \sigma^{-2}$. Now multiplying both sides of Equation (A11) by $\sigma^{2}$ ans sum over t=1,…,T we have

(A12)
$$\begin{array}{c} \begin{array}{cc} \sum_{t=1}^{T}{\left(\sigma^{t-1}\right)}^{2}\left(\gamma^{t}\right) & \leq \frac{\sigma^{2}}{\log(1+\sigma^{-2})}\sum_{t=1}^{T}\log\left(1+z^{t}\right)\\ \; & =\frac{\sigma^{2}}{\log(1+\sigma^{-2})}\sum_{t=1}^{T}\log\left[1+\frac{(\sigma^{t-1}{)}^{2}\left(\gamma^{t}\right)}{\sigma^{2}}\right]\\ \; & =\frac{\sigma^{2}}{\log(1+\sigma^{-2})}I^{\tau }\left(f^{\tau };y^{\tau }\right) \end{array} \end{array}$$

□

**Proposition** **A4.**
*Let δ∈(0,1) be the desired confidence level and let $\beta^{t}=2\log(\frac{\pi^{2}t^{2}}{6\delta }\left|\Gamma |\right)$. Then, the following holds with probability at least 1−δ:*

(A13)
$$\sum_{t=1}^{T}{\left(r^{t}\right)}^{2}\leq \beta^{T}C_{1}I^{T}\left(f^{T};y^{T}\right)\;\forall T\geq 1,$$

*where $C_{1}=\frac{4\sigma^{2}}{\log(1+\sigma^{-2})}$.*

**Proof.** From Propositions A1 and A2, we have that $\left\{{\left(r^{t}\right)}^{2}\leq 4\beta^{t}{\left(\sigma^{t-1}\right)}^{2}\left(\gamma^{t}\right),\;\forall t\geq 1\right\}$ with probability at least 1−δ, by squaring both sides of expression $r^{t}\leq 2\sqrt{\beta }\sigma^{t-1}\left(\gamma^{t}\right)$ in Proposition A2. So, as for the monotonic increasing property of $\beta^{t}$, $\beta^{t}\leq \beta^{T}$ for all t∈[T] and the result from Proposition A3, we have

(A14)
$$\begin{array}{c} \begin{array}{cc} \sum_{t=1}^{T}{\left(r^{t}\right)}^{2} & \leq 4\sum_{t=1}^{T}\beta^{t}{\left(\sigma^{t-1}\right)}^{2}\left(\gamma^{t}\right)\\ \; & \leq 4\beta^{T}\sum_{t=1}^{T}{\left(\sigma^{t-1}\right)}^{2}\left(\gamma^{t}\right)\\ \; & \leq \beta^{T}\frac{4\sigma^{2}}{\log(1+\sigma^{-2})}I^{\tau }\left(f^{\tau };y^{\tau }\right)\\ \; & \leq \beta^{T}C_{1}I^{\tau }\left(f^{\tau };y^{\tau }\right) \end{array} \end{array}$$

□

Finally, Theorem 1 is a consequence of applying the Cauchy–Schwarz inequality $\sum_{t=1}^{T}r^{t}\leq \sqrt{T\sum_{t=1}^{T}}{\left(r^{t}\right)}^{2}$ to the result in Proposition A4 with which we obtain the following bound:

(A15)
$$R^{T}=\sum_{t=1}^{T}r^{t}\leq \sqrt{T\sum_{t=1}^{T}}{\left(r^{t}\right)}^{2}=\sqrt{T\beta^{T}C_{1}I^{\tau }\left(f^{\tau };y^{\tau }\right)}$$

## Appendix B. Reproducing Kernel Hilbert Spaces

Before describing the notion of Reproducing Kernel Hilbert Space (RKHS) functional space, we discuss kernel functions. Let X be a nonempty set and let $R^{X}$ be the set of all functions of real value on X. Recall that a Hilbert space H is a complete vector space, i.e., all Cauchy sequences converge, with an inner product ${\langle \cdot ,\cdot \rangle }_{H}$ that gives rise to a norm ${∥h∥}_{H}={\langle h,h\rangle }_{H}$ for every h∈H. A function *k*: X×X→R is called a kernel on X if there exist a real Hilbert space H and a map Φ: X→H such that for all $x,x^{\prime }\in X$, we have $k\left(x,x^{\prime }\right)=\langle \Phi \left(x\right),\Phi \left(x^{\prime }\right)\rangle$. We call Φ a feature map and H a feature space of *k*. Furthermore, we must know that a kernel is positive definite in the sense that for all n∈N, $\omega_{1},\ldots ,\omega_{n}\in R$, and all $x_{1},\ldots ,x_{n}\in R$, we have $\sum_{i\in [n]}\sum_{j\in [n]}\omega_{i}\omega_{j}k\left(x_{i},x_{j}\right)\geq 0$. The matrix $K={\left(k\left(x_{j},x_{i}\right)\right)}_{ij}$ is called the Gram matrix, and the positive definiteness of the kernel *k* is equivalent to saying that the determinant of *K* must be non-negative. A functional space RKHS $H_{k}$ in X is a Hilbert space $H\subset R^{X}$ with a kernel function *k*: X×X→H, which is called the reproducing kernel, where for all x∈X and all functions f∈H, we have $f\left(x\right)={\langle f,k_{x}\rangle }_{H}$, where $k_{x}=k\left(\cdot ,x\right)\in H$.

In addition to the definition, a more intuitive approach to understand the rationale underlying an RKHS function space can be achieved through the following construction, which involves the formation of a Hilbert function space (an RKHS) from a kernel *k*: X×X→R. Given X and a function *k*, consider a feature map Φ: $X\to R^{X}$, defined as $x\mapsto \Phi \left(x\right)=k_{x}=k\left(x,\cdot \right)$, known as the reproducing kernel map. That is, the point x∈X is mapped to a function $k_{x}$: X→R, so $k_{x}\left(y\right)=k\left(x,y\right)$ for y∈X. Now, we can construct a vector space by considering the images $\left\{k_{x}|x\in X\right\}$ as a spanning set, i.e., construct a vector space containing all linear combinations of the functions k(·,x). Thus, given a kernel $k(x,x^{\prime })$, the RKHS of functions $H_{k}$ and its inner product are given in Equation (A16) and Equation (A17), respectively.

(A16)
$$H_{k}=\{h\left(\cdot \right)=\sum_{i\in [n]}\omega_{i}k\left(x_{i},\cdot \right)|\omega_{i}\in R,n\in N,x_{i}\in X\},$$

(A17)
$$\langle h,g\rangle =\langle \sum_{i\in [n]}\omega_{i}k\left(x_{i},x\right),\sum_{j\in [m]}\xi_{j}k\left(x_{j}^{\prime },x\right)\rangle =\sum_{i\in [n]}\sum_{j\in [m]}\omega_{i}\xi_{j}k\left(x_{i},x_{j}^{\prime }\right),$$

with $\omega_{i},\xi_{j}\in R$, n,m∈N, $x_{i},x_{j}\in X$. From the inner product defined in Equation (A17), we can deduce the reproducing properties: 〈k(·,x),h〉=h(x), $\langle k\left(\cdot ,x\right),k\left(\cdot ,x^{\prime }\right)\rangle =k\left(x,x^{\prime }\right)$ and we can see that $\langle \Phi \left(x\right),\Phi \left(x^{\prime }\right)\rangle =\langle k\left(\cdot ,x\right),k\left(\cdot ,x^{\prime }\right)\rangle =k\left(x,x^{\prime }\right)$. Hilbert spaces of scalar-valued functions with reproducing kernels were introduced and studied in [32]. The kernel *k* in this appendix is the reproducing kernel on X for $H_{k}$. In the main text, the GP over functions $\Gamma \subset H_{k}$ uses a distinct kernel $K(\gamma ,\gamma^{\prime })$ (see Section 4.1.1), constructed from ${∥\cdot ∥}_{H_{k}}$.

### A Finite Reproducing Kernel Hilbert Space

Here, we provide the construction of the functional space $H_{k}^{d}$ used for the regret analysis for Algorithm 1 in Theorem 1. The most general construction of a RKHS can be achieved using a positive semidefinite kernel defined on a finite set. Let $X=\{x_{1},x_{2},\ldots ,x_{d}\}$ be a finite nonempty set. Let *k*: X×X→R be a symmetric and positive semidefinite kernel function, i.e., for any choice of real coefficients $c_{1},\ldots ,c_{d}$, we have $\sum_{i=1}^{d}\sum_{j=1}^{d}c_{i}c_{j}k\left(x_{i},x_{j}\right)\geq 0$. We have a finite RKHS by defining the function space $H_{k}^{d}$ as the span of kernel sections at the finite points $H_{n}:=\mathrm{span}\left\{k\left(\cdot ,x_{1}\right),k\left(\cdot ,x_{2}\right),\ldots ,k\left(\cdot ,x_{n}\right)\right\}$. So, every function $f\in H_{k}^{d}$ has the form $f\left(x\right)=\sum_{i=1}^{n}c_{i}k\left(x,x_{i}\right)$, for some vector of coefficients $c={\left(c_{1},\ldots ,c_{d}\right)}^{⊤}\in R^{d}$. The Gram matrix is defined as $K\in R^{n\times n}$ by $K_{ij}:=k\left(x_{i},x_{j}\right)$. Since *k* is positive semidefinite, the matrix *K* is symmetric and positive semidefinite. For functions $f,g\in H_{k}^{d}$ defined by

(A18)
$$f\left(x\right)=\sum_{i=1}^{n}c_{i}k\left(x,x_{i}\right),\;g\left(x\right)=\sum_{i=1}^{n}b_{i}k\left(x,x_{i}\right),$$

So, the inner product is given by ${\langle f,g\rangle }_{H_{k}^{d}}:=c^{⊤}Kb$, and the associated norm is given by ${∥f∥}_{H_{k}^{d}}^{2}={\langle f,f\rangle }_{H_{k}^{d}}=c^{⊤}Kc$. The space $H_{k}^{d}$ satisfies the reproducing property $f\left(x_{j}\right)={\langle f,k\left(\cdot ,x_{j}\right)\rangle }_{H_{k}^{d}},\;\forall j=1,\ldots ,d$, because for any $f\in H_{n}$, we have

(A19)
$$f\left(x_{j}\right)=\sum_{i=1}^{d}c_{i}k\left(x_{j},x_{i}\right)={\langle f,k\left(\cdot ,x_{j}\right)\rangle }_{H_{k}^{d}}.$$

The aforementioned construction can be further extended to consider $H_{k}^{d}$ as a finite-dimensional RKHS of a generic kernel, with an infinite and continuous input space $X\subseteq R^{d}$. The key idea is to restrict the RKHS to a finite-dimensional subspace by choosing a finite number of basis functions associated with the kernel.

## Appendix C. Bayesian Optimization

Given a real-valued objective function $f_{obj}$: X→R defined over some domain X, Bayesian optimization (BO) takes a probabilistic approach to methodically search for a point $x^{*}\in \arg\max_{x\in X}\;f_{obj}\left(x\right)$, that achieves the global maximum value $f_{obj}^{*}\left(x\right)=\max_{x\in X}\;f_{obj}\left(x\right)$. It is worth highlighting that we do not presume any assumptions about the characteristics of the domain X. In particular, it is not required to be a Euclidean space; it may be a space with a more complex structure, such as a function space, which is the focus of our investigation. In addition, BO differs from traditional optimization, as it does not require $f_{obj}\left(x\right)$ to have a known restricted functional form. BO is an instance of the more general framework of sequential optimization where the input is an initial (possibly empty) data set D={(x,y)} from which we want to design a sequence of experiments to gather information about $f_{obj}\left(x\right)$. In each interaction, a policy optimizer selects a point $x^{\alpha }$ where we make our next observation. Having selected the point $x^{\prime }=x^{\alpha }$, we can obtain feedback from the system under study to obtain $f_{obj}\left(x^{\prime }\right)$. We append the newly observed information to update the data set, i.e., $D^{\prime }=D\cup \left\{\left(x^{\prime },y^{\prime }\right)\right\}$. We repeat until the termination condition is reached. There are different mechanisms by which the decision is made to terminate the sequential optimization process. For our purpose in this research, we assume a stopping criteria by exhausting an experimentation budget, that is, a finite time horizon T∈N. Also, we discard the strong assumption of having an exact observation model and instead adopt an additive Gaussian noise observation model, i.e., the value $f_{obj}\left(x\right)$ observed at *x* modeled as $y=f_{obj}\left(x\right)+\epsilon$, where $\epsilon ∼N(0,\sigma^{2})$ represents the measurement error.

What distinguishes BO from the more general sequential optimization framework is that BO relies on Bayesian inference (Bayes’ Theorem) to reason about the uncertain quantities of a system of interest in light of our prior knowledge and any available data. This specially includes the objective function $f_{obj}$, which is typically treated as a stochastic process in this context, i.e., a probability distribution over an infinite family of random variables $y=f_{obj}\left(x\right)+\epsilon$ in a probability space, indexed by points x∈X.

### Appendix C.1. Gaussian Process Surrogate Model

We focus on the Gaussian process (GP) representation of the objective function $f_{obj}$. A GP is a stochastic process such that every finite collection of random variables in the stochastic process has a multivariate normal distribution. The distribution of a GP is the joint distribution of all those (infinitely many) random variables, and as such it is a distribution over functions with a continuous domain. Thus, a GP can be used as a prior probability distribution over functions to perform Bayesian inference, i.e., to compute posterior distribution refining our initial beliefs according to the observed data.

We specify a Gaussian process as GP(μ(·),k(·,·)) with mean function μ and covariance kernel *k* that is presumably bounded: $k(x,x^{\prime })\leq 1$, for all x∈X, without loss of generality. The mean function $\mu \left(x\right)=E\left(f_{obj}|x\right)$ determines the expected value of our model for $f_{obj}$ at any location *x*, thus serving as a location parameter that represents the central tendency of our stochastic model for $f_{obj}\left(x\right)$. On the other hand, the covariance kernel $k\left(x,x^{\prime }\right)=cov\left[f_{obj},f_{obj}^{\prime }|x,x^{\prime }\right]$ determines how deviations from the mean are structured in our model for $f_{obj}$, i.e., for its samples $f_{obj}\left(x\right)∼\mathrm{GP}\left(\mu \left(x\right),k\left(x,\cdot \right)\right)$, encoding also expected properties for our stochastic model of $f_{obj}$ such as differentiability and smoothness. The squared exponential, also known as the Radial Basis Function (RBF) kernel, and the kernels of the Matérn family of kernels, are two examples of frequently used kernels in the GP framework.

### Appendix C.2. Acquisition Functions

Every BO method fundamentally has two major components: (i) First, a probabilistic surrogate model to represent the belief over the black-box objective function $f_{obj}$, as the Gaussian processes previously discussed. (ii) Second, an acquisition function (AF), α: X→R, which establishes a utility value for the candidate evaluation points from the posterior distribution of the probabilistic surrogate model to select the next optimal evaluation point. Specifically, an acquisition function α: X→R assigns a score to each point in the domain reflecting our preferences over locations for the next observation.

Even though there is a large collection of acquisition functions, we restrict our attention to the Gaussian Process Upper Confidence Bound (GP-UCB) acquisition function. The Upper Confidence Bound criterion is commonly used to deal with the exploration-exploitation dilemma and to evaluate strategies in Multi-Armed Bandit (MAB) algorithms. The GP-UCB acquisition function is an extension of the UCB criteria on MAB to BO, as BO can be interpreted as an infinite-armed bandit problem. Assume we have gathered an arbitrary data set D, and consider an arbitrary point x∈X. Consider the quantile function associated with the predictive distribution p(y∣x,D) given by

$$q\left(\rho ;x,D\right)=\inf\{y^{\prime }|\mathrm{Pr}\left(y\leq y^{\prime }|x,D\right)\geq \rho \}.$$

This quantile function satisfies the relation y≤q(ρ;x,D) with probability ρ∈(0,1), so we can interpret q(ρ;x,D) as an Upper Confidence Bound (UCB) on the objective function *y*, i.e., say that the value of *y* will exceed the bound only with tunable probability 1−ρ. As a function of *x*, we can elucidate q(ρ;x,D) as an optimistic estimate of *y*, suggesting that we observe where this Upper Confidence Bound is maximized, resulting in the acquisition function $\alpha_{\mathrm{UCB}}\left(x;D,\rho \right)=q\left(\rho ;x,D\right)$, where *q* is the quantile function with confidence ρ∈(0,1). For a Gaussian process, this quantile takes the simple form of Equation (A20):

(A20)
$$\alpha_{\text{GP-UCB}}\left(x;D,\rho \right)=\mu \left(x\right)+\beta \sigma \left(x\right),$$

where $\beta =\Phi^{-1}\left(\rho \right)$ depends on the confidence level and can be computed from the inverse Gaussian cumulative distribution function ( $\Phi^{-1}$ denotes the quantile function, i.e., the inverse cumulative distribution function (CDF)). Employing low confidence values (e.g., ρ=0.8) diminishes recognition for sites with high uncertainty, hence favoring exploitation substantially. Increasing the confidence parameter (e.g., ρ=0.95 or ρ=0.99) leads to increasingly exploratory behavior. The policy implemented by sequentially maximizing Upper Confidence Bounds is backed by robust theoretical guarantees. Srinivas et al. [7] demonstrated that this policy is guaranteed to effectively maximize the objective at a non-trivial rate for GP models under reasonable assumptions. In the main text, (Section 4.2 and Section 4.3), we parameterize the acquisition by a (possibly time-varying) exploration coefficient $\beta^{t}$. For the regret analysis (Section 4.4), we take $\beta^{t}=2\log\;\left(\frac{t^{2}\pi^{2}\left|\Gamma \right|}{6\delta }\right)$, which is equivalent to using a time-varying confidence level $\rho^{t}=\Phi \left(\beta^{t}\right)$.

## Appendix D. Functional Approximation via the Stone–Weierstrass Theorem

The Stone–Weierstrass theorem provides sufficient conditions for approximating continuous functions on compact spaces using elements from a subalgebra. In its classical real-valued formulation, it characterizes when an algebra of continuous functions is uniformly dense in the full space C(X,R), the Banach space of real-valued continuous functions on a compact Hausdorff space *X*, endowed with the uniform norm (see, for instance, ref. [33]).

Let (X,d) be a compact Hausdorff space and let C(X,R) denote the space of continuous real-valued functions on *X*, equipped with the uniform norm

$${∥g∥}_{\infty }:=\underset{x\in X}{\sup}\left|g\left(x\right)\right|.$$

A subset A⊂C(X,R) is called an algebra if it is closed under pointwise addition, scalar multiplication, and pointwise multiplication. We say that *A* separates points of *X* if for every pair of distinct points x,y∈X, there exists g∈A such that g(x)≠g(y). This means the algebra is rich enough to distinguish any two points of the domain. We say that *A* contains the constants if the constant function 1(x)≡1 belongs to *A*.

**Theorem** **A1** (Stone–Weierstrass Theorem)**.** *If A⊂C(X,R) is a subalgebra that separates points of X and contains the constants, then A is dense in C(X,R) with respect to the uniform norm. That is, for every f∈C(X,R) and every ε>0, there exists g∈A such that*

$${∥f-g∥}_{\infty }<\varepsilon .$$

In our context, *X* will be either the compact subset $\Gamma \subset H_{k}$ of admissible incentive functions, or the compact input domain $\Omega_{F}$ of a follower’s decision variable. The algebra *A* will be constructed from either (1) the span of kernel sections $K(\cdot ,\gamma_{0})$ with $\gamma_{0}\in \Gamma$ for the approximation of the functional *f*: Γ→R, or (2) the span of kernel sections k(·,x) with $x\in \Omega_{F}$ or the polynomial algebra $P_{n}$ for the approximation of scalar-valued incentive functions γ: $\Omega_{F}\to R$.

The separation property follows from the positive definiteness of the kernels *K* or *k*, which guarantees that kernel sections distinguish distinct points. The constants are contained in the algebra because constant functions can be obtained (or approximated) from kernel sections or are trivially included in $P_{n}$.

Theorem A1 then ensures that any continuous target functional or incentive function can be uniformly approximated by finite linear combinations of functions from these algebras, which is the foundation for the two approximation steps described in the following subsections.

### Appendix D.1. Approximation of the Objective Functional

Let $G\subset H_{k}$ be a compact subset of the RKHS of incentive functions, equipped with the topology induced by the RKHS norm ${∥\cdot ∥}_{H_{k}}$. That is, a sequence $\left\{\gamma_{n}\right\}\subset H_{k}$ converges to $\gamma \in H_{k}$ if and only if ${∥\gamma_{n}-\gamma ∥}_{H_{k}}\to 0$. We consider the objective functional *f*: G→R defined by

$$f\left(\gamma \right)=E[J_{L}|\mathrm{do}\left(\Omega_{L}=\gamma \left(\omega_{F}\right)\right)],$$

which we assume to be continuous with respect to ${∥\cdot ∥}_{H_{k}}$. The functional kernel *K*: $H_{k}\times H_{k}\to R$ induces a family of evaluation maps of the form $\gamma \mapsto K(\gamma ,\gamma_{0})$ for fixed $\gamma_{0}\in G$. We define the algebra

$$A_{1}:=\mathrm{span}\left\{K\left(\cdot ,\gamma_{0}\right)\;|\;\gamma_{0}\in G\right\}\subset C\left(G,R\right).$$

This algebra separates points, since *K* is strictly positive definite and for $\gamma_{1}\neq \gamma_{2}$, there exists $\gamma_{0}$ such that $K\left(\gamma_{1},\gamma_{0}\right)\neq K\left(\gamma_{2},\gamma_{0}\right)$. Also, constants functions are included in this algebra, since K(γ,γ) is bounded below by a positive value and adding scaled copies yields constant approximations. Hence, according to the Stone–Weierstrass theorem, $A_{1}$ is uniformly dense in C(G,R), and any continuous objective functional *f* can be approximated by elements of the algebra $A_{1}$. The algebra described above is equivalent to the following algebra

We can rewrite this $A_{1}$ in the language that we need for regret bounds. Let $\Gamma \subset H_{k}$ be a compact subset of the RKHS of incentive functions, endowed with the norm topology ${∥\cdot ∥}_{H_{k}}$. We assume that the objective functional *f*: Γ→R is continuous, f∈C(Γ). We rewrite this algebra $A_{1}$ as follows:

$$A_{1}:=\mathrm{span}\left\{K\left(\cdot ,\gamma_{0}\right)\;|\;\gamma_{0}\in \Gamma \right\},$$

where *K*: $H_{k}\times H_{k}\to R$ is the functional kernel used in the GP prior. Because *K* is positive definite, $A_{1}$ separates points in Γ and contains constant functions, as stated above. By the Stone–Weierstrass theorem, $A_{1}$ is dense in C(Γ) under the uniform norm.

So, as a consequence, for every $\varepsilon_{1}>0$, there exists a finite set $\{\gamma_{1},\ldots ,\gamma_{d}\}\subset \Gamma$ and coefficients $\alpha_{1},\ldots ,\alpha_{d}\in R$ such that

$$\underset{\gamma \in \Gamma }{\sup}\left|f\left(\gamma \right)-\sum_{i=1}^{d}\alpha_{i}\;K\left(\gamma_{i},\gamma \right)\right|<\varepsilon_{1}.$$

Therefore, the finite set ${\left\{\gamma_{i}\right\}}_{i=1}^{d}$ plays the role of a basis of reference incentive functions. Each γ∈Γ is represented through its coordinates $\left(K\left(\gamma_{1},\gamma \right),\ldots ,K\left(\gamma_{d},\gamma \right)\right)$ in this basis. The approximant $\sum_{i=1}^{d}\alpha_{i}K\left(\gamma_{i},\gamma \right)$ is a finite-dimensional parametric model for *f*, with parameters $\left\{\alpha_{i}\right\}$. Thus, the algebraic density statement of Stone–Weierstrass and the finite-basis parametric view are equivalent; both state that a continuous functional *f* in Γ can be uniformly approximated by an element of the finite-dimensional span of kernel sections $K(\cdot ,\gamma_{i})$. This is the first step in reducing the infinite-dimensional functional optimization problem to one over a finite parameter space.

### Appendix D.2. Selection Methods of a Finite Set of Basis Incentive Functions

In practice, the first application of the Stone–Weierstrass theorem requires the construction of a finite set $\left\{\gamma_{1},\ldots ,\gamma_{d}\right\}\subset H_{k}$ such that the algebra they generate, $A_{1}=\mathrm{span}\left\{K\left(\cdot ,\gamma_{i}\right):i=1,\ldots ,d\right\}$, separates points and contains the constants, ensuring density in $C(H_{k},R)$ under the uniform norm on compact subsets. Below, we describe two formal approaches for constructing such a set.

Method 1: ε-cover-based selection. Let $\Gamma \subset H_{k}$ be compact in the RKHS norm ${∥\cdot ∥}_{H_{k}}$. For ε>0, an *ε-cover* of Γ with respect to the $H_{k}$ norm is a finite subset $G_{\varepsilon }\subset \Gamma$ such that for every γ∈Γ, there exists $\tilde{\gamma }\in G_{\varepsilon }$ satisfying ${∥\gamma -\tilde{\gamma }∥}_{H_{k}}\leq \varepsilon .$ An ε-cover is said to be minimal if it has the smallest possible cardinality among all ε-covers of Γ. The minimal cardinality is called the *ε-covering number* and is denoted by $N(\Gamma ,{∥\cdot ∥}_{H_{k}},\varepsilon )$.

Given ε>0, a minimal ε-cover can be constructed by iteratively selecting points from Γ such that (i) the first point is chosen arbitrarily from Γ, (ii) each subsequent point is chosen so that it lies at $H_{k}$-norm distance strictly greater than ε from all previously chosen points, and (iii) the process stops once the selected points cover Γ in the sense above. This greedy selection achieves a covering whose cardinality is within a factor of optimal for compact metric spaces. *Basis selection.* Once a minimal (or near-minimal) ε-cover $G_{\varepsilon }=\left\{{\tilde{\gamma }}_{1},\ldots ,{\tilde{\gamma }}_{d}\right\}$ is obtained, we set $\left\{\gamma_{1},\ldots ,\gamma_{d}\right\}:=G_{\varepsilon }$. By properties of continuous positive-definite kernels *K*, the kernel sections $K(\cdot ,\gamma_{i})$ separate points in Γ and constants are contained in $A_{1}=\mathrm{span}\left\{K\left(\cdot ,\gamma_{i}\right)\right\}$.

By the Stone–Weierstrass theorem, $A_{1}$ is then dense in C(Γ,R) in the uniform topology (see, for instance, [27]). This method explicitly links the choice of basis to covering numbers $N(\Gamma ,{∥\cdot ∥}_{H_{k}},\varepsilon )$, which determines the discretization size $|G_{\varepsilon }|=d$, which are also relevant in the analysis of the confidence parameter $\beta_{T}$ in the regret bounds.

Method 2: Spectral decomposition of the kernel operator. Assume that *K*: Γ×Γ→R is continuous and positive-definite on a compact Γ with associated Mercer decomposition

$$K\left(\gamma ,\gamma^{\prime }\right)=\sum_{m=1}^{\infty }\lambda_{m}\;\phi_{m}\left(\gamma \right)\;\phi_{m}\left(\gamma^{\prime }\right),$$

where ${\left(\lambda_{m}\right)}_{m\geq 1}$ are positive eigenvalues and ${\left(\phi_{m}\right)}_{m\geq 1}$ are orthonormal eigenfunctions in $L^{2}\left(\Gamma ,\mu \right)$ for some finite Borel measure μ. Select $\gamma_{i}\in \Gamma$ such that $\gamma_{i}$ corresponds to the RKHS representative associated with $\phi_{i}$, and take *d* large enough so that $\sum_{m>d}\lambda_{m}$ is below a desired threshold. The kernel sections $K(\cdot ,\gamma_{i})$ inherit the properties of separation and constant inclusion, and the span of the first *d* eigenfunctions produces a finite-dimensional approximation space whose richness improves with *d*. For details on Mercer expansions and spectral selection strategies, see [34].

In both methods, the returned finite set $\left\{\gamma_{1},\ldots ,\gamma_{d}\right\}$ serves as a basis for the algebra $A_{1}$, ensuring that the approximation property required by the Stone–Weierstrass theorem holds to within the desired tolerance on compact subsets of $H_{k}$.
